# Supplementary material for: Diversification dynamics in the Neotropics through time, clades, and biogeographic regions
Source: eLife. 2022 Oct 27;11:e74503. doi: 10.7554/eLife.74503 (PMC9668338; doi:10.7554/eLife.74503)
Supplement: Figure 2—source data 1. [file elife-74503-fig2-data1.docx]

**Figure 2, source data file 1**.

Dataset of plant (P), mammal (M), bird (B), squamate (S) and amphibian (A) phylogenies compiled in this study, including the taxonomic level (Tax. level), the crown age (in million years ago), the number of species on the clade (#spp), the number of Neotropical species (#spp Neotrop) and proportion, as well as the sampling fraction (*i.e.* the number of species sampled in the tree from the total number of species described in the group).

| **ID** | **Order** | **Family** | **Clade name** | **Tax. level** | **Crown Age** | **#spp** | **#spp**  **Neotrop** | **%**  **Neotrop** | **#spp**  **sampled** | **Samplig**  **fraction** | **Tree origin** | **#spp reference** |
| --- | --- | --- | --- | --- | --- | --- | --- | --- | --- | --- | --- | --- |
| P1 | Asparagales | Orchidaceae | *Prosthechea* | genus | 2,88 | 112 | 112 | 100,00 | 13 | 0,12 | (1) | (2) |
| P2 | Asparagales | Orchidaceae | *Camaridium* | genus | 0,93 | 140 | 140 | 100,00 | 39 | 0,28 | (1) | (2) |
| P3 | Zingiberales | Zingiberaceae | *Renealmia* | genus | 43,02 | 76 | 61 | 80,26 | 12 | 0,16 | (1) | (3) |
| P4 | Zingiberales | Costaceae | *Costus*_clade 1 | section | 13,8 | 80 | 40 | 50,00 | 10 | 0,13 | (1) | (4) |
| P5 | Zingiberales | Costaceae | *Costus*_clade 2 | section | 7,32 | 80 | 40 | 50,00 | 10 | 0,13 | (1) | (4) |
| P6 | Magnoliales | Annonaceae | *Cremastosperma* | genus | 5,81 | 31 | 31 | 100,00 | 14 | 0,45 | (1) | (5) |
| P7 | Magnoliales | Annonaceae | Annonaceae-clade-2 | genus | 12,27 | 51 | 51 | 100,00 | 29 | 0,57 | (1) | (5) |
| P8 | Myrtales | Onagraceae | *Fuchsia* | genus | 11,87 | 108 | 103 | 95,37 | 15 | 0,14 | (1) | (6) |
| P9 | Myrtales | Melastomataceae | Melastomataceae-clade-1 | tribe | 19,62 | 72 | 72 | 100,00 | 11 | 0,15 | (1) | (7) |
| P10 | Rosales | Moraceae | Castilleae group | tribe | 25,52 | 59 | 59 | 100,00 | 24 | 0,41 | (1) | (2, 8) |
| P11 | Fabales | Fabaceae | *Andira*-clade | genus | 15,15 | 44 | 43 | 97,73 | 30 | 0,68 | (9) | (9) |
| P12 | Caryophyllales | Polygonaceae | Polygonaceae-clade-1 | genus | 13,04 | 43 | 43 | 100,00 | 15 | 0,35 | (1) | (2) |
| P13 | Ericales | Sapotaceae | *Sideroxylon* | genus | 56,3 | 81 | 49 | 60,49 | 26 | 0,32 | (10) | (2) |
| P14 | Ericales | Lecythidaceae | Lecythidaceae-clade-1 | genus | 26 | 50 | 50 | 100,00 | 10 | 0,20 | (1) | (2) |
| P15 | Ericales | Lecythidaceae | *Lecythis* | genus | 19,87 | 27 | 27 | 100,00 | 17 | 0,63 | (1) | (2, 11) |
| P16 | Ericales | Lecythidaceae | *Eschweilera* | genus | 7 | 92 | 92 | 100,00 | 19 | 0,21 | (1) | (2, 11) |
| P17 | Gentianales | Rubiaceae | Condamineeae | genus | 22,42 | 99 | 99 | 100,00 | 18 | 0,18 | (1) | (2) |
| P18 | Fabales | Fabaceae | *Amicia* | genus | 10,8 | 8 | 8 | 100,00 | 7 | 0,88 | (12) | (2) |
| P19 | Gentianales | Rubiaceae | Cinchoneideae-clade | Genus | 28,06 | 122 | 117 | 95,90 | 22 | 0,18 | (13) | (2) |
| P20 | Fabales | Fabaceae | *Coursetia* | Genus | 20,25 | 40 | 40 | 100,00 | 29 | 0,73 | (14) | (2) |
| P21 | Magnoliales | Annonaceae | *Guatteria* | genus | 20,76 | 254 | 254 | 100,00 | 103 | 0,41 | (15) | (2) |
| P22 | Chloranthales | Chlorantaceae | *Hedyosmum* | genus | 17,63 | 44 | 44 | 100,00 | 19 | 0,43 | (16) | (2) |
| P23 | Gentianales | Rubiaceae | Isertieae | tribe | 25,05 | 16 | 16 | 100,00 | 9 | 0,56 | (13) | (2) |
| P24 | Caryophyllales | Campanulaceae | Centropogonid_clade | tribe | 17 | 550 | 550 | 100,00 | 200 | 0,36 | (17) | (17) |
| P25 | Cycadales | Zamiaceae | *Ceratozamia* | genus | 19,2 | 27 | 27 | 100,00 | 24 | 0,89 | (18) | (18) |
| P26 | Cycadales | Zamiaceae | *Zamia* | genus | 14,63 | 71 | 71 | 100,00 | 43 | 0,61 | (18) | (18) |
| P27 | Fabales | Fabaceae | *Lupinus*-clade-1 | subgenus | 4,67 | 35 | 35 | 100,00 | 19 | 0,54 | (19) | (19) |
| P28 | Fabales | Fabaceae | *Lupinus*-clade-2 | subgenus | 2,75 | 130 | 130 | 100,00 | 37 | 0,28 | (19) | (19) |
| P29 | Lamales | Bignoniaceae | *Tynanthus* | genus | 15,28 | 15 | 15 | 100,00 | 14 | 0,93 | (20) | (2) |
| P30 | Arecales | Arecaceae | *Sabal* | genus | 37,32 | 16 | 16 | 100,00 | 14 | 0,88 | (21) | (2) |
| P31 | Arecales | Arecaceae | Cryosophileae | tribe | 30,96 | 80 | 80 | 100,00 | 78 | 0,98 | (21) | (2) |
| P32 | Arecales | Arecaceae | *Copernicia* | genus | 20,51 | 28 | 28 | 100,00 | 22 | 0,79 | (21) | (2) |
| P33 | Arecales | Arecaceae | *Livistoninae*-clade-1 | tribe | 19,96 | 16 | 16 | 100,00 | 16 | 1,00 | (21) | (2) |
| P34 | Arecales | Arecaceae | Iriarteeae | tribe | 29,67 | 32 | 32 | 100,00 | 32 | 1,00 | (21) | (2) |
| P35 | Arecales | Arecaceae | Chamaedoreeae | tribe | 38,07 | 115 | 115 | 100,00 | 114 | 0,99 | (21) | (2) |
| P36 | Arecales | Arecaceae | Attaleinae | tribe | 27,09 | 156 | 156 | 100,00 | 154 | 0,99 | (21) | (2) |
| P37 | Arecales | Arecaceae | Elaeidinae+Bactridinae | tribe | 43,62 | 180 | 180 | 100,00 | 178 | 0,99 | (21) | (2) |
| P38 | Arecales | Arecaceae | Geonomateae | tribe | 26,92 | 102 | 102 | 100,00 | 102 | 1,00 | (21) | (2) |
| P39 | Arecales | Arecaceae | Euterpeae | tribe | 34,2 | 22 | 22 | 100,00 | 22 | 1,00 | (21) | (2) |
| P40 | Fabales | Fabaceae | *Piptadenia*-group | genus | 20,39 | 594 | 554 | 93,27 | 271 | 0,46 | (9) | (22) |
| P41 | Asparagales | Orchidaceae | Pleurothallidinae | tribe | 18,34 | 5100 | 5100 | 100,00 | 670 | 0,13 | (23) | (23) |
| P42 | Asparagales | Orchidaceae | Cymbidieae | tribe | 34,7 | 3700 | 3330 | 90,00 | 789 | 0,21 | (23) | (23) |
| P43 | Poales | Poaceae (Panicoideaea) | Paspaleae | tribe | 21 | 680 | 680 | 100,00 | 168 | 0,25 | (24) | (2) |
| P44 | Fabales | Fabaceae | *Inga* | genus | 6 | 381 | 381 | 100,00 | 126 | 0,33 | (25) | (2) |
| P45 | Lamiales | Gesneriaceae | Gesnerioideae | tribe | 48,44 | 1200 | 1200 | 100,00 | 588 | 0,49 | (26) | (26) |
| P46 | Sapindales | Burseraceae | Proteiae | tribe | 28,91 | 140 | 134 | 95,71 | 111 | 0,79 | (27) | (27) |
| P47 | Solanales | Solanaceae | Schizanthoideae-Goetzeoideae | tribe | 28,8 | 21 | 20 | 95,24 | 21 | 1,00 | (28, 29) | (28, 29) |
| P48 | Solanales | Solanaceae | Cestroideae | tribe | 32,21 | 221 | 221 | 100,00 | 50 | 0,23 | (28, 29) | (28, 29) |
| P49 | Solanales | Solanaceae | Petunioideae | tribe | 30,92 | 146 | 146 | 100,00 | 44 | 0,30 | (28, 29) | (28, 29) |
| P50 | Solanales | Solanaceae | Nolaneae | tribe | 7,2 | 89 | 89 | 100,00 | 63 | 0,71 | (28, 29) | (28, 29) |
| P51 | Solanales | Solanaceae | Physaleae | tribe | 21,78 | 448 | 371 | 82,81 | 198 | 0,44 | (28, 29) | (28, 29) |
| P52 | Solanales | Solanaceae | Solaneae | tribe | 20,79 | 1550 | 1000 | 64,52 | 495 | 0,32 | (28, 29) | (30) |
| P53 | Fabales | Detarioideae | *Brownea*-clade | tribe | 30,27 | 111 | 111 | 100,00 | 86 | 0,78 | (31) | (31) |
| P54 | Lamiales | Plantaginaceae | Angeloniae | tribe | 45,01 | 68 | 68 | 100,00 | 38 | 0,56 | (32) | (32) |
| P55 | Asparagales | Orchidaceae | Catasetinae | tribe | 19,5 | 262 | 262 | 100,00 | 120 | 0,46 | (23) | (2) |
| P56 | Myrtales | Myrtaceae | *Myrcia* s.l. | genus | 27,87 | 690 | 690 | 100,00 | 173 | 0,25 | (33) | (34) |
| P57 | Dioscoreales | Dioscoreaceae | Dioscoreaceae | family | 48,28 | 637 | 321 | 50,39 | 161 | 0,25 | (35) | (2, 35) |
| P58 | Malpighiales | Euphorbiaceae | *Croton* | genus | 42,54 | 1300 | 900 | 69,23 | 312 | 0,24 | (36) | (36, 37) |
| P59 | Magnoliales | Annonaceae | *Duguetia*-*Fusaea* | genus | 24,16 | 97 | 93 | 95,88 | 34 | 0,35 | (1) | (2) |
| P60 | Brassicales | Tropaeolaceae | *Tropaeolum* | genus | 35,74 | 88 | 88 | 100,00 | 16 | 0,18 | (1) | (2) |
| P61 | Malvales | Bombacoideae | *Eriotheca*-*Pachira* | genus | 8,68 | 110 | 109 | 99,09 | 14 | 0,13 | (1) | (2) |
| P62 | Sapindales | Simaroubaceae | *Simaba* | genus | 1,64 | 25 | 25 | 100,00 | 10 | 0,40 | (1) | (2) |
| P63 | Fabales | Fabaceae | *Leucaena* | genus | 0,87 | 24 | 24 | 100,00 | 10 | 0,42 | (1) | (2) |
| P64 | Caryophyllales | Cactaceae | *Pereskia* | genus | 4,56 | 17 | 17 | 100,00 | 10 | 0,59 | (1) | (2) |
| P65 | Ericales | Symplocaceae | *Symplocos* | genus | 21,5 | 300 | 105 | 35,00 | 32 | 0,11 | (1) | (38) |
| P66 | Gentianales | Apocynaceae | *Mandevilla* | genus | 0,49 | 174 | 174 | 100,00 | 48 | 0,28 | (1) | (2) |
| M1 | Xenarthra | Xenarthra | Xenarthra | order | 67,95 | 32 | 32 | 100 | 32 | 1,00 | (39) | (40) |
| M2 | Chiroptera | Phyllostomidae | Phyllostomidae | family | 48,47 | 194 | 194 | 100 | 194 | 1,00 | (41) | (40) |
| M3 | Chiroptera | Molossidae | Molossini | subgenus | 22,5 | 29 | 29 | 100 | 20 | 0,69 | (42) | (40) |
| M4 | Platyrrhini | Ceboidea | Ceboidea | superfam | 21,53 | 199 | 199 | 100 | 95 | 0,48 | (43) | (44) |
| M5 | Didelphimorphia | Didelphidae | Didelphidae | Family | 25,49 | 103 | 92 | 90 | 43 | 0,42 | (45) | (40) |
| M6 | Rodentia (Myomorpha) | Cricetidae | Sigmodontinae | subfam | 12,65 | 413 | 400 | 96,85 | 279 | 0,68 | (46) | (40) |
| M7 | Rodentia (Castorimorpha) | Heteromyidae | *Heteromys* | genus | 10,6 | 16 | 16 | 100 | 11 | 0,69 | (42) | (40) |
| M8 | Rodentia (Myomorpha) | Cricetidae | Neotominae-*Reithrodontomys* | genus | 13,8 | 22 | 18 | 81,82 | 10 | 0,45 | (42) | (40) |
| M9 | Chiroptera | Emballonuroidea | Diclidurini | tribe | 34,9 | 22 | 22 | 100 | 20 | 0,91 | (42) | (47) |
| M10 | Artiodactyla | Cervidae | Odocoileini | tribe | 8,54 | 19 | 17 | 89,47 | 11 | 0,58 | (48) | (40) |
| M11 | Carnivora | Mephitidae | New World Mephitidae | family | 16 | 10 | 10 | 100 | 10 | 1,00 | (49) | (40) |
| M12 | Rodentia (Caviomorpha) | Caviomorpha | Caviomorpha | parvorder | 35,27 | 244 | 244 | 100 | 199 | 0,82 | This study | (50) |
| B1 | Galliformes | Cracidae | Cracidae | family | 11,82 | 55 | 55 | 100 | 39 | 0,71 | (51) | (52) |
| B2 | Apodiformes | Trochilidae | Hummingbirds | family | 26,09 | 338 | 338 | 100 | 233 | 0,69 | (51) | (53) |
| B3 | Falconiformes | Falconidae | Caracarinae+*Spiziapteryx* | Subfam | 24,78 | 11 | 11 | 100 | 8 | 0,73 | (51) | (52) |
| B4 | Psittaciformes | Psittacidae | Psittacidae | Family | 28,95 | 167 | 157 | 94,01 | 118 | 0,71 | (51) | (52) |
| B5 | Passeriformes | Grallariidae-Rhinocryptidae | Grallariidae-Rhinocryptidae | family | 25,16 | 112 | 112 | 100 | 55 | 0,49 | (51) | (52) |
| B6 | Passeriformes | Melanopareiidae-Conopophagidae | Melanopareiidae-Conopophagidae | family | 23,42 | 15 | 15 | 100 | 9 | 0,60 | (51) | (52) |
| B7 | Passeriformes | Formicariidae | Formicariidae | family | 18,54 | 11 | 11 | 100 | 7 | 0,64 | (51) | (52) |
| B8 | Passeriformes | Thamnophilidae | Thamnophilidae | family | 16,82 | 234 | 234 | 100 | 165 | 0,71 | (51) | (52) |
| B9 | Passeriformes | Funariidae | Funariidae | family | 32,58 | 301 | 301 | 100 | 284 | 0,94 | (54) | (52) |
| B10 | Passeriformes | Tyrannoidea | Tyrannoidea | superfam | 25,5 | 400 | 400 | 100 | 316 | 0,79 | (51) | (52) |
| B11 | Passeriformes | Pipridae-Cotingidae | Pipridae-Cotingidae | family | 23,51 | 118 | 118 | 100 | 80 | 0,68 | (51) | (52) |
| B12 | Passeriformes | Vireonidae | Vireonidae | family | 20,15 | 52 | 42 | 80,77 | 19 | 0,37 | (51) | (55) |
| B13 | Passeriformes | Corvidae | Neot-Corvidae | family | 17,38 | 38 | 36 | 94,74 | 36 | 0,95 | (51) | (52) |
| B14 | Passeriformes | Turdidae | *Turdus* | genus | 8,19 | 46 | 44 | 95,65 | 39 | 0,85 | (51) | (52) |
| B15 | Passeriformes | Turdidae | Neotrop-Turdidae | genus | 13,10 | 18 | 17 | 94,44 | 17 | 0,94 | (51) | (52) |
| B16 | Passeriformes | Certhioidea | Polioptilidae-Troglodytidae | family | 24,50 | 108 | 100 | 92,59 | 67 | 0,62 | (51) | (52) |
| B17 | Passeriformes | Fringillidae | *Euphonia* | genus | 13,1 | 27 | 27 | 100 | 10 | 0,37 | (51) | (52) |
| B18 | Passeriformes | Passerellidae | Passerellidae | family | 11,2 | 90 | 60 | 66,67 | 89 | 0,99 | (51) | (52) |
| B19 | Passeriformes | Icteridae | Icteridae | family | 12,02 | 103 | 103 | 100 | 92 | 0,89 | (51) | (52) |
| B20 | Passeriformes | Cardinalidae | Cardinalidae | family | 18,03 | 50 | 50 | 100 | 41 | 0,82 | (51) | (52) |
| B21 | Passeriformes | Thraupidae | Thraupidae | family | 15,56 | 400 | 400 | 100 | 309 | 0,77 | (51) | (52) |
| B22 | Trogoniformes | Trogonidae | *Trogon-Priotelus* | genus | 24,66 | 23 | 23 | 100 | 20 | 0,87 | (51) | (52) |
| B23 | Piciformes | Picidae | Neotrop-*Picini* | tribe | 9,36 | 33 | 23 | 69,70 | 25 | 0,76 | (51) | (52) |
| B24 | Piciformes | Picidae | *Campephilus* (Neotrop-*Megapicini*) | genus | 6,01 | 11 | 11 | 100 | 10 | 0,91 | (51) | (52) |
| B25 | Piciformes | Picidae | *Melanerpes* | genus | 10,53 | 24 | 20 | 83,33 | 12 | 0,50 | (51) | (52) |
| B26 | Piciformes | Picidae | *Veniliornis* | genus | 5,38 | 14 | 14 | 100 | 12 | 0,86 | (51) | (52) |
| B27 | Piciformes | Picidae | *Picumnus* | genus | 12,17 | 28 | 28 | 100 | 8 | 0,29 | (51) | (52) |
| B28 | Piciformes | Ramphastides | Capitonidae, Semnornithidae,Ramphastidae | infraord | 20,11 | 59 | 59 | 100 | 44 | 0,75 | (51) | (52) |
| B29 | Galbuliformes | Bucconidae | Neotrop-Bucconidae | family | 42,09 | 37 | 37 | 100 | 10 | 0,27 | (51) | (52) |
| B30 | Accipitriformes | Accipitridae | *Buteogallus* | genus | 9,64 | 9 | 9 | 100 | 9 | 1,00 | (51) | (52) |
| B31 | Columbiformes | Columbidae | *Patagioenas* | genus | 11,05 | 17 | 17 | 100 | 12 | 0,71 | (51) | (52) |
| B32 | Columbiformes | Columbidae | Neotrop-Columbinae | tribe | 20,68 | 17 | 17 | 100 | 13 | 0,76 | (51) | (52) |
| S1 | Squamata | Colubridae (sf: Dipsadidae) | Colubridae (sf: Dipsadidae) | tribe | 34,35 | 154 | 154 | 100,00 | 76 | 0,49 | (56) | (57) |
| S2 | Squamata | Colubridae (sf: Colubrinae) | Colubridae (sf: Colubrinae) | tribe | 26,03 | 39 | 28 | 71,79 | 15 | 0,38 | (56) | (57) |
| S3 | Squamata | Elapidae | Elapidae | family | 21,78 | 80 | 80 | 100,00 | 19 | 0,24 | (56) | (57) |
| S4 | Squamata | Viperidae (sf. Crotalinae) | Viperidae (sf. Crotalinae) | tribe | 21,14 | 21 | 21 | 100,00 | 13 | 0,62 | (56) | (57) |
| S5 | Squamata | Viperidae (sf. Crotalinae) | Viperidae (sf. Crotalinae) | tribe | 21,41 | 71 | 71 | 100,00 | 43 | 0,61 | (56) | (57) |
| S6 | Squamata | Boidae | Boidae | family | 44,84 | 32 | 32 | 100,00 | 15 | 0,47 | (56) | (57) |
| S7 | Squamata | Aniliidae, Tropidophiidae | Aniliidae, Tropidophiidae | family | 80,54 | 35 | 35 | 100,00 | 9 | 0,26 | (56) | (57) |
| S8 | Squamata | Typhlopidae | Typhlopidae | family | 43,74 | 59 | 59 | 100,00 | 29 | 0,49 | (56) | (57) |
| S9 | Squamata | Corytophanidae, Dactyloidae | Corytophanidae, Dactyloidae | family | 83,4 | 436 | 436 | 100,00 | 214 | 0,49 | (56) | (57) |
| S10 | Squamata | Liolaemidae | Liolaemidae | family | 71,14 | 308 | 308 | 100,00 | 119 | 0,39 | (56) | (57) |
| S11 | Squamata | Leiosauridae | Leiosauridae | family | 42,19 | 33 | 33 | 100,00 | 11 | 0,33 | (56) | (57) |
| S12 | Squamata | Polychrotidae, Hoplocercidae | Polychrotidae, Hoplocercidae | family | 81,21 | 27 | 27 | 100,00 | 12 | 0,44 | (56) | (57) |
| S13 | Squamata | Phrynosomatidae | Phrynosomatidae | family | 19,79 | 102 | 50 | 49,02 | 44 | 0,43 | (56) | (57) |
| S14 | Squamata | Iguanidae | Iguanidae | family | 34,56 | 29 | 29 | 100,00 | 22 | 0,76 | (56) | (57) |
| S15 | Squamata | Tropiduridae | Tropiduridae | family | 88,47 | 136 | 136 | 100,00 | 78 | 0,57 | (56) | (57) |
| S16 | Squamata | Anguidae (Gerrhonotinae) | Anguidae (Gerrhonotinae) | tribe | 26,47 | 43 | 43 | 100,00 | 18 | 0,42 | (56) | (57) |
| S17 | Squamata | Amphisbaenidae | Amphisbaenidae | family | 48,84 | 95 | 95 | 100,00 | 37 | 0,39 | (56) | (57) |
| S18 | Squamata | Teiidae, Alopoglossidae, Gymnophthalmidae | Teiidae, Alopoglossidae, Gymnophthalmidae | family | 86,27 | 337 | 292 | 86,65 | 144 | 0,43 | (56) | (57) |
| S19 | Squamata | Scincidae (Mabuyinae) | Scincidae (Mabuyinae) | tribe | 25,22 | 60 | 60 | 100,00 | 20 | 0,33 | (56) | (57) |
| S20 | Squamata | Xantusiidae | Xantusiidae | family | 36,33 | 19 | 19 | 100,00 | 16 | 0,84 | (56) | (57) |
| S21 | Squamata | Phyllodactylidae | Phyllodactylidae | family | 66,91 | 65 | 65 | 100,00 | 20 | 0,31 | (56) | (57) |
| S22 | Squamata | Sphaerodactylidae | Sphaerodactylidae | family | 70,88 | 170 | 170 | 100,00 | 69 | 0,41 | (56) | (57) |
| S23 | Squamata | Colubridae (sf: Dipsadidae) | Colubridae (sf: Dipsadidae) | tribe | 34,42 | 123 | 123 | 100,00 | 60 | 0,49 | (56) | (57) |
| S24 | Squamata | Colubridae (sf: Dipsadidae) | Colubridae (sf: Dipsadidae) | tribe | 29,25 | 313 | 313 | 100,00 | 45 | 0,14 | (56) | (57) |
| A1 | Anura | Aromobatidae | Aromobatidae | family | 67,18 | 127 | 127 | 100 | 118 | 0,93 | (58) | (59) |
| A2 | Anura | Dendrobatidae | Dendrobatidae | family | 61,2 | 136 | 136 | 100 | 136 | 1,00 | (58) | (59) |
| A3 | Anura | Hemiphractidae | Hemiphractidae | family | 80,69 | 109 | 109 | 100 | 86 | 0,79 | (58) | (59) |
| A4 | Anura | Eleutherodactylidae | Eleutherodactylidae | family | 72,08 | 217 | 212 | 97,7 | 170 | 0,78 | (58) | (59) |
| A5 | Anura | Craugastoridae (sf: Craugastorinae, excluding Haddadus) | Craugastoridae (sf: Craugastorinae, excluding Haddadus) | family | 69,87 | 131 | 126 | 96,2 | 61 | 0,47 | (58) | (60) |
| A6 | Anura | Craugastoridae (Ceuthomantinae and (part of) Holoadeninae (other is paraphyletic)) | Craugastoridae (Ceuthomantinae and (part of) Holoadeninae (other is paraphyletic)) | family | 68,16 | 625 | 625 | 100 | 295 | 0,47 | (58) | (60) |
| A7 | Anura | Hylidae (Phyllomedusinae) | Hylidae (Phyllomedusinae) | tribe | 54,78 | 63 | 63 | 100 | 50 | 0,79 | (58) | (61) |
| A8 | Anura | Hylidae (Hylinae) | Hylidae (Hylinae) | tribe | 69,93 | 183 | 183 | 100 | 117 | 0,64 | (58) | (59) |
| A9 | Anura | Hylidae (Hylinae) | Hylidae (Hylinae) | tribe | 72,8 | 252 | 252 | 100 | 147 | 0,58 | (58) | (59) |
| A10 | Anura | Hylidae (Lophyohylinae) | Hylidae (Lophyohylinae) | tribe | 43,23 | 85 | 85 | 100 | 60 | 0,71 | (58) | (59) |
| A11 | Anura | Odontophrynidae, Ceratophryidae, Rhinodermatidae, Telmatobiidae, Cycloramphidae, Hylodidae, Batrichylidae, Alsodidae | Odontophrynidae, Ceratophryidae, Rhinodermatidae, Telmatobiidae, Cycloramphidae, Hylodidae, Batrichylidae, Alsodidae | family | 87,37 | 259 | 259 | 100 | 127 | 0,49 | (58) | (59) |
| A12 | Anura | Leptodactylidae | Leptodactylidae | family | 78,07 | 211 | 211 | 100 | 186 | 0,88 | (58) | (59) |
| A13 | Anura | Centrolenidae | Centrolenidae | family | 33,39 | 158 | 158 | 100 | 128 | 0,81 | (58) | (59) |
| A14 | Anura | Bufonidae | Bufonidae | family | 53,95 | 119 | 119 | 100 | 54 | 0,45 | (58) | (59) |
| A15 | Anura | Bufonidae | Bufonidae | family | 33,89 | 157 | 132 | 84 | 110 | 0,70 | (58) | (60, 61) |
| A16 | Caudata | Plethodontidae (sf: Hemidactyliinae) | Plethodontidae (sf: Hemidactyliinae) | family | 73,05 | 307 | 307 | 100 | 159 | 0,52 | (62) | (59) |

**Reference list**

1. A. E. Zanne, *et al.*, Three keys to the radiation of angiosperms into freezing environments. *Nature* **506**, 89–92 (2014).

2. , The Plant List (2010). Version 1. Published on Internet; http://www.theplantlist.org/ (accessed May 2019).

3. T. E. Särkinen, *et al.*, Recent oceanic long-distance dispersal and divergence in the amphi-Atlantic rain forest genus *Renealmia* L.f. (Zingiberaceae). *Mol. Phylogenet. Evol.* **44**, 968–980 (2007).

4. C. D. Specht, D. W. Stevenson, A new phylogeny-based generic classification of Costaceae (Zingiberales). *Taxon* **55**, 153–163 (2006).

5. M. D. Pirie, P. J. M. Maas, R. A. Wilschut, H. Melchers-Sharrott, L. W. Chatrou, Parallel diversifications of *Cremastosperma* and *Mosannona* (Annonaceae), tropical rainforest trees tracking Neogene upheaval of South America. *R. Soc. Open Sci.* **5**, 171561 (2018).

6. P. E. Berry, W. J. Hahn, K. J. Sytsma, J. C. Hall, A. Mast, Phylogenetic relationships and biogeography of *Fuchsia* (Onagraceae) based on noncoding nuclear and chloroplast DNA data. *Am. J. Bot.* **91**, 601–614 (2004).

7. G. Davidse, *Flora mesoamericana. 4: Parte 1. Cucurbitaceae a Polemoniaceae* (Univ. Nacional Autónoma de México, Inst. de Biología, 2009).

8. , Tropicos.org (2008).

9. M. F. Simon, *et al.*, Recent assembly of the Cerrado, a Neotropical plant diversity hotspot, by in situ evolution of adaptations to fire. *Proc. Natl. Acad. Sci.* **106**, 20359–20364 (2009).

10. G. Stride, S. Nylinder, U. Swenson, Revisiting the biogeography of *Sideroxylon* (Sapotaceae) and an evaluation of the taxonomic status of *Argania* and *Spiniluma*. *Aust. Syst. Bot.* **27**, 104–118 (2014).

11. Y.-Y. Huang, S. A. Mori, L. M. Kelly, Toward a phylogenetic-based generic classification of Neotropical Lecythidaceae-I. Status of *Bertholletia*, *Corythophora*, *Eschweilera* and *Lecythis*. *Phytotaxa* **203**, 85–121 (2015).

12. T. E. Särkinen, “Historical assembly of seasonally dry tropical forest diversity in the tropical Andes,” University of Oxford. (2010).

13. A. Antonelli, J. A. A. Nylander, C. Persson, I. Sanmartín, Tracing the impact of the Andean uplift on Neotropical plant evolution. *Proc. Natl. Acad. Sci. U. S. A.* **106**, 9749–9754 (2009).

14. M. Lavin, M. F. Wojciechowski, P. Gasson, C. Hughes, E. Wheeler, Phylogeny of robinioid legumes (Fabaceae) revisited: *Coursetia* and *Gliricidia* recircumscribed, and a biogeographical appraisal of the Caribbean endemics. *Syst. Bot.* **28**, 387–409 (2003).

15. R. H. J. Erkens, J. W. Maas, T. L. P. Couvreur, From Africa via Europe to South America: migrational route of a species-rich genus of Neotropical lowland rain forest trees (*Guatteria*, Annonaceae). *J. Biogeogr.* **36**, 2338–2352 (2009).

16. A. Antonelli, I. Sanmartín, Mass extinction, gradual cooling, or rapid radiation? Reconstructing the spatiotemporal evolution of the ancient angiosperm genus *Hedyosmum* (Chloranthaceae) using empirical and simulated approaches. *Syst. Biol.* **60**, 596–615 (2011).

17. L. P. Lagomarsino, F. L. Condamine, A. Antonelli, A. Mulch, C. C. Davis, The abiotic and biotic drivers of rapid diversification in Andean bellflowers (Campanulaceae). *New Phytol.* **210**, 1430–1442 (2016).

18. F. L. Condamine, N. S. Nagalingum, C. R. Marshall, H. Morlon, Origin and diversification of living cycads: A cautionary tale on the impact of the branching process prior in Bayesian molecular dating. *BMC Evol. Biol.* **15**, 65 (2015).

19. C. S. Drummond, R. J. Eastwood, S. T. S. Miotto, C. E. Hughes, Multiple continental radiations and correlates of diversification in *Lupinus* (Leguminosae): testing for key innovation with incomplete taxon sampling. *Syst. Biol.* **61**, 443–460 (2012).

20. M. C. M. P. de Medeiros, L. G. Lohmann, Phylogeny and biogeography of *Tynanthus* Miers (Bignonieae, Bignoniaceae). *Mol. Phylogenet. Evol.* **85**, 32–40 (2015).

21. S. Faurby, W. L. Eiserhardt, W. J. Baker, J.-C. Svenning, An all-evidence species-level supertree for the palms (Arecaceae). *Mol. Phylogenet. Evol.* **100**, 57–69 (2016).

22. M. F. Simon, *et al.*, The evolutionary history of *Mimosa* (Leguminosae): toward a phylogeny of the sensitive plants. *Am. J. Bot.* **98**, 1201–1221 (2011).

23. O. A. Pérez-Escobar, *et al.*, Recent origin and rapid speciation of Neotropical orchids in the world’s richest plant biodiversity hotspot. *New Phytol.* **215**, 891–905 (2017).

24. E. L. Spriggs, P.-A. Christin, E. J. Edwards, C4 photosynthesis promoted species diversification during the Miocene grassland expansion. *PLoS One* **9**, e97722 (2014).

25. K. G. Dexter, *et al.*, Dispersal assembly of rain forest tree communities across the Amazon basin. *Proc. Natl. Acad. Sci.* **114**, 2645–2650 (2017).

26. M. L. Serrano-Serrano, J. Rolland, J. L. Clark, N. Salamin, M. Perret, Hummingbird pollination and the diversification of angiosperms: an old and successful association in Gesneriaceae. *Proc. R. Soc. B Biol. Sci.* **284**, 20162816 (2017).

27. P. V. A. Fine, F. Zapata, D. C. Daly, Investigating processes of Neotropical rain forest tree diversification by examining the evolution and historical biogeography of the Protieae (Burseraceae). *Evolution* **68**, 1988–2004 (2014).

28. T. Särkinen, L. Bohs, R. G. Olmstead, S. Knapp, A phylogenetic framework for evolutionary study of the nightshades (Solanaceae): a dated 1000-tip tree. *BMC Evol. Biol.* **13**, 214 (2013).

29. D. L. De-Silva, *et al.*, North Andean origin and diversification of the largest ithomiine butterfly genus. *Sci. Rep.* **7**, 45966 (2017).

30. T. L. Weese, L. Bohs, A three-gene phylogeny of the genus *Solanum* (Solanaceae). *Syst. Bot.* **32**, 445–463 (2007).

31. R. J. Schley, *et al.*, Is Amazonia a ‘museum’for Neotropical trees? The evolution of the *Brownea* clade (Detarioideae, Leguminosae). *Mol. Phylogenet. Evol.* **126**, 279–292 (2018).

32. A. C. Martins, M. D. Scherz, S. S. Renner, Several origins of floral oil in the Angelonieae, a southern hemisphere disjunct clade of Plantaginaceae. *Am. J. Bot.* **101**, 2113–2120 (2014).

33. M. F. Santos, *et al.*, Biogeographical patterns of *Myrcia* sl (Myrtaceae) and their correlation with geological and climatic history in the Neotropics. *Mol. Phylogenet. Evol.* **108**, 34–48 (2017).

34. R. Govaerts, M. Sobral, P. Ashton, F. Barrie, World checklist of Myrtaceae. Royal Botanic Gardens (2016).

35. R. S. Couto, *et al.*, Time calibrated tree of *Dioscorea* (Dioscoreaceae) indicates four origins of yams in the Neotropics since the Eocene. *Bot. J. Linn. Soc.* **188**, 144–160 (2018).

36. R. Arévalo, B. W. van Ee, R. Riina, P. E. Berry, A. C. Wiedenhoeft, Force of habit: shrubs, trees and contingent evolution of wood anatomical diversity using *Croton* (Euphorbiaceae) as a model system. *Ann. Bot.* **119**, 563–579 (2017).

37. H.-J. Esser, P. E. Berry, R. Riina, EuphORBia: a global inventory of the spurges. *Blumea-Biodiversity, Evol. Biogeogr. Plants* **54**, 11–12 (2009).

38. Y. Wang, *et al.*, Phylogeny and infrageneric classification of *Symplocos* (Symplocaceae) inferred from DNA sequence data. *Am. J. Bot.* **91**, 1901–1914 (2004).

39. G. C. Gibb, *et al.*, Shotgun mitogenomics provides a reference phylogenetic framework and timescale for living xenarthrans. *Mol. Biol. Evol.* **33**, 621–642 (2016).

40. D. E. Wilson, D. M. Reeder, *Mammal species of the world: a taxonomic and geographic reference* (JHU Press, 2005).

41. D. Rojas, O. M. Warsi, L. M. Davalos, Bats (Chiroptera: Noctilionoidea) challenge a recent origin of extant neotropical diversity. *Syst. Biol.* **65**, 432–448 (2016).

42. O. R. P. Bininda-Emonds, *et al.*, The delayed rise of present-day mammals. *Nature* **446**, 507–512 (2007).

43. M. S. Springer, *et al.*, Macroevolutionary dynamics and historical biogeography of primate diversification inferred from a species supermatrix. *PLoS One* **7**, e49521 (2012).

44. A. B. Rylands, R. A. Mittermeier, “The Diversity of the New World primates (Platyrrhini): an annotated taxonomy” in *South American Primates: Comparative Perspectives in the Study of Behavior, Ecology, and Conservation*, P. A. Garber, A. Estrada, J. C. Bicca-Marques, E. W. Heymann, K. B. Strier, Eds. (Springer New York, 2009), pp. 23–54.

45. S. A. Jansa, F. K. Barker, R. S. Voss, The early diversification history of didelphid marsupials: A window into South America’s “splendid isolation.” *Evolution* **68**, 684–695 (2014).

46. R. Maestri, *et al.*, The ecology of a continental evolutionary radiation: Is the radiation of sigmodontine rodents adaptive? *Evolution* **71**, 610–632 (2017).

47. B. K. Lim, Divergence times and origin of neotropical sheath-tailed bats (tribe Diclidurini) in South America. *Mol. Phylogenet. Evol.* **45**, 777–791 (2007).

48. O. Toljagić, K. L. Voje, M. Matschiner, L. H. Liow, T. F. Hansen, Millions of years behind: slow adaptation of ruminants to grasslands. *Syst. Biol.* **67**, 145–157 (2017).

49. K. Nyakatura, O. R. P. Bininda-Emonds, Updating the evolutionary history of Carnivora (Mammalia): a new species-level supertree complete with divergence time estimates. *BMC Biol.* **10**, 12 (2012).

50. N. S. Upham, B. D. Patterson, Diversification and biogeography of the Neotropical caviomorph lineage Octodontoidea (Rodentia: Hystricognathi). *Mol. Phylogenet. Evol.* **63**, 417–429 (2012).

51. W. Jetz, G. H. Thomas, J. B. Joy, K. Hartmann, A. O. Mooers, The global diversity of birds in space and time. *Nature* **491**, 444–448 (2012).

52. J. V Remsen Jr, HBW and BirdLife International Illustrated Checklist of the Birds of the World Volume 1: Non-passerines (2015).

53. J. A. McGuire, *et al.*, Molecular phylogenetics and the diversification of hummingbirds. *Curr. Biol.* **24**, 910–916 (2014).

54. E. P. Derryberry, *et al.*, Lineage diversification and morphological evolution in a large-scale continental radiation: The Neotropical ovenbirds and woodcreepers (aves: furnariidae). *Evolution* **65**, 2973–2986 (2011).

55. D. L. Slager, C. J. Battey, R. W. Bryson Jr, G. Voelker, J. Klicka, A multilocus phylogeny of a major New World avian radiation: the Vireonidae. *Mol. Phylogenet. Evol.* **80**, 95–104 (2014).

56. R. A. Pyron, F. T. Burbrink, Early origin of viviparity and multiple reversions to oviparity in squamate reptiles. *Ecol. Lett.* **17**, 13–21 (2014).

57. P. Uetz, P. Freed, J. Hošek, The Reptile Database, http://www.reptile-database.org, accessed 2018.

58. C. R. Hutter, S. M. Lambert, J. J. Wiens, Rapid diversification and time explain Amphibian richness at different scales in the tropical Andes, Earth’s most biodiverse Hotspot. *Am. Nat.* **190**, 828–843 (2017).

59. , AmphibiaWeb. 2019. https://amphibiaweb.org University of California, Berkeley, USA. Accessed 2018.

60. D. R. Frost, Amphibian Species of the World: an online reference. http://research.amnh.org/herpetology/amphibia/index.html. Accessed 2018. *Am. Museum Nat. Hist. New York, USA* (2019).

61. S. N. Stuart, *Threatened amphibians of the world* (Lynx Edicions, 2008).

62. R. A. Pyron, Biogeographic analysis reveals ancient continental vicariance and recent oceanic dispersal in amphibians. *Syst. Biol.* **63**, 779–797 (2014).
