# Supplementary material for: Diversification dynamics in the Neotropics through time, clades, and biogeographic regions
Source: eLife. 2022 Oct 27;11:e74503. doi: 10.7554/eLife.74503 (PMC9668338; doi:10.7554/eLife.74503)
Supplement: Figure 3—source data 1. [file elife-74503-fig3-data1.docx]

**Figure 3, Source Data 1.**

Source data for Figure 3a, and Figure 4a. Number of phylogenies and species supporting different diversification models. (among time-constant and time-variable), speciation trends (among speciation [lambda] constant, decreasing, increasing) and species richness dynamics (gradual increase [Sc. 1], exponential increase [Sc. 2], saturated increase [Sc. 3] and decline [Sc. 4]), based on traditional diversification rates in this study and only considering models where diversification rates are allowed to vary as a function of time.

|  |  | All | Plants | Mammals | Birds | Squamata | Amphibia |
| --- | --- | --- | --- | --- | --- | --- | --- |
|  | Total # of clades | 150 | 66 | 12 | 32 | 24 | 16 |
|  | Total # of species | 12512 | 6222 | 922 | 2216 | 1148 | 2004 |
| Diversification trend | # clades constant | 101 | 47 | 8 | 22 | 14 | 10 |
|  | # clades time-variable | 49 | 19 | 4 | 10 | 10 | 6 |
|  | # species constant | 5891 | 2861 | 404 | 1037 | 484 | 1105 |
|  | # species time-variable | 6621 | 3361 | 518 | 1179 | 664 | 899 |
| Speciation trend | # lambda decreasing | 28 | 3 | 3 | 10 | 7 | 5 |
|  | # lambda increasing | 12 | 7 | 1 | 0 | 3 | 1 |
|  | # lambda constant (mu varies) | 9 | 9 | 0 | 0 | 0 | 0 |
| Species richness dynamic | Gradual increase | 101 | 47 | 8 | 22 | 14 | 10 |
|  | Exponential increase | 20 | 17 | 1 | 0 | 1 | 1 |
|  | Saturated increase | 24 | 1 | 2 | 10 | 7 | 4 |
|  | Declining & Waning | 5 | 1 | 1 | 0 | 2 | 1 |
