# Supplementary material for: Diversification dynamics in the Neotropics through time, clades, and biogeographic regions
Source: eLife. 2022 Oct 27;11:e74503. doi: 10.7554/eLife.74503 (PMC9668338; doi:10.7554/eLife.74503)
Supplement: Figure 3—source data 2. [file elife-74503-fig3-data2.docx]

**Figure 3, Source Data 2.**

Source data for subfigure (b). Number of phylogenies and species supporting different diversification (among time-constant and time-variable models) and speciation trends (among decreasing, increasing) based on pulled diversification rates.

|  |  | All | Plants | Mammals | Birds | Squamata | Amphibia |
| --- | --- | --- | --- | --- | --- | --- | --- |
|  | Total # of clades | 150 | 66 | 12 | 32 | 24 | 16 |
|  | Total # of species | 12512 | 6222 | 922 | 2216 | 1148 | 2004 |
| Diversification trend | # clades constant | 95 | 39 | 6 | 24 | 18 | 8 |
|  | # clades time-variable | 55 | 27 | 6 | 8 | 6 | 8 |
|  | # species constant | 4913 | 1908 | 117 | 1270 | 684 | 934 |
|  | # species time-variable | 7599 | 4314 | 805 | 946 | 464 | 1070 |
| Speciation trend | # lambda decreasing | 51 | 25 | 6 | 8 | 5 | 7 |
|  | # lambda increasing | 4 | 2 | 0 | 0 | 1 | 1 |
