# Supplementary material for: Diversification dynamics in the Neotropics through time, clades, and biogeographic regions
Source: eLife. 2022 Oct 27;11:e74503. doi: 10.7554/eLife.74503 (PMC9668338; doi:10.7554/eLife.74503)
Supplement: Figure 4—source data 1. — Source data to generate Figure 4a is provided as Figure 3—source data 2. [file elife-74503-fig4-data1.docx]

**Figure 4, Source Data 1.**

Source data for subfigure b and c (Source data to generate subfigure 4a is provided as Figure 3 - source data file 2). Number of phylogenies and species supporting different diversification models (among time-constant and time-variable), and species richness dynamics (gradual increase [Sc. 1], exponential increase [Sc. 2], saturated increase [Sc. 3] and decline [Sc. 4]), based on traditional diversification rates and considering models where diversification rates are allowed to vary as a function of time, temperature and Andean uplift changes.

|  |  | All | Plants | Mammals | Birds | Squamata | Amphibia |
| --- | --- | --- | --- | --- | --- | --- | --- |
|  | Total # of clades | 150 | 66 | 12 | 32 | 24 | 16 |
|  | Total # of species | 12512 | 6222 | 922 | 2216 | 1148 | 2004 |
| Diversification trend | # clades constant | 76 | 39 | 6 | 19 | 7 | 5 |
|  | # clades time-variable | 74 | 27 | 6 | 13 | 17 | 11 |
|  | # species constant | 2989 | 1785 | 117 | 457 | 116 | 514 |
|  | # species time-variable | 9523 | 4437 | 805 | 1759 | 1032 | 1490 |
| Species richness dynamic | Gradual increase | 76 | 39 | 6 | 19 | 7 | 5 |
|  | Exponential increase | 30 | 19 | 1 | 0 | 4 | 6 |
|  | Saturated increase | 31 | 3 | 3 | 11 | 11 | 3 |
|  | Declining & Waning | 13 | 5 | 2 | 2 | 2 | 2 |
