# Supplementary material for: Diversification dynamics in the Neotropics through time, clades, and biogeographic regions
Source: eLife. 2022 Oct 27;11:e74503. doi: 10.7554/eLife.74503 (PMC9668338; doi:10.7554/eLife.74503)
Supplement: Figure 5—source data 1. [file elife-74503-fig5-data1.docx]

**Figure 5, Source Data 1.**

Number of phylogenies and species supporting different diversification models (among time-constant and time-variable), and diversification drivers (among time, temperature, Andean uplift) in this study.

|  |  | All | Plants | Mammals | Birds | Squamata | Amphibia |
| --- | --- | --- | --- | --- | --- | --- | --- |
|  | Total # of clades | 150 | 66 | 12 | 32 | 24 | 16 |
|  | Total # of species | 12512 | 6222 | 922 | 2216 | 1148 | 2004 |
| Diversification trend | # clades constant | 76 | 39 | 6 | 19 | 7 | 5 |
|  | # clades time-variable | 74 | 27 | 6 | 13 | 17 | 11 |
|  | # species constant | 2989 | 1785 | 117 | 457 | 116 | 514 |
|  | # species time-variable | 9523 | 4437 | 805 | 1759 | 1032 | 1490 |
| Environmental driver | Time-dependent models | 17 | 11 | 2 | 1 | 3 | 0 |
|  | Temperature-dependent models | 40 | 15 | 4 | 12 | 6 | 3 |
|  | Uplift-dependent models | 17 | 1 | 0 | 0 | 8 | 8 |
