# Supplementary material for: Diversification dynamics in the Neotropics through time, clades, and biogeographic regions
Source: eLife. 2022 Oct 27;11:e74503. doi: 10.7554/eLife.74503 (PMC9668338; doi:10.7554/eLife.74503)
Supplement: Figure 5—source data 2. [file elife-74503-fig5-data2.docx]

**Figure-5-source-data-2**. Diversification results for each clade showing whether diversification rates are best explained by a model with constant, time-, temperature-, or uplift-dependent diversification based on (1) the most supported model (lowest AIC value), and (2) the second most supported model. The dependency parameter of speciation [alpha] is provided for each tree and model. For each model category, we fitted three models in which speciation (B) and/or extinction (D) remain constant (CST), or vary continuously with time, with temperature changes, or with the elevation of the Andes.

| **Clade** | **Best model summary** | **Best Model** | **Alpha** | **Second best model summary** | **Second Best Model** | **Alpha** |
| --- | --- | --- | --- | --- | --- | --- |
| P1 | time | BCSTDTimeVar | NA | temperature | BCSTDTemp.Var | NA |
| P2 | time | BCSTDTimeVar | NA | temperature | BCSTDTemp.Var | NA |
| P3 | constant | BCSTDCST | NA | temperature | BCSTDTemp.Var | NA |
| P4 | constant | BCSTDCST | NA | temperature | BCSTDTemp.Var | NA |
| P5 | constant | BCSTDCST | NA | time | BCSTDTimeVar | NA |
| P6 | constant | BCSTDCST | NA | time | BTimeVarDCST | 0,017 |
| P7 | constant | BCSTDCST | NA | uplift | BAnde.VarDCST | 0,000 |
| P8 | temperature | BCSTDTemp.Var | NA | uplift | BAnde.VarDCST | 0,003 |
| P9 | constant | BCSTDCST | NA | temperature | BTemp.VarDCST | 0,027 |
| P10 | constant | BCSTDCST | NA | temperature | BTemp.VarDCST | 0,024 |
| P11 | constant | BCSTDCST | NA | temperature | BTemp.VarDCST | -0,145 |
| P12 | constant | BCSTDCST | NA | time | BCSTDTimeVar | NA |
| P13 | temperature | BTemp.VarDCST | 0,056 | time | BTimeVarDCST | 0,011 |
| P14 | constant | BCSTDCST | NA | time | BCSTDTimeVar | NA |
| P15 | constant | BCSTDCST | NA | time | BCSTDTimeVar | NA |
| P16 | constant | BCSTDCST | NA | temperature | BCSTDTemp.Var | NA |
| P17 | constant | BCSTDCST | NA | temperature | BTemp.VarDCST | 0,092 |
| P18 | constant | BCSTDCST | NA | temperature | BTemp.VarDCST | 0,564 |
| P19 | time | BCSTDTimeVar | NA | constant | BCSTDCST | NA |
| P20 | constant | BCSTDCST | NA | temperature | BTemp.VarDCST | 0,028 |
| P21 | temperature | BTemp.VarDTemp.Var | 0,066 | temperature | BTemp.VarDCST | 0,293 |
| P22 | constant | BCSTDCST | NA | temperature | BTemp.VarDCST | 0,102 |
| P23 | constant | BCSTDCST | NA | time | BCSTDTimeVar | NA |
| P24 | time | BTimeVarDCST | -0,292 | temperature | BTemp.VarDCST | -0,163 |
| P25 | constant | BCSTDCST | NA | temperature | BTemp.VarDCST | 0,100 |
| P26 | constant | BCSTDCST | NA | time | BTimeVarDCST | 0,042 |
| P27 | constant | BCSTDCST | NA | time | BCSTDTimeVar | NA |
| P28 | time | BCSTDTimeVar | NA | temperature | BCSTDTemp.Var | NA |
| P29 | temperature | BTemp.VarDCST | 0,325 | constant | BCSTDCST | NA |
| P30 | constant | BCSTDCST | NA | time | BCSTDTimeVar | NA |
| P31 | constant | BCSTDCST | NA | time | BTimeVarDCST | -0,028 |
| P32 | constant | BCSTDCST | NA | temperature | BTemp.VarDCST | 0,087 |
| P33 | constant | BCSTDCST | NA | time | BTimeVarDCST | 0,076 |
| P34 | constant | BCSTDCST | NA | time | BCSTDTimeVar | NA |
| P35 | temperature | BTemp.VarDCST | 0,071 | constant | BCSTDCST | NA |
| P36 | temperature | BCSTDTemp.Var | NA | constant | BCSTDCST | NA |
| P37 | temperature | BTemp.VarDTemp.Var | 0,168 | temperature | BCSTDTemp.Var | NA |
| P38 | time | BCSTDTimeVar | NA | temperature | BCSTDTemp.Var | NA |
| P39 | constant | BCSTDCST | NA | time | BCSTDTimeVar | NA |
| P40 | temperature | BTemp.VarDCST | -0,194 | time | BTimeVarDCST | -0,051 |
| P41 | constant | BCSTDCST | NA | time | BTimeVarDCST | 0,000 |
| P42 | temperature | BTemp.VarDCST | -0,192 | temperature | BTemp.VarDTemp.Var | -0,192 |
| P43 | time | BCSTDTimeVar | NA | time | BTimeVarDCST | -0,047 |
| P44 | time | BTimeVarDCST | -0,678 | temperature | BTemp.VarDCST | -0,801 |
| P45 | uplift | BCSTDAnde.Var | NA | uplift | BAnde.VarDAnde.Var | 0,000 |
| P46 | temperature | BTemp.VarDCST | 0,106 | temperature | BTemp.VarDTemp.Var | 0,106 |
| P47 | constant | BCSTDCST | NA | temperature | BTemp.VarDCST | -0,227 |
| P48 | constant | BCSTDCST | NA | temperature | BCSTDTemp.Var | NA |
| P49 | constant | BCSTDCST | NA | temperature | BTemp.VarDCST | -0,325 |
| P50 | constant | BCSTDCST | NA | time | BCSTDTimeVar | NA |
| P51 | temperature | BTemp.VarDCST | -0,165 | temperature | BCSTDTemp.Var | NA |
| P52 | time | BTimeVarDCST | -0,074 | uplift | BAnde.VarDCST | 0,001 |
| P53 | constant | BCSTDCST | NA | time | BCSTDTimeVar | NA |
| P54 | temperature | BCSTDTemp.Var | NA | constant | BCSTDCST | NA |
| P55 | temperature | BTemp.VarDCST | -0,381 | temperature | BTemp.VarDTemp.Var | -0,381 |
| P56 | temperature | BTemp.VarDTemp.Var | 0,469 | constant | BCSTDCST | NA |
| P57 | constant | BCSTDCST | NA | temperature | BCSTDTemp.Var | NA |
| P58 | temperature | BTemp.VarDCST | -0,127 | temperature | BCSTDTemp.Var | NA |
| P59 | time | BCSTDTimeVar | NA | temperature | BCSTDTemp.Var | NA |
| P60 | time | BCSTDTimeVar | NA | uplift | BCSTDAnde.Var | NA |
| P61 | constant | BCSTDCST | NA | temperature | BCSTDTemp.Var | NA |
| P62 | constant | BCSTDCST | NA | time | BCSTDTimeVar | NA |
| P63 | constant | BCSTDCST | NA | time | BTimeVarDCST | 253504 |
| P64 | constant | BCSTDCST | NA | time | BTimeVarDCST | 0,281 |
| P65 | constant | BCSTDCST | NA | temperature | BTemp.VarDCST | 0,018 |
| P66 | constant | BCSTDCST | NA | time | BTimeVarDCST | 0,111 |
| M1 | constant | BCSTDCST | NA | temperature | BTemp.VarDCST | -0,094 |
| M2 | temperature | BTemp.VarDTemp.Var | 0,304 | constant | BCSTDCST | NA |
| M3 | time | BTimeVarDTimeVar | 0,364 | temperature | BTemp.VarDCST | 0,237 |
| M4 | temperature | BTemp.VarDCST | -0,341 | temperature | BTemp.VarDTemp.Var | -0,341 |
| M5 | constant | BCSTDCST | NA | time | BTimeVarDCST | -0,033 |
| M6 | temperature | BTemp.VarDCST | 0,099 | temperature | BTemp.VarDTemp.Var | 0,099 |
| M7 | constant | BCSTDCST | NA | temperature | BTemp.VarDCST | 0,295 |
| M8 | constant | BCSTDCST | NA | time | BTimeVarDCST | 0,022 |
| M9 | time | BTimeVarDCST | 0,059 | uplift | BAnde.VarDCST | 0,000 |
| M10 | constant | BCSTDCST | NA | temperature | BTemp.VarDCST | 0,361 |
| M11 | constant | BCSTDCST | NA | temperature | BTemp.VarDCST | 0,069 |
| M12 | temperature | BTemp.VarDTemp.Var | 0,355 | time | BTimeVarDCST | -0,038 |
| B1 | constant | BCSTDCST | NA | temperature | BCSTDTemp.Var | NA |
| B2 | temperature | BTemp.VarDCST | 0,065 | constant | BCSTDCST | NA |
| B3 | constant | BCSTDCST | NA | temperature | BCSTDTemp.Var | NA |
| B4 | constant | BCSTDCST | NA | temperature | BCSTDTemp.Var | NA |
| B5 | temperature | BTemp.VarDCST | 0,120 | constant | BCSTDCST | NA |
| B6 | constant | BCSTDCST | NA | uplift | BCSTDAnde.Var | NA |
| B7 | constant | BCSTDCST | NA | time | BCSTDTimeVar | NA |
| B8 | temperature | BTemp.VarDCST | 0,253 | temperature | BTemp.VarDTemp.Var | 0,253 |
| B9 | temperature | BTemp.VarDCST | 0,068 | temperature | BTemp.VarDTemp.Var | 0,068 |
| B10 | temperature | BTemp.VarDCST | 0,065 | time | BTimeVarDCST | 0,023 |
| B11 | constant | BCSTDCST | NA | temperature | BTemp.VarDCST | 0,042 |
| B12 | constant | BCSTDCST | NA | time | BCSTDTimeVar | NA |
| B13 | temperature | BTemp.VarDCST | 0,199 | temperature | BTemp.VarDTemp.Var | 0,199 |
| B14 | time | BTimeVarDCST | 0,367 | uplift | BAnde.VarDCST | -0,003 |
| B15 | constant | BCSTDCST | NA | temperature | BTemp.VarDCST | 0,267 |
| B16 | temperature | BTemp.VarDCST | 0,215 | temperature | BTemp.VarDTemp.Var | 0,215 |
| B17 | constant | BCSTDCST | NA | temperature | BTemp.VarDCST | 0,201 |
| B18 | temperature | BTemp.VarDCST | 0,411 | time | BTimeVarDCST | 0,195 |
| B19 | temperature | BTemp.VarDCST | 0,267 | temperature | BTemp.VarDTemp.Var | 0,265 |
| B20 | temperature | BTemp.VarDCST | 0,337 | time | BTimeVarDCST | 0,129 |
| B21 | temperature | BTemp.VarDCST | 0,297 | time | BTimeVarDCST | 0,119 |
| B22 | constant | BCSTDCST | NA | temperature | BTemp.VarDCST | 0,072 |
| B23 | temperature | BTemp.VarDCST | 0,376 | time | BTimeVarDCST | 0,178 |
| B24 | constant | BCSTDCST | NA | temperature | BTemp.VarDCST | 0,313 |
| B25 | constant | BCSTDCST | NA | temperature | BTemp.VarDCST | 0,457 |
| B26 | constant | BCSTDCST | NA | temperature | BTemp.VarDCST | 0,329 |
| B27 | constant | BCSTDCST | NA | temperature | BCSTDTemp.Var | NA |
| B28 | constant | BCSTDCST | NA | temperature | BTemp.VarDTemp.Var | 0,593 |
| B29 | constant | BCSTDCST | NA | temperature | BCSTDTemp.Var | NA |
| B30 | constant | BCSTDCST | NA | temperature | BTemp.VarDCST | 0,237 |
| B31 | constant | BCSTDCST | NA | time | BCSTDTimeVar | NA |
| B32 | constant | BCSTDCST | NA | time | BTimeVarDCST | 0,025 |
| S1 | temperature | BTemp.VarDCST | 0,157 | time | BTimeVarDCST | 0,035 |
| S2 | time | BTimeVarDCST | 0,066 | constant | BCSTDCST | NA |
| S3 | constant | BCSTDCST | NA | time | BCSTDTimeVar | NA |
| S4 | constant | BCSTDCST | NA | temperature | BTemp.VarDCST | 0,164 |
| S5 | temperature | BTemp.VarDCST | 0,158 | constant | BCSTDCST | NA |
| S6 | constant | BCSTDCST | NA | uplift | BAnde.VarDCST | 0,000 |
| S7 | constant | BCSTDCST | NA | time | BCSTDTimeVar | NA |
| S8 | time | BTimeVarDTimeVar | -0,059 | temperature | BCSTDTemp.Var | NA |
| S9 | uplift | BAnde.VarDCST | 0,000 | uplift | BAnde.VarDAnde.Var | 0,000 |
| S10 | uplift | BAnde.VarDAnde.Var | 0,000 | time | BTimeVarDCST | -0,039 |
| S11 | uplift | BAnde.VarDCST | -0,001 | constant | BCSTDCST | NA |
| S12 | uplift | BAnde.VarDAnde.Var | -0,001 | uplift | BCSTDAnde.Var | NA |
| S13 | time | BTimeVarDCST | 0,049 | uplift | BAnde.VarDCST | 0,000 |
| S14 | constant | BCSTDCST | NA | time | BCSTDTimeVar | NA |
| S15 | uplift | BAnde.VarDAnde.Var | 0,000 | uplift | BAnde.VarDCST | 0,000 |
| S16 | constant | BCSTDCST | NA | uplift | BAnde.VarDCST | 0,000 |
| S17 | temperature | BTemp.VarDCST | 0,224 | time | BTimeVarDCST | 0,043 |
| S18 | uplift | BAnde.VarDAnde.Var | 0,000 | constant | BCSTDCST | NA |
| S19 | temperature | BTemp.VarDCST | 0,293 | temperature | BTemp.VarDTemp.Var | 0,658 |
| S20 | uplift | BAnde.VarDCST | 0,000 | temperature | BTemp.VarDCST | 0,313 |
| S21 | constant | BCSTDCST | NA | uplift | BCSTDAnde.Var | NA |
| S22 | uplift | BAnde.VarDCST | 0,000 | uplift | BAnde.VarDAnde.Var | 0,000 |
| S23 | temperature | BTemp.VarDCST | 0,124 | constant | BCSTDCST | NA |
| S24 | temperature | BTemp.VarDCST | 0,150 | time | BTimeVarDCST | 0,029 |
| A1 | uplift | BAnde.VarDAnde.Var | 0,000 | uplift | BAnde.VarDCST | 0,000 |
| A2 | temperature | BTemp.VarDCST | 0,062 | temperature | BTemp.VarDTemp.Var | 0,062 |
| A3 | constant | BCSTDCST | NA | uplift | BAnde.VarDCST | 0,000 |
| A4 | uplift | BAnde.VarDAnde.Var | -0,001 | time | BTimeVarDTimeVar | 0,049 |
| A5 | uplift | BAnde.VarDAnde.Var | -0,001 | uplift | BAnde.VarDCST | 0,000 |
| A6 | uplift | BAnde.VarDAnde.Var | 0,000 | uplift | BAnde.VarDCST | 0,000 |
| A7 | uplift | BAnde.VarDCST | 0,000 | constant | BCSTDCST | NA |
| A8 | constant | BCSTDCST | NA | uplift | BCSTDAnde.Var | NA |
| A9 | constant | BCSTDCST | NA | uplift | BAnde.VarDAnde.Var | 0,000 |
| A10 | temperature | BTemp.VarDCST | 0,158 | temperature | BTemp.VarDTemp.Var | 0,300 |
| A11 | uplift | BCSTDAnde.Var | NA | time | BTimeVarDTimeVar | -0,018 |
| A12 | uplift | BAnde.VarDAnde.Var | 0,000 | uplift | BAnde.VarDCST | NA |
| A13 | temperature | BTemp.VarDCST | 0,200 | temperature | BTemp.VarDTemp.Var | 0,199 |
| A14 | constant | BCSTDCST | NA | temperature | BTemp.VarDTemp.Var | 0,241 |
| A15 | constant | BCSTDCST | NA | time | BTimeVarDCST | 0,014 |
| A16 | uplift | BAnde.VarDAnde.Var | 0,000 | constant | BCSTDCST | NA |
