# Supplementary material for: Diversification dynamics in the Neotropics through time, clades, and biogeographic regions
Source: eLife. 2022 Oct 27;11:e74503. doi: 10.7554/eLife.74503 (PMC9668338; doi:10.7554/eLife.74503)
Supplement: Figure 6—source data 1. [file elife-74503-fig6-data1.docx]

**Figure 6 - Source data 1.**

Species-richness per clade and area. For each of the 150 phylogenetic clades considered in this study, we provide the number of species occurring in each of the 13 Neotropical ecoregions of the WWF biome classification. Clade numbers correspond with Figure 2, Source data 1. Abbreviations: Amazonia = Amaz., Atlantic Forest = Atl.For., Bahama-Antilles = Baham., Caatinga, Central Andes = C.Andes, Cerrado, Chaco, Chocó, Guiana Shield, Mesoamerica = Mesoam., and the Northern Andes = N.Andes, temperate South America = TemSAm., and Elsewhere = Elsewh.

| Clade | Amaz. | Atl.For. | Baham. | Caatinga | C.Andes | Cerrado | Chaco | Chocó | Elsewh. | Galap. | Guiana | Mesoam. | N.Andes | TemSAm |
| --- | --- | --- | --- | --- | --- | --- | --- | --- | --- | --- | --- | --- | --- | --- |
| A1 | 46 | 1 | 1 | 0 | 3 | 0 | 0 | 3 | 0 | 0 | 48 | 1 | 26 | 0 |
| A2 | 60 | 1 | 0 | 0 | 12 | 4 | 0 | 26 | 0 | 0 | 8 | 17 | 59 | 0 |
| A3 | 5 | 13 | 0 | 1 | 25 | 1 | 1 | 6 | 0 | 0 | 18 | 2 | 31 | 1 |
| A4 | 2 | 5 | 0 | 2 | 0 | 0 | 0 | 1 | 0 | 0 | 2 | 7 | 0 | 0 |
| A5 | 4 | 0 | 143 | 0 | 0 | 0 | 0 | 5 | 2 | 0 | 1 | 63 | 3 | 0 |
| A6 | 43 | 4 | 2 | 1 | 77 | 4 | 1 | 26 | 0 | 0 | 25 | 28 | 155 | 1 |
| A7 | 12 | 15 | 0 | 4 | 3 | 9 | 4 | 3 | 0 | 0 | 6 | 11 | 6 | 2 |
| A8 | 20 | 44 | 1 | 4 | 12 | 10 | 5 | 8 | 0 | 0 | 28 | 4 | 28 | 4 |
| A9 | 64 | 41 | 0 | 5 | 3 | 32 | 11 | 11 | 0 | 0 | 28 | 9 | 31 | 5 |
| A10 | 26 | 9 | 8 | 4 | 5 | 5 | 2 | 3 | 1 | 0 | 21 | 2 | 8 | 2 |
| A11 | 2 | 37 | 0 | 4 | 33 | 4 | 8 | 0 | 0 | 0 | 1 | 0 | 2 | 46 |
| A12 | 53 | 64 | 3 | 14 | 12 | 49 | 22 | 18 | 0 | 0 | 38 | 11 | 22 | 17 |
| A13 | 23 | 2 | 0 | 0 | 19 | 0 | 0 | 22 | 0 | 0 | 18 | 15 | 59 | 0 |
| A14 | 6 | 1 | 0 | 0 | 7 | 0 | 0 | 1 | 0 | 0 | 12 | 5 | 27 | 0 |
| A15 | 19 | 14 | 0 | 5 | 13 | 13 | 5 | 5 | 25 | 0 | 10 | 30 | 8 | 6 |
| A16 | 4 | 0 | 0 | 0 | 1 | 0 | 0 | 2 | 8 | 0 | 0 | 149 | 6 | 0 |
| B1 | 15 | 5 | 0 | 1 | 8 | 7 | 5 | 8 | 3 | 0 | 10 | 9 | 11 | 2 |
| B2 | 61 | 29 | 6 | 20 | 90 | 24 | 12 | 59 | 19 | 0 | 53 | 70 | 127 | 16 |
| B3 | 4 | 3 | 0 | 2 | 4 | 4 | 4 | 2 | 0 | 0 | 3 | 2 | 3 | 5 |
| B4 | 47 | 24 | 11 | 19 | 32 | 33 | 16 | 27 | 16 | 0 | 36 | 19 | 31 | 13 |
| B5 | 11 | 8 | 0 | 1 | 19 | 5 | 3 | 4 | 0 | 0 | 6 | 4 | 25 | 11 |
| B6 | 3 | 2 | 0 | 2 | 3 | 2 | 1 | 2 | 0 | 0 | 0 | 1 | 2 | 2 |
| B7 | 5 | 3 | 0 | 2 | 3 | 0 | 1 | 2 | 0 | 0 | 3 | 2 | 4 | 1 |
| B8 | 112 | 32 | 0 | 20 | 63 | 34 | 9 | 35 | 0 | 0 | 55 | 26 | 68 | 4 |
| B9 | 86 | 53 | 0 | 25 | 140 | 58 | 52 | 45 | 3 | 0 | 66 | 42 | 96 | 76 |
| B10 | 153 | 104 | 1 | 72 | 169 | 107 | 76 | 101 | 37 | 0 | 131 | 98 | 148 | 72 |
| B11 | 46 | 16 | 0 | 7 | 31 | 18 | 1 | 16 | 0 | 0 | 33 | 13 | 28 | 3 |
| B12 | 4 | 0 | 1 | 0 | 4 | 1 | 0 | 3 | 13 | 0 | 4 | 16 | 7 | 0 |
| B13 | 8 | 5 | 0 | 1 | 5 | 4 | 3 | 2 | 12 | 0 | 5 | 18 | 7 | 2 |
| B14 | 9 | 6 | 4 | 6 | 13 | 5 | 2 | 10 | 10 | 0 | 10 | 8 | 19 | 4 |
| B15 | 1 | 1 | 0 | 0 | 4 | 0 | 0 | 4 | 9 | 0 | 2 | 13 | 6 | 0 |
| B16 | 15 | 8 | 0 | 4 | 17 | 8 | 5 | 21 | 17 | 0 | 14 | 46 | 23 | 4 |
| B17 | 3 | 3 | 1 | 3 | 3 | 2 | 2 | 2 | 2 | 0 | 4 | 5 | 4 | 2 |
| B18 | 7 | 3 | 0 | 2 | 19 | 4 | 6 | 10 | 49 | 0 | 6 | 51 | 17 | 4 |
| B19 | 26 | 18 | 10 | 8 | 25 | 17 | 19 | 19 | 25 | 0 | 23 | 38 | 29 | 17 |
| B20 | 8 | 5 | 1 | 2 | 9 | 5 | 4 | 12 | 19 | 0 | 12 | 32 | 15 | 3 |
| B21 | 95 | 68 | 15 | 38 | 150 | 63 | 49 | 84 | 11 | 13 | 81 | 63 | 150 | 54 |
| B22 | 6 | 4 | 2 | 2 | 5 | 4 | 1 | 6 | 3 | 0 | 6 | 11 | 8 | 0 |
| B23 | 12 | 7 | 1 | 5 | 11 | 11 | 6 | 5 | 5 | 0 | 8 | 6 | 10 | 4 |
| B24 | 2 | 1 | 0 | 1 | 5 | 2 | 2 | 3 | 3 | 0 | 2 | 3 | 5 | 2 |
| B25 | 1 | 2 | 1 | 0 | 1 | 2 | 2 | 1 | 6 | 0 | 0 | 5 | 2 | 2 |
| B26 | 4 | 3 | 0 | 2 | 7 | 4 | 3 | 3 | 0 | 0 | 3 | 1 | 7 | 4 |
| B27 | 5 | 3 | 0 | 1 | 2 | 2 | 1 | 0 | 1 | 0 | 3 | 0 | 1 | 1 |
| B28 | 21 | 7 | 0 | 3 | 16 | 9 | 2 | 15 | 0 | 0 | 13 | 10 | 25 | 1 |
| B29 | 10 | 1 | 0 | 2 | 3 | 3 | 1 | 1 | 0 | 0 | 6 | 1 | 4 | 0 |
| B30 | 4 | 4 | 0 | 2 | 4 | 3 | 3 | 5 | 1 | 0 | 6 | 5 | 5 | 3 |
| B31 | 4 | 4 | 0 | 3 | 7 | 4 | 3 | 5 | 2 | 0 | 5 | 6 | 6 | 3 |
| B32 | 7 | 5 | 0 | 5 | 8 | 6 | 4 | 7 | 3 | 0 | 5 | 5 | 7 | 6 |
| M1 | 14 | 10 | 2 | 5 | 13 | 11 | 13 | 9 | 3 | 0 | 13 | 10 | 11 | 9 |
| M2 | 72 | 40 | 20 | 20 | 78 | 29 | 15 | 82 | 8 | 0 | 89 | 77 | 98 | 2 |
| M3 | 12 | 6 | 2 | 3 | 7 | 7 | 11 | 7 | 2 | 0 | 15 | 12 | 8 | 3 |
| M4 | 61 | 14 | 0 | 6 | 14 | 12 | 5 | 12 | 8 | 0 | 17 | 10 | 25 | 0 |
| M5 | 21 | 17 | 1 | 5 | 19 | 13 | 10 | 8 | 3 | 0 | 17 | 8 | 15 | 6 |
| M6 | 57 | 51 | 2 | 8 | 106 | 48 | 49 | 22 | 10 | 4 | 33 | 29 | 50 | 65 |
| M7 | 0 | 0 | 0 | 0 | 0 | 0 | 0 | 3 | 3 | 0 | 1 | 10 | 3 | 0 |
| M8 | 0 | 0 | 0 | 0 | 0 | 0 | 0 | 0 | 1 | 0 | 0 | 10 | 1 | 0 |
| M9 | 13 | 5 | 0 | 4 | 6 | 5 | 0 | 12 | 0 | 0 | 16 | 12 | 9 | 0 |
| M10 | 4 | 2 | 0 | 1 | 5 | 4 | 4 | 3 | 2 | 0 | 3 | 3 | 5 | 2 |
| M11 | 0 | 0 | 1 | 0 | 2 | 0 | 1 | 0 | 6 | 0 | 1 | 6 | 1 | 2 |
| M12 | 35 | 19 | 5 | 9 | 28 | 12 | 23 | 11 | 0 | 0 | 19 | 5 | 19 | 37 |
| P1 | 3 | 3 | 3 | 3 | 3 | 0 | 0 | 3 | 0 | 0 | 4 | 13 | 3 | 0 |
| P2 | 0 | 2 | 0 | 0 | 0 | 0 | 0 | 2 | 0 | 0 | 2 | 34 | 6 | 0 |
| P3 | 5 | 1 | 3 | 1 | 5 | 2 | 0 | 5 | 0 | 0 | 4 | 8 | 7 | 0 |
| P4 | 4 | 2 | 4 | 1 | 4 | 1 | 0 | 4 | 0 | 0 | 4 | 6 | 4 | 0 |
| P5 | 6 | 1 | 0 | 1 | 1 | 1 | 0 | 2 | 0 | 0 | 3 | 3 | 8 | 0 |
| P6 | 14 | 0 | 0 | 0 | 5 | 0 | 0 | 0 | 0 | 0 | 0 | 0 | 11 | 0 |
| P7 | 21 | 1 | 2 | 0 | 6 | 3 | 0 | 4 | 0 | 0 | 5 | 8 | 10 | 0 |
| P8 | 0 | 0 | 1 | 0 | 7 | 0 | 0 | 1 | 0 | 0 | 0 | 0 | 7 | 2 |
| P9 | 6 | 1 | 2 | 1 | 3 | 2 | 0 | 4 | 0 | 0 | 7 | 6 | 4 | 0 |
| P10 | 19 | 2 | 1 | 1 | 13 | 2 | 0 | 11 | 0 | 0 | 11 | 11 | 15 | 0 |
| P11 | 19 | 11 | 4 | 9 | 7 | 11 | 4 | 8 | 0 | 0 | 14 | 9 | 11 | 0 |
| P12 | 7 | 2 | 0 | 3 | 7 | 2 | 4 | 3 | 0 | 0 | 5 | 5 | 4 | 1 |
| P13 | 0 | 1 | 20 | 1 | 0 | 1 | 1 | 3 | 20 | 0 | 4 | 19 | 1 | 0 |
| P14 | 5 | 0 | 1 | 1 | 2 | 0 | 0 | 6 | 0 | 0 | 3 | 4 | 7 | 0 |
| P15 | 11 | 2 | 0 | 1 | 1 | 0 | 0 | 7 | 0 | 0 | 7 | 5 | 8 | 0 |
| P16 | 18 | 1 | 0 | 1 | 3 | 5 | 0 | 1 | 0 | 0 | 15 | 2 | 2 | 0 |
| P17 | 12 | 2 | 0 | 1 | 7 | 0 | 0 | 4 | 0 | 0 | 6 | 3 | 11 | 0 |
| P18 | 1 | 0 | 0 | 0 | 6 | 0 | 0 | 0 | 0 | 0 | 0 | 1 | 1 | 0 |
| P19 | 9 | 0 | 0 | 0 | 16 | 0 | 0 | 2 | 0 | 0 | 5 | 0 | 16 | 0 |
| P20 | 2 | 1 | 0 | 2 | 6 | 3 | 2 | 5 | 3 | 0 | 4 | 15 | 7 | 0 |
| P21 | 42 | 20 | 2 | 17 | 20 | 28 | 0 | 14 | 0 | 0 | 34 | 25 | 29 | 0 |
| P22 | 0 | 1 | 1 | 0 | 10 | 1 | 0 | 2 | 0 | 0 | 1 | 6 | 12 | 0 |
| P23 | 7 | 0 | 1 | 0 | 2 | 2 | 0 | 4 | 0 | 0 | 8 | 3 | 5 | 0 |
| P24 | 5 | 13 | 0 | 2 | 108 | 9 | 0 | 15 | 0 | 0 | 4 | 46 | 81 | 4 |
| P25 | 0 | 0 | 0 | 0 | 0 | 0 | 0 | 0 | 0 | 0 | 0 | 24 | 0 | 0 |
| P26 | 3 | 0 | 9 | 0 | 1 | 0 | 0 | 7 | 0 | 0 | 1 | 30 | 2 | 0 |
| P27 | 0 | 15 | 0 | 1 | 1 | 4 | 5 | 0 | 0 | 0 | 0 | 0 | 0 | 7 |
| P28 | 3 | 0 | 0 | 0 | 24 | 1 | 0 | 0 | 1 | 0 | 0 | 5 | 14 | 2 |
| P29 | 7 | 4 | 1 | 2 | 3 | 3 | 1 | 2 | 0 | 0 | 3 | 3 | 3 | 0 |
| P30 | 0 | 0 | 6 | 0 | 0 | 0 | 0 | 1 | 4 | 0 | 1 | 8 | 0 | 0 |
| P31 | 4 | 2 | 58 | 0 | 1 | 0 | 2 | 3 | 0 | 0 | 1 | 10 | 0 | 1 |
| P32 | 1 | 0 | 19 | 1 | 0 | 1 | 1 | 1 | 0 | 0 | 1 | 0 | 0 | 0 |
| P33 | 0 | 0 | 3 | 0 | 0 | 0 | 0 | 0 | 7 | 0 | 0 | 9 | 0 | 0 |
| P34 | 13 | 0 | 0 | 0 | 7 | 1 | 0 | 11 | 0 | 0 | 7 | 7 | 22 | 0 |
| P35 | 4 | 0 | 3 | 0 | 8 | 0 | 0 | 11 | 0 | 0 | 1 | 102 | 10 | 0 |
| P36 | 40 | 60 | 2 | 17 | 10 | 55 | 7 | 9 | 3 | 0 | 21 | 6 | 11 | 6 |
| P37 | 88 | 23 | 8 | 10 | 24 | 22 | 2 | 32 | 1 | 0 | 41 | 40 | 43 | 0 |
| P38 | 28 | 4 | 5 | 2 | 20 | 1 | 0 | 27 | 0 | 0 | 20 | 44 | 41 | 0 |
| P39 | 8 | 1 | 2 | 1 | 8 | 1 | 0 | 8 | 0 | 0 | 9 | 10 | 12 | 0 |
| P40 | 31 | 64 | 8 | 61 | 28 | 119 | 21 | 11 | 33 | 0 | 27 | 72 | 20 | 14 |
| P41 | 16 | 163 | 31 | 17 | 60 | 12 | 0 | 60 | 0 | 0 | 49 | 315 | 250 | 3 |
| P42 | 88 | 109 | 43 | 16 | 141 | 33 | 8 | 100 | 69 | 0 | 81 | 272 | 320 | 2 |
| P43 | 39 | 96 | 42 | 48 | 37 | 84 | 67 | 22 | 18 | 8 | 51 | 54 | 36 | 34 |
| P44 | 112 | 20 | 4 | 16 | 82 | 27 | 5 | 61 | 0 | 0 | 70 | 52 | 87 | 0 |
| P45 | 92 | 127 | 53 | 12 | 113 | 39 | 8 | 115 | 2 | 0 | 70 | 161 | 227 | 5 |
| P46 | 70 | 16 | 9 | 9 | 25 | 17 | 4 | 19 | 6 | 0 | 53 | 18 | 36 | 0 |
| P47 | 1 | 2 | 5 | 2 | 1 | 0 | 0 | 0 | 1 | 0 | 0 | 0 | 0 | 12 |
| P48 | 3 | 0 | 10 | 0 | 6 | 2 | 0 | 2 | 1 | 0 | 1 | 30 | 9 | 7 |
| P49 | 0 | 11 | 1 | 1 | 15 | 1 | 11 | 0 | 4 | 0 | 0 | 5 | 3 | 25 |
| P50 | 1 | 0 | 0 | 0 | 40 | 0 | 0 | 0 | 0 | 1 | 0 | 0 | 0 | 28 |
| P51 | 21 | 18 | 17 | 7 | 65 | 8 | 13 | 23 | 43 | 3 | 16 | 84 | 59 | 9 |
| P52 | 77 | 56 | 19 | 30 | 187 | 48 | 38 | 44 | 192 | 3 | 41 | 98 | 93 | 44 |
| P53 | 71 | 3 | 2 | 1 | 3 | 2 | 1 | 30 | 15 | 0 | 64 | 13 | 26 | 0 |
| P54 | 2 | 3 | 2 | 13 | 4 | 8 | 5 | 1 | 5 | 0 | 2 | 1 | 4 | 9 |
| P55 | 54 | 29 | 0 | 10 | 9 | 26 | 2 | 14 | 0 | 0 | 21 | 47 | 14 | 0 |
| P56 | 35 | 114 | 20 | 60 | 19 | 79 | 6 | 13 | 1 | 0 | 34 | 12 | 13 | 6 |
| P57 | 10 | 34 | 4 | 17 | 11 | 19 | 7 | 3 | 105 | 0 | 2 | 8 | 5 | 12 |
| P58 | 26 | 40 | 40 | 30 | 33 | 40 | 19 | 25 | 82 | 0 | 37 | 103 | 41 | 13 |
| P59 | 21 | 6 | 0 | 2 | 3 | 3 | 0 | 2 | 2 | 0 | 17 | 2 | 3 | 0 |
| P60 | 0 | 1 | 0 | 0 | 5 | 0 | 1 | 2 | 0 | 0 | 1 | 2 | 8 | 8 |
| P61 | 8 | 7 | 0 | 4 | 4 | 6 | 2 | 3 | 0 | 0 | 6 | 2 | 3 | 0 |
| P62 | 5 | 3 | 0 | 3 | 2 | 3 | 0 | 1 | 0 | 0 | 5 | 2 | 1 | 0 |
| P63 | 0 | 0 | 1 | 0 | 1 | 0 | 0 | 1 | 4 | 0 | 0 | 7 | 2 | 0 |
| P64 | 0 | 4 | 3 | 2 | 1 | 5 | 2 | 1 | 0 | 0 | 0 | 2 | 0 | 0 |
| P65 | 1 | 7 | 5 | 1 | 3 | 3 | 1 | 0 | 0 | 0 | 2 | 16 | 6 | 1 |
| P66 | 9 | 26 | 0 | 8 | 13 | 20 | 5 | 4 | 5 | 0 | 5 | 11 | 11 | 2 |
| S1 | 24 | 31 | 1 | 9 | 4 | 29 | 13 | 6 | 0 | 0 | 16 | 4 | 9 | 13 |
| S2 | 7 | 7 | 0 | 1 | 3 | 5 | 0 | 5 | 3 | 0 | 6 | 5 | 8 | 0 |
| S3 | 6 | 7 | 0 | 2 | 3 | 3 | 3 | 2 | 1 | 0 | 4 | 3 | 5 | 4 |
| S4 | 1 | 1 | 0 | 1 | 0 | 0 | 0 | 2 | 0 | 0 | 1 | 13 | 2 | 0 |
| S5 | 7 | 13 | 2 | 2 | 3 | 4 | 3 | 6 | 0 | 0 | 5 | 14 | 5 | 2 |
| S6 | 6 | 4 | 9 | 2 | 3 | 4 | 4 | 3 | 0 | 0 | 6 | 2 | 4 | 0 |
| S7 | 1 | 0 | 6 | 0 | 0 | 0 | 0 | 2 | 0 | 0 | 1 | 1 | 1 | 0 |
| S8 | 1 | 1 | 27 | 0 | 0 | 1 | 1 | 1 | 0 | 0 | 2 | 0 | 0 | 0 |
| S9 | 9 | 3 | 129 | 0 | 3 | 1 | 0 | 20 | 1 | 0 | 12 | 48 | 25 | 0 |
| S10 | 0 | 2 | 0 | 0 | 25 | 0 | 8 | 0 | 0 | 0 | 0 | 0 | 0 | 90 |
| S11 | 1 | 2 | 0 | 0 | 0 | 0 | 2 | 0 | 0 | 0 | 0 | 0 | 0 | 7 |
| S12 | 5 | 0 | 0 | 1 | 1 | 2 | 1 | 3 | 0 | 0 | 1 | 2 | 8 | 0 |
| S13 | 0 | 0 | 0 | 0 | 0 | 0 | 0 | 0 | 25 | 0 | 0 | 29 | 0 | 0 |
| S14 | 0 | 0 | 8 | 0 | 0 | 0 | 0 | 0 | 1 | 0 | 0 | 14 | 0 | 0 |
| S15 | 11 | 7 | 0 | 8 | 35 | 11 | 4 | 16 | 0 | 0 | 7 | 0 | 15 | 3 |
| S16 | 0 | 0 | 0 | 0 | 0 | 0 | 0 | 0 | 2 | 0 | 0 | 17 | 0 | 0 |
| S17 | 8 | 6 | 12 | 4 | 1 | 12 | 8 | 1 | 0 | 0 | 2 | 0 | 2 | 5 |
| S18 | 41 | 25 | 21 | 14 | 18 | 27 | 11 | 10 | 10 | 0 | 33 | 12 | 25 | 7 |
| S19 | 6 | 5 | 2 | 5 | 1 | 3 | 3 | 1 | 0 | 0 | 5 | 1 | 2 | 1 |
| S20 | 0 | 0 | 0 | 0 | 0 | 0 | 0 | 0 | 0 | 0 | 0 | 16 | 0 | 0 |
| S21 | 0 | 0 | 1 | 0 | 1 | 0 | 2 | 1 | 6 | 0 | 0 | 9 | 1 | 5 |
| S22 | 9 | 2 | 39 | 2 | 2 | 1 | 0 | 4 | 0 | 0 | 22 | 4 | 8 | 0 |
| S23 | 8 | 12 | 28 | 4 | 3 | 11 | 13 | 7 | 0 | 0 | 10 | 3 | 5 | 13 |
| S24 | 12 | 11 | 1 | 1 | 2 | 4 | 3 | 8 | 2 | 0 | 13 | 22 | 10 | 2 |
