# Supplementary material for: Diversification dynamics in the Neotropics through time, clades, and biogeographic regions
Source: eLife. 2022 Oct 27;11:e74503. doi: 10.7554/eLife.74503 (PMC9668338; doi:10.7554/eLife.74503)
Supplement: Figure 6—source data 2. [file elife-74503-fig6-data2.docx]

**Figure 6 - Source Data 2.**

For each of the 150 phylogenetic clades considered in this study, we provide the proportion of species occurring in the 5 main biogeographic clusters identified here based on K-means clustering algorithms: cluster 1 (including the Amazonia, Central Andes, Chocó, Guiana Shield, Mesoamerica, and Northern Andes), cluster 2 (Atlantic Forest, Caatinga, Cerrado, Chaco, and temperate South America), cluster 3 (Bahama-Antilles), cluster 4 (“elsewhere” region), or cluster 5 (Galapagos). Clades were assigned to a given cluster only if > 60% of the species in the clade are distributed in the cluster, otherwise clades are classified as ‘mixed’ (main cluster 0). Clade numbers correspond with Figure-2-source-data-1. Cluster numbers correspond with Figure 6.

| Clade | cluster-1 | cluster-2 | cluster-3 | cluster-4 | cluster-5 | Assignation to cluster |
| --- | --- | --- | --- | --- | --- | --- |
| A1 | 0.98 | 0.01 | 0 | 0 | 0.01 | 1 |
| A10 | 0.68 | 0.23 | 0.01 | 0 | 0.08 | 1 |
| A11 | 0.28 | 0.72 | 0 | 0 | 0 | 2 |
| A12 | 0.48 | 0.51 | 0 | 0 | 0.01 | 0 |
| A13 | 0.99 | 0.01 | 0 | 0 | 0 | 1 |
| A14 | 0.98 | 0.02 | 0 | 0 | 0 | 1 |
| A15 | 0.56 | 0.28 | 0.16 | 0 | 0 | 0 |
| A16 | 0.95 | 0 | 0.05 | 0 | 0 | 1 |
| A2 | 0.97 | 0.03 | 0 | 0 | 0 | 1 |
| A3 | 0.84 | 0.16 | 0 | 0 | 0 | 1 |
| A4 | 0.63 | 0.37 | 0 | 0 | 0 | 1 |
| A5 | 0.34 | 0 | 0.01 | 0 | 0.65 | 5 |
| A6 | 0.96 | 0.03 | 0 | 0 | 0.01 | 1 |
| A7 | 0.55 | 0.45 | 0 | 0 | 0 | 0 |
| A8 | 0.6 | 0.4 | 0 | 0 | 0.01 | 1 |
| A9 | 0.61 | 0.39 | 0 | 0 | 0 | 1 |
| B1 | 0.73 | 0.24 | 0.04 | 0 | 0 | 1 |
| B10 | 0.63 | 0.34 | 0.03 | 0 | 0 | 1 |
| B11 | 0.79 | 0.21 | 0 | 0 | 0 | 1 |
| B12 | 0.72 | 0.02 | 0.25 | 0 | 0.02 | 1 |
| B13 | 0.62 | 0.21 | 0.17 | 0 | 0 | 1 |
| B14 | 0.65 | 0.22 | 0.09 | 0 | 0.04 | 1 |
| B15 | 0.75 | 0.03 | 0.22 | 0 | 0 | 1 |
| B16 | 0.75 | 0.16 | 0.09 | 0 | 0 | 1 |
| B17 | 0.58 | 0.33 | 0.06 | 0 | 0.03 | 0 |
| B18 | 0.62 | 0.11 | 0.28 | 0 | 0 | 1 |
| B19 | 0.58 | 0.29 | 0.09 | 0 | 0.04 | 0 |
| B2 | 0.78 | 0.17 | 0.03 | 0 | 0.01 | 1 |
| B20 | 0.69 | 0.15 | 0.15 | 0 | 0.01 | 1 |
| B21 | 0.67 | 0.29 | 0.01 | 0.01 | 0.02 | 1 |
| B22 | 0.72 | 0.19 | 0.05 | 0 | 0.03 | 1 |
| B23 | 0.57 | 0.36 | 0.05 | 0 | 0.01 | 0 |
| B24 | 0.65 | 0.26 | 0.1 | 0 | 0 | 1 |
| B25 | 0.4 | 0.32 | 0.24 | 0 | 0.04 | 0 |
| B26 | 0.61 | 0.39 | 0 | 0 | 0 | 1 |
| B27 | 0.55 | 0.4 | 0.05 | 0 | 0 | 0 |
| B28 | 0.82 | 0.18 | 0 | 0 | 0 | 1 |
| B29 | 0.78 | 0.22 | 0 | 0 | 0 | 1 |
| B3 | 0.5 | 0.5 | 0 | 0 | 0 | 0 |
| B30 | 0.64 | 0.33 | 0.02 | 0 | 0 | 1 |
| B31 | 0.63 | 0.33 | 0.04 | 0 | 0 | 1 |
| B32 | 0.57 | 0.38 | 0.04 | 0 | 0 | 0 |
| B4 | 0.59 | 0.32 | 0.05 | 0 | 0.03 | 0 |
| B5 | 0.71 | 0.29 | 0 | 0 | 0 | 1 |
| B6 | 0.55 | 0.45 | 0 | 0 | 0 | 0 |
| B7 | 0.73 | 0.27 | 0 | 0 | 0 | 1 |
| B8 | 0.78 | 0.22 | 0 | 0 | 0 | 1 |
| B9 | 0.64 | 0.36 | 0 | 0 | 0 | 1 |
| M1 | 0.57 | 0.39 | 0.02 | 0 | 0.02 | 0 |
| M10 | 0.61 | 0.34 | 0.05 | 0 | 0 | 1 |
| M11 | 0.5 | 0.15 | 0.3 | 0 | 0.05 | 0 |
| M12 | 0.53 | 0.45 | 0 | 0 | 0.02 | 0 |
| M2 | 0.79 | 0.17 | 0.01 | 0 | 0.03 | 1 |
| M3 | 0.64 | 0.32 | 0.02 | 0 | 0.02 | 1 |
| M4 | 0.76 | 0.2 | 0.04 | 0 | 0 | 1 |
| M5 | 0.62 | 0.36 | 0.02 | 0 | 0.01 | 1 |
| M6 | 0.56 | 0.41 | 0.02 | 0.01 | 0 | 0 |
| M7 | 0.85 | 0 | 0.15 | 0 | 0 | 1 |
| M8 | 0.92 | 0 | 0.08 | 0 | 0 | 1 |
| M9 | 0.83 | 0.17 | 0 | 0 | 0 | 1 |
| P1 | 0.76 | 0.16 | 0 | 0 | 0.08 | 1 |
| P10 | 0.93 | 0.06 | 0 | 0 | 0.01 | 1 |
| P11 | 0.64 | 0.33 | 0 | 0 | 0.04 | 1 |
| P12 | 0.72 | 0.28 | 0 | 0 | 0 | 1 |
| P13 | 0.38 | 0.06 | 0.28 | 0 | 0.28 | 0 |
| P14 | 0.93 | 0.03 | 0 | 0 | 0.03 | 1 |
| P15 | 0.93 | 0.07 | 0 | 0 | 0 | 1 |
| P16 | 0.85 | 0.15 | 0 | 0 | 0 | 1 |
| P17 | 0.93 | 0.07 | 0 | 0 | 0 | 1 |
| P18 | 1 | 0 | 0 | 0 | 0 | 1 |
| P19 | 1 | 0 | 0 | 0 | 0 | 1 |
| P2 | 0.96 | 0.04 | 0 | 0 | 0 | 1 |
| P20 | 0.78 | 0.16 | 0.06 | 0 | 0 | 1 |
| P21 | 0.71 | 0.28 | 0 | 0 | 0.01 | 1 |
| P22 | 0.91 | 0.06 | 0 | 0 | 0.03 | 1 |
| P23 | 0.91 | 0.06 | 0 | 0 | 0.03 | 1 |
| P24 | 0.9 | 0.1 | 0 | 0 | 0 | 1 |
| P25 | 1 | 0 | 0 | 0 | 0 | 1 |
| P26 | 0.83 | 0 | 0 | 0 | 0.17 | 1 |
| P27 | 0.03 | 0.97 | 0 | 0 | 0 | 2 |
| P28 | 0.92 | 0.06 | 0.02 | 0 | 0 | 1 |
| P29 | 0.66 | 0.31 | 0 | 0 | 0.03 | 1 |
| P3 | 0.83 | 0.1 | 0 | 0 | 0.07 | 1 |
| P30 | 0.5 | 0 | 0.2 | 0 | 0.3 | 0 |
| P31 | 0.23 | 0.06 | 0 | 0 | 0.71 | 5 |
| P32 | 0.12 | 0.12 | 0 | 0 | 0.76 | 5 |
| P33 | 0.47 | 0 | 0.37 | 0 | 0.16 | 0 |
| P34 | 0.99 | 0.01 | 0 | 0 | 0 | 1 |
| P35 | 0.98 | 0 | 0 | 0 | 0.02 | 1 |
| P36 | 0.39 | 0.59 | 0.01 | 0 | 0.01 | 0 |
| P37 | 0.8 | 0.17 | 0 | 0 | 0.02 | 1 |
| P38 | 0.94 | 0.04 | 0 | 0 | 0.03 | 1 |
| P39 | 0.92 | 0.05 | 0 | 0 | 0.03 | 1 |
| P4 | 0.76 | 0.12 | 0 | 0 | 0.12 | 1 |
| P40 | 0.37 | 0.55 | 0.06 | 0 | 0.02 | 0 |
| P41 | 0.77 | 0.2 | 0 | 0 | 0.03 | 1 |
| P42 | 0.78 | 0.13 | 0.05 | 0 | 0.03 | 1 |
| P43 | 0.38 | 0.52 | 0.03 | 0.01 | 0.07 | 0 |
| P44 | 0.87 | 0.13 | 0 | 0 | 0.01 | 1 |
| P45 | 0.76 | 0.19 | 0 | 0 | 0.05 | 1 |
| P46 | 0.78 | 0.16 | 0.02 | 0 | 0.03 | 1 |
| P47 | 0.08 | 0.67 | 0.04 | 0 | 0.21 | 2 |
| P48 | 0.72 | 0.13 | 0.01 | 0 | 0.14 | 1 |
| P49 | 0.3 | 0.64 | 0.05 | 0 | 0.01 | 2 |
| P5 | 0.88 | 0.12 | 0 | 0 | 0 | 1 |
| P50 | 0.59 | 0.4 | 0 | 0.01 | 0 | 0 |
| P51 | 0.69 | 0.14 | 0.11 | 0.01 | 0.04 | 1 |
| P52 | 0.56 | 0.22 | 0.2 | 0 | 0.02 | 0 |
| P53 | 0.9 | 0.03 | 0.06 | 0 | 0.01 | 1 |
| P54 | 0.24 | 0.64 | 0.08 | 0 | 0.03 | 2 |
| P55 | 0.7 | 0.3 | 0 | 0 | 0 | 1 |
| P56 | 0.31 | 0.64 | 0 | 0 | 0.05 | 2 |
| P57 | 0.16 | 0.38 | 0.44 | 0 | 0.02 | 0 |
| P58 | 0.5 | 0.27 | 0.16 | 0 | 0.08 | 0 |
| P59 | 0.79 | 0.18 | 0.03 | 0 | 0 | 1 |
| P6 | 1 | 0 | 0 | 0 | 0 | 1 |
| P60 | 0.64 | 0.36 | 0 | 0 | 0 | 1 |
| P61 | 0.58 | 0.42 | 0 | 0 | 0 | 0 |
| P62 | 0.64 | 0.36 | 0 | 0 | 0 | 1 |
| P63 | 0.69 | 0 | 0.25 | 0 | 0.06 | 1 |
| P64 | 0.2 | 0.65 | 0 | 0 | 0.15 | 2 |
| P65 | 0.61 | 0.28 | 0 | 0 | 0.11 | 1 |
| P66 | 0.45 | 0.51 | 0.04 | 0 | 0 | 0 |
| P7 | 0.9 | 0.07 | 0 | 0 | 0.03 | 1 |
| P8 | 0.83 | 0.11 | 0 | 0 | 0.06 | 1 |
| P9 | 0.83 | 0.11 | 0 | 0 | 0.06 | 1 |
| S1 | 0.4 | 0.6 | 0 | 0 | 0.01 | 2 |
| S10 | 0.2 | 0.8 | 0 | 0 | 0 | 2 |
| S11 | 0.08 | 0.92 | 0 | 0 | 0 | 2 |
| S12 | 0.83 | 0.17 | 0 | 0 | 0 | 1 |
| S13 | 0.54 | 0 | 0.46 | 0 | 0 | 0 |
| S14 | 0.61 | 0 | 0.04 | 0 | 0.35 | 1 |
| S15 | 0.72 | 0.28 | 0 | 0 | 0 | 1 |
| S16 | 0.89 | 0 | 0.11 | 0 | 0 | 1 |
| S17 | 0.23 | 0.57 | 0 | 0 | 0.2 | 0 |
| S18 | 0.55 | 0.33 | 0.04 | 0 | 0.08 | 0 |
| S19 | 0.46 | 0.49 | 0 | 0 | 0.06 | 0 |
| S2 | 0.68 | 0.26 | 0.06 | 0 | 0 | 1 |
| S20 | 1 | 0 | 0 | 0 | 0 | 1 |
| S21 | 0.46 | 0.27 | 0.23 | 0 | 0.04 | 0 |
| S22 | 0.53 | 0.05 | 0 | 0 | 0.42 | 0 |
| S23 | 0.31 | 0.45 | 0 | 0 | 0.24 | 0 |
| S24 | 0.74 | 0.23 | 0.02 | 0 | 0.01 | 1 |
| S3 | 0.53 | 0.44 | 0.02 | 0 | 0 | 0 |
| S4 | 0.9 | 0.1 | 0 | 0 | 0 | 1 |
| S5 | 0.61 | 0.36 | 0 | 0 | 0.03 | 1 |
| S6 | 0.51 | 0.3 | 0 | 0 | 0.19 | 0 |
| S7 | 0.5 | 0 | 0 | 0 | 0.5 | 0 |
| S8 | 0.12 | 0.09 | 0 | 0 | 0.79 | 5 |
| S9 | 0.47 | 0.02 | 0 | 0 | 0.51 | 0 |
