# Supplementary material for: Diversification dynamics in the Neotropics through time, clades, and biogeographic regions
Source: eLife. 2022 Oct 27;11:e74503. doi: 10.7554/eLife.74503 (PMC9668338; doi:10.7554/eLife.74503)
Supplement: Figure 6—source data 3. [file elife-74503-fig6-data3.docx]

**Figure 6 - Source data 3.**

Source data for Figure 6c,d,f, and Figure 7, 8, and 9. Tree size, sampling fraction, crown age, diversification (*r*), speciation (*𝛌*) and extinction (*µ*) rates (based on the constant diversification model) and species richness dynamics (gradual increase [Sc. 1: Cst.], exponential increase [Sc. 2: Expo.], saturated increase [Sc. 3: Saturat.] and decline [Sc. 4: Dec.]). In addition, for each clade, we provide the main elevational range (and an alternative codification [Elev. 2] with the lowland and montane categories pooled together; see text) and its assignation to 5 or 7 biogeographic clusters, respectively. Abbreviations: Mixed = Mix., Lowland = L.; Montane = M., Highland = H.; Exponential = Expo.; Constant = Cst.; Saturated = Saturat.; Decline = Dec.

| Clade | Tree size | Sampling  fraction | Crown.age | *r* | *𝛌* | *µ* | Richness | Elevation | Elev. 2 | 5 cluster | 7 cluster |
| --- | --- | --- | --- | --- | --- | --- | --- | --- | --- | --- | --- |
| P1 | 13 | 0.116 | 2.88 | 1.558 | 2.182 | 0.624 | Expo. | Mix. | L-M | 1 | 0 |
| P2 | 38 | 0.279 | 0.936 | 5.272 | 6.689 | 1.417 | Expo. | Mix. | L-M | 1 | 3 |
| P3 | 12 | 0.158 | 43.022 | 0.068 | 0.1537 | 0.085 | Cst. | Mix. | L-M | 1 | 1 |
| P4 | 10 | 0.125 | 13.8 | 0.133 | 1.02 | 0.890 | Cst. | Mix. | L-M | 1 | 0 |
| P5 | 10 | 0.125 | 7.32 | 0.317 | 1.354 | 1.036 | Cst. | Mix. | L-M | 1 | 1 |
| P6 | 14 | 0.452 | 5.818 | 0.471 | 0.471 | 0 | Cst. | Mix. | L-M | 1 | 1 |
| P7 | 29 | 0.569 | 12.272 | 0.290 | 0.290 | 0 | Cst. | Mix. | L-M | 1 | 1 |
| P8 | 15 | 0.139 | 11.88 | 0.151 | 1.860 | 1.709 | Expo. | M | L-M | 1 | 1 |
| P9 | 10 | 0.153 | 19.626 | 0.176 | 0.175 | 0 | Cst. | Mix. | L-M | 1 | 1 |
| P10 | 24 | 0.407 | 25.524 | 0.136 | 0.136 | 0 | Cst. | Mix. | L-M | 1 | 1 |
| P11 | 42 | 0.682 | 15.153 | 0.184 | 0.429 | 0.244 | Cst. | Mix. | L-M | 1 | 0 |
| P12 | 15 | 0.349 | 13.044 | 0.223 | 0.298 | 0.075 | Cst. | Mix. | L-M | 1 | 1 |
| P13 | 46 | 0.321 | 56.306 | 0.042 | 0.161 | 0.119 | Dec. | L | L-M | 0 | 0 |
| P14 | 10 | 0.200 | 26.003 | 0.052 | 0.43 | 0.378 | Cst. | L | L-M | 1 | 1 |
| P15 | 19 | 0.630 | 19.876 | 0.134 | 0.185 | 0.051 | Cst. | L | L-M | 1 | 1 |
| P16 | 19 | 0.207 | 7.085 | 0.449 | 1.140 | 0.691 | Cst. | L | L-M | 1 | 1 |
| P17 | 18 | 0.182 | 22.422 | 0.131 | 0.270 | 0.139 | Cst. | Mix. | L-M | 1 | 1 |
| P18 | 7 | 0.875 | 10.802 | 0.113 | 0.113 | 0 | Cst. | M | L-M | 1 | 1 |
| P19 | 22 | 0.18 | 28.016 | 0.1638 | 0.237 | 0.073 | Expo. | Mix. | L-M | 1 | 1 |
| P20 | 29 | 0.725 | 20.251 | 0.145 | 0.145 | 0 | Cst. | Mix. | L-M | 1 | 0 |
| P21 | 103 | 0.406 | 20.768 | 0.185 | 0.185 | 0 | Dec. | L | L-M | 1 | 1 |
| P22 | 19 | 0.432 | 17.636 | 0.172 | 0.171 | 0 | Cst. | M-H | M-H | 1 | 1 |
| P23 | 9 | 0.562 | 25.054 | 0.058 | 0.130 | 0.072 | Cst. | L | L-M | 1 | 1 |
| P24 | 200 | 0.364 | 17.027 | 0.349 | 2.758 | 2.409 | Expo. | Mix. | Mix. | 1 | 1 |
| P25 | 24 | 0.889 | 19.209 | 0.138 | 0.138 | 0 | Cst. | M | L-M | 1 | 3 |
| P26 | 43 | 0.606 | 14.632 | 0.225 | 0.225 | 0 | Cst. | Mix. | L-M | 1 | 0 |
| P27 | 19 | 0.543 | 4.666 | 0.574 | 0.744 | 0.170 | Cst. | Mix. | L-M | 2 | 2 |
| P28 | 37 | 0.285 | 2.755 | 1.976 | 2.092 | 0.116 | Expo. | M-H | M-H | 1 | 1 |
| P29 | 13 | 0.933 | 15.285 | 0.107 | 0.107 | 0 | Saturat. | L | L-M | 1 | 0 |
| P30 | 14 | 0.875 | 37.321 | 0.035 | 0.098 | 0.062 | Cst. | L | L-M | 0 | 0 |
| P31 | 78 | 0.975 | 30.969 | 0.106 | 0.187 | 0.081 | Cst. | Mix. | L-M | 5 | 7 |
| P32 | 22 | 0.786 | 20.511 | 0.137 | 0.136 | 0 | Cst. | L | L-M | 5 | 7 |
| P33 | 16 | 1 | 19.96 | 0.089 | 0.089 | 0 | Cst. | Mix. | L-M | 0 | 0 |
| P34 | 32 | 1 | 29.675 | 0.064 | 0.205 | 0.141 | Cst. | Mix. | L-M | 1 | 1 |
| P35 | 114 | 0.991 | 38.071 | 0.130 | 0.130 | 0 | Saturat. | Mix. | L-M | 1 | 3 |
| P36 | 154 | 0.987 | 27.09 | 0.195 | 0.195 | 0.000 | Expo. | Mix. | L-M | 0 | 0 |
| P37 | 178 | 0.989 | 43.622 | 0.177 | 0.177 | 0 | Dec. | Mix. | L-M | 1 | 1 |
| P38 | 102 | 1 | 26.923 | 0.202 | 0.253 | 0.050 | Expo. | Mix. | L-M | 1 | 1 |
| P39 | 22 | 1 | 34.208 | 0.007 | 0.263 | 0.255 | Cst. | Mix. | L-M | 1 | 1 |
| P40 | 271 | 0.456 | 20.391 | 0.288 | 0.883 | 0.595 | Expo. | Mix. | L-M | 0 | 0 |
| P41 | 670 | 0.131 | 17.869 | 0.314 | 3.079 | 2.764 | Cst. | M-H | M-H | 1 | 0 |
| P42 | 789 | 0.213 | 31.481 | 0.243 | 0.865 | 0.622 | Expo. | Mix. | Mix. | 1 | 0 |
| P43 | 168 | 0.247 | 21.008 | 0.287 | 0.836 | 0.548 | Expo. | Mix. | L-M | 0 | 0 |
| P44 | 126 | 0.331 | 6 | 1.193 | 4.850 | 3.657 | Expo. | Mix. | L-M | 1 | 1 |
| P45 | 588 | 0.49 | 48.448 | 0.167 | 0.166 | 0 | Expo. | M | L-M | 1 | 1 |
| P46 | 111 | 0.793 | 28.918 | 0.155 | 0.155 | 0 | Saturat. | L | L-M | 1 | 1 |
| P47 | 21 | 1 | 28.86 | -0.002 | 0.338 | 0.340 | Cst. | Mix. | L-M | 2 | 0 |
| P48 | 50 | 0.226 | 32.21 | 0.002 | 3.132 | 3.13 | Cst. | Mix. | L-M | 1 | 0 |
| P49 | 44 | 0.301 | 30.992 | 0.041 | 1.176 | 1.135 | Cst. | M-H | M-H | 2 | 0 |
| P50 | 63 | 0.708 | 7.206 | 0.594 | 0.593 | 0 | Cst. | L | L-M | 0 | 0 |
| P51 | 189 | 0.442 | 21.785 | 0.201 | 0.585 | 0.384 | Expo. | Mix. | L-M | 1 | 0 |
| P52 | 495 | 0.319 | 20.793 | 0.345 | 0.702 | 0.356 | Expo. | Mix. | Mix. | 0 | 0 |
| P53 | 86 | 0.77 | 30.276 | 0.149 | 0.149 | 0 | Cst. | L | L-M | 1 | 1 |
| P54 | 38 | 0.559 | 45.013 | 0.059 | 0.191 | 0.131 | Dec. | Mix. | L-M | 2 | 0 |
| P55 | 120 | 0.458 | 19.5 | 0.157 | 1.605 | 1.448 | Expo. | L | L-M | 1 | 0 |
| P56 | 150 | 0.251 | 27.879 | 0.200 | 0.251 | 0.051 | Dec. | Mix. | L-M | 2 | 2 |
| P57 | 161 | 0.25 | 48.279 | 0.118 | 0.183 | 0.065 | Cst. | L | L-M | 0 | 0 |
| P58 | 307 | 0.24 | 42.379 | 0.123 | 0.526 | 0.403 | Expo. | Mix. | L-M | 0 | 0 |
| P59 | 34 | 0.351 | 24.169 | 0.139 | 0.606 | 0.467 | Expo. | L | L-M | 1 | 1 |
| P60 | 16 | 0.182 | 35.745 | 0.077 | 314 | 0.236 | Expo. | Mix. | L-M | 1 | 0 |
| P61 | 14 | 0.127 | 8.684 | 0.438 | 0.513 | 0.074 | Cst. | L | L-M | 0 | 0 |
| P62 | 10 | 0.4 | 1.647 | 1.695 | 1.695 | 0 | Cst. | L | L-M | 1 | 0 |
| P63 | 10 | 0.417 | 0.878 | 2.227 | 2.226 | 0 | Cst. | Mix. | L-M | 1 | 0 |
| P64 | 10 | 0.588 | 4.566 | 0.415 | 0.415 | 0 | Cst. | L | L-M | 2 | 0 |
| P65 | 32 | 0.107 | 21.506 | 0.160 | 0.706 | 0.546 | Cst. | M-H | M-H | 1 | 0 |
| P66 | 48 | 0.276 | 0.497 | 8.821 | 8.821 | 0 | Cst. | Mix. | L-M | 0 | 0 |
| M1 | 32 | 1 | 67.956 | 0.028 | 0.077 | 0.048 | Cst. | L | L-M | 0 | 0 |
| M2 | 192 | 0.86 | 41.856 | 0.123 | 0.123 | 0,000 | Saturat. | L | L-M | 1 | 1 |
| M3 | 20 | 0.69 | 22.502 | 0.089 | 0.089 | 0 | Dec. | L | L-M | 1 | 0 |
| M4 | 95 | 0.477 | 21.539 | 0.099 | 1.066 | 0.967 | Expo. | L | L-M | 1 | 1 |
| M5 | 43 | 0.417 | 25.493 | 0.133 | 0.249 | 0.116 | Cst. | Mix. | L-M | 1 | 0 |
| M6 | 279 | 0.676 | 12.657 | 0.439 | 0.439 | 0 | Saturat. | Mix. | Mix. | 0 | 0 |
| M7 | 11 | 0.688 | 10.6 | 0.173 | 0.173 | 0 | Cst. | L | L-M | 1 | 0 |
| M8 | 10 | 0.455 | 13.809 | 0.045 | 0.452 | 0.406 | Cst. | L | L-M | 1 | 3 |
| M9 | 20 | 0.909 | 34.902 | 0.049 | 0.049 | 0 | Saturat. | Mix. | L-M | 1 | 1 |
| M10 | 11 | 0.579 | 8.544 | 0.233 | 0.233 | 0 | Cst. | Mix. | Mix. | 1 | 0 |
| M11 | 10 | 1 | 16 | 0.104 | 0.104 | 0 | Cst. | L | L-M | 0 | 0 |
| M12 | 199 | 0.816 | 35.28 | 0.131 | 265 | 0.133 | Dec. | L | L-M | 0 | 0 |
| B1 | 39 | 0.71 | 11.823 | 0.334 | 0.3349 | 0 | Cst. | Mix. | L-M | 1 | 1 |
| B2 | 233 | 0.69 | 26.097 | 0.180 | 0.180 | 0 | Saturat. | Mix. | Mix. | 1 | 1 |
| B3 | 8 | 0.73 | 24.786 | -0.063 | 0.299 | 0.359 | Cst. | Mix. | L-M | 0 | 0 |
| B4 | 118 | 0.71 | 28.953 | 0.169 | 0.184 | 0.015 | Cst. | Mix. | Mix. | 0 | 0 |
| B5 | 55 | 0.49 | 25.166 | 0.152 | 0.152 | 0 | Saturat. | Mix. | L-M | 1 | 1 |
| B6 | 9 | 0.6 | 23.421 | 0.091 | 0.091 | 0 | Cst. | M | L-M | 0 | 0 |
| B7 | 7 | 0.64 | 18.548 | 0.004 | 0.203 | 0.198 | Cst. | L | L-M | 1 | 1 |
| B8 | 165 | 0.71 | 16.825 | 0.209 | 0.209 | 0 | Saturat. | L | L-M | 1 | 1 |
| B9 | 292 | 0.94 | 32.586 | 0.149 | 0.149 | 0 | Saturat. | Mix. | Mix. | 1 | 0 |
| B10 | 316 | 0.79 | 25.501 | 0.175 | 0.175 | 0 | Saturat. | Mix. | Mix. | 1 | 0 |
| B11 | 80 | 0.68 | 23.515 | 0.180 | 0.180 | 0 | Cst. | L | L-M | 1 | 1 |
| B12 | 19 | 0.37 | 20.151 | 0.154 | 0.197 | 0.043 | Cst. | Mix. | L-M | 1 | 0 |
| B13 | 36 | 0.95 | 17.382 | 0.145 | 0.145 | 0 | Saturat. | Mix. | L-M | 1 | 0 |
| B14 | 39 | 0.85 | 8.199 | 0.225 | 0.225 | 0 | Saturat. | Mix. | Mix. | 1 | 0 |
| B15 | 17 | 0.94 | 13.105 | 0.137 | 0.137 | 0 | Cst. | Mix. | L-M | 1 | 0 |
| B16 | 67 | 0.62 | 24.509 | 0.158 | 0.158 | 0 | Saturat. | L | L-M | 1 | 0 |
| B17 | 10 | 0.37 | 13.101 | 0.185 | 0.1854 | 0 | Cst. | L | L-M | 0 | 0 |
| B18 | 89 | 0.99 | 11.208 | 0.216 | 0.216 | 0 | Dec. | Mix. | L-M | 1 | 0 |
| B19 | 92 | 0.89 | 12.021 | 0.265 | 0.265 | 0 | Saturat. | Mix. | L-M | 0 | 0 |
| B20 | 41 | 0.82 | 18.038 | 0.121 | 0.121 | 0 | Saturat. | Mix. | L-M | 1 | 0 |
| B21 | 309 | 0.77 | 13.569 | 0.248 | 0.248 | 0 | Dec. | L | L-M | 1 | 1 |
| B22 | 20 | 0.87 | 24.669 | 0.098 | 0.097 | 0 | Cst. | Mix. | Mix. | 1 | 0 |
| B23 | 25 | 0.76 | 9.365 | 0.236 | 0.236 | 0 | Saturat. | L | L-M | 0 | 0 |
| B24 | 10 | 0.91 | 6.014 | 0.257 | 0.257 | 0 | Cst. | L | L-M | 1 | 0 |
| B25 | 12 | 0.5 | 10.537 | 0.205 | 0.205 | 0 | Cst. | M | L-M | 0 | 0 |
| B26 | 12 | 0.86 | 5.382 | 0.318 | 0.318 | 0 | Cst. | Mix. | L-M | 1 | 0 |
| B27 | 8 | 0.29 | 12.172 | 0.055 | 0.617 | 0.562 | Cst. | Mix. | L-M | 0 | 0 |
| B28 | 44 | 0.75 | 20.118 | 0.175 | 0.217 | 0.041 | Cst. | Mix. | L-M | 1 | 1 |
| B29 | 10 | 0.27 | 42.091 | 0.049 | 0.125 | 0.076 | Cst. | L | L-M | 1 | 1 |
| B30 | 9 | 1 | 9.646 | 0.151 | 0.151 | 0 | Cst. | Mix. | L-M | 1 | 0 |
| B31 | 12 | 0.71 | 11.065 | 0.197 | 0.196 | 0 | Cst. | Mix. | Mix. | 1 | 0 |
| B32 | 13 | 0.76 | 20.683 | 0.097 | 0.096 | 0 | Cst. | Mix. | L-M | 0 | 0 |
| S1 | 76 | 0.494 | 34.354 | 0.096 | 0.096 | 0 | Saturat. | Mix. | L-M | 2 | 0 |
| S2 | 15 | 0.385 | 26.032 | 0.091 | 0.091 | 0 | Saturat. | Mix. | L-M | 1 | 0 |
| S3 | 19 | 0.238 | 21.777 | 0.146 | 0.277 | 0.131 | Cst. | Mix. | L-M | 0 | 0 |
| S4 | 13 | 0.619 | 21.136 | 0.099 | 0.099 | 0 | Cst. | M | L-M | 1 | 3 |
| S5 | 43 | 0.606 | 21.409 | 0.162 | 0.162 | 0 | Saturat. | Mix. | L-M | 1 | 0 |
| S6 | 15 | 0.469 | 44.837 | 0.057 | 0.057 | 0 | Cst. | L | L-M | 0 | 0 |
| S7 | 9 | 0.257 | 80.541 | -0.011 | 0.234 | 0.244 | Cst. | Mix. | L-M | 0 | 0 |
| S8 | 29 | 0.492 | 43.744 | 0.064 | 0.11 | 0.045 | Dec. | L | L-M | 5 | 7 |
| S9 | 214 | 0.491 | 83.402 | 0.076 | 0.075 | 0 | Saturat. | Mix. | L-M | 0 | 0 |
| S10 | 119 | 0.386 | 71.135 | 0.101 | 0.268 | 0.168 | Expo. | M-H | M-H | 2 | 4 |
| S11 | 11 | 0.333 | 42.192 | 0.056 | 0.056 | 0 | Saturat. | Mix. | L-M | 2 | 4 |
| S12 | 12 | 0.444 | 81.207 | 0.033 | 0.033 | 0 | Expo. | L | L-M | 1 | 1 |
| S13 | 44 | 0.431 | 19.786 | 0.166 | 0.166 | 0 | Saturat. | Mix. | Mix. | 0 | 0 |
| S14 | 22 | 0.759 | 34.556 | 0.060 | 0.119 | 0.058 | Cst. | L | L-M | 1 | 3 |
| S15 | 78 | 0.574 | 88.47 | 0.053 | 0.053 | 0 | Expo. | Mix. | L-M | 1 | 1 |
| S16 | 18 | 0.419 | 26.471 | 0.097 | 0.107 | 0.010 | Cst. | Mix. | L-M | 1 | 3 |
| S17 | 37 | 0.389 | 48.842 | 0.056 | 0.056 | 0 | Saturat. | L | L-M | 0 | 0 |
| S18 | 144 | 0.427 | 86.266 | 0.060 | 0.060 | 0 | Expo. | Mix. | Mix. | 0 | 0 |
| S19 | 20 | 0.333 | 25.219 | 0.121 | 0.121 | 0 | Dec. | Mix. | L-M | 0 | 0 |
| S20 | 16 | 0.842 | 36.326 | 0.049 | 0.049 | 0 | Saturat. | M | L-M | 1 | 3 |
| S21 | 20 | 0.308 | 66.909 | 0.058 | 0.058 | 0 | Cst. | L | L-M | 0 | 0 |
| S22 | 69 | 0.406 | 70.885 | 0.52 | 0.52 | 0 | Saturat. | Mix. | L-M | 0 | 0 |
| S23 | 60 | 0.488 | 34.422 | 0.110 | 0.110 | 0 | Saturat. | Mix. | Mix. | 0 | 0 |
| S24 | 45 | 0.144 | 29.252 | 0.152 | 0.152 | 0 | Saturat. | Mix. | L-M | 1 | 0 |
| A1 | 118 | 0.929 | 67.184 | 0.057 | 0.057 | 0 | Expo. | Mix. | L-M | 1 | 1 |
| A2 | 136 | 1 | 67.352 | 0.063 | 0.063 | 0 | Saturat. | Mix. | Mix. | 1 | 1 |
| A3 | 86 | 0.789 | 80.689 | 0.044 | 0.044 | 0 | Cst. | Mix. | L-M | 1 | 1 |
| A4 | 170 | 0.783 | 72.077 | 0.067 | 0.067 | 0 | Expo. | Mix. | Mix. | 1 | 0 |
| A5 | 61 | 0.466 | 69.867 | 0.56 | 0.56 | 0 | Expo. | L | L-M | 5 | 7 |
| A6 | 295 | 0.472 | 68.163 | 0.079 | 0.079 | 0 | Expo. | Mix. | Mix. | 1 | 1 |
| A7 | 50 | 0.794 | 54.784 | 0.055 | 0.055 | 0 | Saturat. | Mix. | L-M | 0 | 0 |
| A8 | 117 | 0.639 | 69.933 | 0.058 | 0.087 | 0.029 | Cst. | Mix. | L-M | 1 | 0 |
| A9 | 147 | 0.583 | 72.804 | 0.069 | 0.069 | 0 | Cst. | Mix. | L-M | 1 | 0 |
| A10 | 60 | 0.706 | 43.327 | 0.077 | 0.077 | 0 | Dec. | Mix. | L-M | 1 | 1 |
| A11 | 127 | 0.49 | 87.368 | 0.026 | 0.168 | 0.142 | Dec. | Mix. | L-M | 2 | 0 |
| A12 | 186 | 0.882 | 78.066 | 0.057 | 0.057 | 0 | Expo. | Mix. | L-M | 0 | 0 |
| A13 | 128 | 0.81 | 33.396 | 0.104 | 0.104 | 0 | Saturat. | Mix. | L-M | 1 | 1 |
| A14 | 54 | 0.454 | 53.951 | 0.029 | 0.429 | 0.399 | Cst. | Mix. | Mix. | 1 | 1 |
| A15 | 110 | 0.701 | 33.893 | 0.103 | 0.161 | 0.058 | Cst. | Mix. | Mix. | 0 | 0 |
| A16 | 159 | 0.518 | 73.052 | 0.072 | 0.072 | 0 | Expo. | Mix. | L-M | 1 | 3 |
