# Supplementary material for: Diversification dynamics in the Neotropics through time, clades, and biogeographic regions
Source: eLife. 2022 Oct 27;11:e74503. doi: 10.7554/eLife.74503 (PMC9668338; doi:10.7554/eLife.74503)
Supplement: Figure 6—source data 4. [file elife-74503-fig6-data4.docx]

**Figure 6 - Source data 4.**

Source data for subfigure (e) and (g). Tree size, sampling fraction, crown age, pulled diversification rates (*r_p_*) (based on the constant model), present-day pulled extinction (*µ_p_(0)*) and species richness dynamics (constant vs. declining speciation; based on the most supported model). In addition, for each clade, we provide the main elevational range (and an alternative codification [Elev. 2] if the lowland and montane categories are pooled together; see text) and its assignation to 5 or 7 biogeographic clusters, respectively. Abbreviations: Lowland = L.; Montane = M., Highland = H.; Constant = Cst.; Declining = Decline

|  | Tree size | Sampling | Crown age | r_p_ | *µ_p_(0)* | Richness | Elevation | Elev. 2 | 5 cluster | 7 cluster |
| --- | --- | --- | --- | --- | --- | --- | --- | --- | --- | --- |
| P1 | 13 | 0.116 | 2.88 | 1.831 | -20.436 | decline | Mixed | L-M | 1 | 0 |
| P2 | 38 | 0.279 | 0.936 | 5.626 | -19.701 | decline | Mixed | L-M | 1 | 3 |
| P3 | 12 | 0.158 | 43.022 | 0.086 | -0.516 | constant | Mixed | L-M | 1 | 1 |
| P4 | 10 | 0.125 | 13.8 | 0.215 | -1.915 | constant | Mixed | L-M | 1 | 0 |
| P5 | 10 | 0.125 | 7.32 | 0.45 | -1.612 | constant | Mixed | L-M | 1 | 1 |
| P6 | 14 | 0.452 | 5.818 | 0.668 | -3.897 | constant | Mixed | L-M | 1 | 1 |
| P7 | 29 | 0.569 | 12.272 | 0.339 | -0.375 | constant | Mixed | L-M | 1 | 1 |
| P8 | 15 | 0.139 | 11.88 | 0.234 | -1.656 | constant | M | L-M | 1 | 1 |
| P9 | 10 | 0.153 | 19.626 | 0.229 | -0.552 | constant | Mixed | L-M | 1 | 1 |
| P10 | 24 | 0.407 | 25.524 | 0.174 | -0.415 | constant | Mixed | L-M | 1 | 1 |
| P12 | 15 | 0.349 | 13.044 | 0.273 | -0.767 | constant | Mixed | L-M | 1 | 1 |
| P14 | 10 | 0.2 | 26.003 | 0.101 | -0.487 | constant | Mixed | L-M | 1 | 1 |
| P15 | 19 | 0.63 | 19.876 | 0.167 | -0.497 | constant | L | L-M | 1 | 1 |
| P16 | 19 | 0.207 | 7.085 | 0.532 | -0.941 | constant | L | L-M | 1 | 1 |
| P17 | 18 | 0.182 | 22.422 | 0.151 | -0.027 | constant | L | L-M | 1 | 1 |
| P18 | 7 | 0.875 | 10.802 | 0.46 | -5.229 | constant | L | L-M | 1 | 1 |
| P19 | 22 | 0.18 | 28.016 | 0.182 | -0.609 | constant | Mixed | L-M | 1 | 1 |
| P20 | 29 | 0.725 | 20.251 | 0.179 | -0.072 | constant | M | L-M | 1 | 0 |
| P22 | 19 | 0.432 | 17.636 | 0.267 | -0.66 | constant | Mixed | L-M | 1 | 1 |
| P23 | 9 | 0.562 | 25.054 | 0.112 | -0.2 | constant | Mixed | L-M | 1 | 1 |
| P24 | 200 | 0.364 | 17.027 | 0.35 | -0.496 | decline | L | L-M | 1 | 1 |
| P25 | 24 | 0.889 | 19.209 | 0.221 | -0.971 | constant | M-H | M-H | 1 | 3 |
| P26 | 43 | 0.606 | 14.632 | 0.305 | -0.558 | constant | L | L-M | 1 | 0 |
| P27 | 19 | 0.543 | 4.666 | 0.693 | -0.436 | constant | Mixed | Mixed | 2 | 2 |
| P28 | 37 | 0.285 | 2.755 | 2.102 | -8.957 | decline | M | L-M | 1 | 1 |
| P29 | 13 | 0.933 | 15.285 | 0.32 | -3.156 | decline | Mixed | L-M | 1 | 0 |
| P30 | 14 | 0.875 | 37.321 | 0.061 | -0.264 | constant | Mixed | L-M | 0 | 0 |
| P31 | 78 | 0.975 | 30.969 | 0.115 | -0.247 | decline | M-H | M-H | 5 | 7 |
| P32 | 22 | 0.786 | 20.511 | 0.204 | -0.603 | constant | L | L-M | 5 | 7 |
| P35 | 114 | 0.991 | 38.071 | 0.184 | -0.311 | decline | L | L-M | 1 | 3 |
| P36 | 154 | 0.987 | 27.09 | 0.2 | -0.237 | constant | Mixed | L-M | 0 | 0 |
| P37 | 178 | 0.989 | 43.622 | 0.186 | -0.356 | decline | L | L-M | 1 | 1 |
| P38 | 102 | 1 | 26.923 | 0.214 | -0.492 | decline | Mixed | L-M | 1 | 1 |
| P39 | 22 | 1 | 34.208 | 0.043 | -0.113 | constant | Mixed | L-M | 1 | 1 |
| P40 | 271 | 0.456 | 20.391 | 0.288 | -0.163 | decline | Mixed | L-M | 0 | 0 |
| P42 | 789 | 0.213 | 31.481 | 0.243 | -0.363 | decline | Mixed | L-M | 1 | 0 |
| P43 | 168 | 0.247 | 21.008 | 0.294 | -0.317 | constant | Mixed | L-M | 0 | 0 |
| P44 | 126 | 0.331 | 6 | 1.254 | -4.486 | decline | Mixed | L-M | 1 | 1 |
| P45 | 588 | 0.49 | 48.448 | 0.196 | -0.458 | decline | Mixed | L-M | 1 | 1 |
| P46 | 111 | 0.793 | 28.918 | 0.241 | -1.109 | decline | Mixed | L-M | 1 | 1 |
| P47 | 21 | 1 | 28.86 | 0.034 | -0.046 | decline | M-H | M-H | 2 | 0 |
| P50 | 63 | 0.708 | 7.206 | 0.649 | -1.089 | constant | Mixed | Mixed | 0 | 0 |
| P51 | 189 | 0.442 | 21.785 | 0.206 | -0.431 | decline | Mixed | L-M | 1 | 0 |
| P52 | 495 | 0.319 | 20.793 | 0.348 | -0.591 | decline | Mixed | L-M | 0 | 0 |
| P54 | 38 | 0.559 | 45.013 | 0.07 | -0.207 | constant | M | L-M | 2 | 0 |
| P55 | 120 | 0.458 | 19.5 | 0.176 | -0.18 | decline | L | L-M | 1 | 0 |
| P56 | 150 | 0.251 | 27.879 | 0.203 | -0.781 | decline | Mixed | L-M | 2 | 2 |
| P57 | 161 | 0.25 | 48.279 | 0.12 | -0.239 | decline | Mixed | L-M | 0 | 0 |
| P58 | 307 | 0.24 | 42.379 | 0.125 | -0.248 | decline | M-H | M-H | 0 | 0 |
| P59 | 34 | 0.351 | 24.169 | 0.169 | -0.864 | decline | L | L-M | 1 | 1 |
| P60 | 16 | 0.182 | 35.745 | 0.101 | -0.141 | constant | Mixed | L-M | 1 | 0 |
| P61 | 14 | 0.127 | 8.684 | 0.499 | -3.382 | constant | Mixed | Mixed | 0 | 0 |
| P62 | 10 | 0.4 | 1.647 | 2.454 | -27.409 | decline | L | L-M | 1 | 0 |
| P63 | 10 | 0.417 | 0.878 | 4.252 | -48.822 | decline | Mixed | L-M | 1 | 0 |
| P64 | 10 | 0.588 | 4.566 | 0.668 | -5.51 | decline | L | L-M | 2 | 0 |
| P65 | 32 | 0.107 | 21.506 | 0.175 | -0.037 | constant | Mixed | L-M | 1 | 0 |
| P66 | 48 | 0.276 | 0.497 | 9.669 | -20.667 | constant | L | L-M | 0 | 0 |
| M2 | 192 | 0.86 | 41.856 | 0.113 | -0.234 | decline | L | L-M | 1 | 1 |
| M3 | 20 | 0.69 | 22.502 | 0.191 | -0.991 | decline | L | L-M | 1 | 0 |
| M4 | 95 | 0.477 | 21.539 | 0.113 | -0.117 | decline | L | L-M | 1 | 1 |
| M5 | 43 | 0.417 | 25.493 | 0.144 | -0.404 | constant | Mixed | L-M | 1 | 0 |
| M6 | 279 | 0.676 | 12.657 | 0.591 | -0.915 | decline | Mixed | Mixed | 0 | 0 |
| M7 | 11 | 0.688 | 10.6 | 0.405 | -3.735 | constant | L | L-M | 1 | 0 |
| M8 | 10 | 0.455 | 13.809 | 0.125 | -0.265 | constant | L | L-M | 1 | 3 |
| M9 | 20 | 0.909 | 34.902 | 0.145 | -1.789 | decline | Mixed | L-M | 1 | 1 |
| M11 | 10 | 1 | 16 | 0.135 | -1.684 | constant | Mixed | Mixed | 0 | 0 |
| M12 | 199 | 0.816 | 35.28 | 0.135 | -0.16 | decline | L | L-M | 0 | 0 |
| B1 | 39 | 0.71 | 11.823 | 0.404 | -2.073 | decline | L | L-M | 1 | 1 |
| B2 | 233 | 0.69 | 26.097 | 0.216 | -0.275 | constant | Mixed | L-M | 1 | 1 |
| B3 | 8 | 0.73 | 24.786 | 0.033 | -0.012 | constant | Mixed | Mixed | 0 | 0 |
| B4 | 118 | 0.71 | 28.953 | 0.174 | -0.367 | decline | Mixed | L-M | 0 | 0 |
| B5 | 55 | 0.49 | 25.166 | 0.24 | -0.565 | constant | Mixed | Mixed | 1 | 1 |
| B6 | 9 | 0.6 | 23.421 | 0.153 | -1.316 | constant | Mixed | L-M | 0 | 0 |
| B7 | 7 | 0.64 | 18.548 | 0.105 | -0.292 | constant | M | L-M | 1 | 1 |
| B8 | 165 | 0.71 | 16.825 | 0.389 | -0.671 | constant | L | L-M | 1 | 1 |
| B9 | 292 | 0.94 | 32.586 | 0.188 | -0.411 | decline | L | L-M | 1 | 0 |
| B10 | 316 | 0.79 | 25.501 | 0.217 | -0.266 | constant | Mixed | Mixed | 1 | 0 |
| B11 | 80 | 0.68 | 23.515 | 0.195 | -0.128 | constant | Mixed | Mixed | 1 | 1 |
| B12 | 19 | 0.37 | 20.151 | 0.18 | -0.633 | constant | L | L-M | 1 | 0 |
| B13 | 36 | 0.95 | 17.382 | 0.288 | -1.342 | decline | Mixed | L-M | 1 | 0 |
| B14 | 39 | 0.85 | 8.199 | 0.704 | -1.313 | constant | Mixed | L-M | 1 | 0 |
| B15 | 17 | 0.94 | 13.105 | 0.323 | -0.459 | constant | Mixed | Mixed | 1 | 0 |
| B16 | 67 | 0.62 | 24.509 | 0.297 | -1.256 | decline | Mixed | L-M | 1 | 0 |
| B17 | 10 | 0.37 | 13.101 | 0.353 | -2.502 | constant | L | L-M | 0 | 0 |
| B18 | 89 | 0.99 | 11.208 | 0.508 | -0.568 | constant | L | L-M | 1 | 0 |
| B19 | 92 | 0.89 | 12.021 | 0.526 | -0.976 | constant | Mixed | L-M | 0 | 0 |
| B20 | 41 | 0.82 | 18.038 | 0.328 | -1.419 | decline | Mixed | L-M | 1 | 0 |
| B21 | 309 | 0.77 | 13.569 | 0.375 | -0.041 | decline | Mixed | L-M | 1 | 1 |
| B23 | 25 | 0.76 | 9.365 | 0.56 | -0.996 | constant | L | L-M | 0 | 0 |
| B24 | 10 | 0.91 | 6.014 | 0.633 | -2.426 | constant | Mixed | Mixed | 1 | 0 |
| B25 | 12 | 0.5 | 10.537 | 0.533 | -3.106 | constant | L | L-M | 0 | 0 |
| B26 | 12 | 0.86 | 5.382 | 0.847 | -0.481 | constant | L | L-M | 1 | 0 |
| B27 | 8 | 0.29 | 12.172 | 0.171 | -0.54 | constant | M | L-M | 0 | 0 |
| B28 | 44 | 0.75 | 20.118 | 0.193 | -0.796 | decline | Mixed | L-M | 1 | 1 |
| B29 | 10 | 0.27 | 42.091 | 0.072 | -0.351 | constant | Mixed | L-M | 1 | 1 |
| B31 | 12 | 0.71 | 11.065 | 0.282 | -1.09 | constant | Mixed | L-M | 1 | 0 |
| B32 | 13 | 0.76 | 20.683 | 0.16 | -0.205 | constant | L | L-M | 0 | 0 |
| S1 | 76 | 0.494 | 34.354 | 0.152 | -0.727 | decline | Mixed | L-M | 2 | 0 |
| S2 | 15 | 0.385 | 26.032 | 0.179 | -0.578 | constant | Mixed | Mixed | 1 | 0 |
| S3 | 19 | 0.238 | 21.777 | 0.169 | -0.448 | constant | Mixed | L-M | 0 | 0 |
| S4 | 13 | 0.619 | 21.136 | 0.19 | -1.21 | constant | Mixed | L-M | 1 | 3 |
| S5 | 43 | 0.606 | 21.409 | 0.266 | -0.533 | constant | Mixed | L-M | 1 | 0 |
| S6 | 15 | 0.469 | 44.837 | 0.12 | -0.046 | constant | Mixed | L-M | 0 | 0 |
| S7 | 9 | 0.257 | 80.541 | 0.015 | -0.122 | constant | M | L-M | 0 | 0 |
| S9 | 214 | 0.491 | 83.402 | 0.12 | -0.153 | constant | Mixed | L-M | 0 | 0 |
| S10 | 119 | 0.386 | 71.135 | 0.105 | -0.258 | decline | L | L-M | 2 | 4 |
| S11 | 11 | 0.333 | 42.192 | 0.173 | -1.878 | constant | Mixed | L-M | 2 | 4 |
| S12 | 12 | 0.444 | 81.207 | 0.079 | -0.375 | constant | L | L-M | 1 | 1 |
| S13 | 44 | 0.431 | 19.786 | 0.248 | -0.477 | constant | Mixed | L-M | 0 | 0 |
| S15 | 78 | 0.574 | 88.47 | 0.081 | -0.194 | decline | M-H | M-H | 1 | 1 |
| S16 | 18 | 0.419 | 26.471 | 0.113 | -0.07 | decline | Mixed | L-M | 1 | 3 |
| S17 | 37 | 0.389 | 48.842 | 0.116 | -0.349 | constant | L | L-M | 0 | 0 |
| S18 | 144 | 0.427 | 86.266 | 0.074 | -0.163 | decline | Mixed | Mixed | 0 | 0 |
| S19 | 20 | 0.333 | 25.219 | 0.228 | -0.027 | constant | L | L-M | 0 | 0 |
| S20 | 16 | 0.842 | 36.326 | 0.148 | -0.443 | constant | Mixed | L-M | 1 | 3 |
| S21 | 20 | 0.308 | 66.909 | 0.07 | -0.197 | constant | Mixed | L-M | 0 | 0 |
| S22 | 69 | 0.406 | 70.885 | 0.086 | -0.061 | constant | L | L-M | 0 | 0 |
| S23 | 60 | 0.488 | 34.422 | 0.158 | -0.21 | constant | Mixed | Mixed | 0 | 0 |
| S24 | 45 | 0.144 | 29.252 | 0.215 | -0.172 | constant | Mixed | L-M | 1 | 0 |
| A1 | 118 | 0.929 | 67.184 | 0.084 | -0.067 | constant | M | L-M | 1 | 1 |
| A2 | 136 | 1 | 67.352 | 0.087 | -0.221 | decline | L | L-M | 1 | 1 |
| A3 | 86 | 0.789 | 80.689 | 0.06 | -0.12 | constant | Mixed | L-M | 1 | 1 |
| A4 | 170 | 0.783 | 72.077 | 0.124 | -0.35 | decline | Mixed | Mixed | 1 | 0 |
| A5 | 61 | 0.466 | 69.867 | 0.09 | -0.226 | constant | Mixed | L-M | 5 | 7 |
| A6 | 295 | 0.472 | 68.163 | 0.109 | -0.139 | constant | Mixed | L-M | 1 | 1 |
| A7 | 50 | 0.794 | 54.784 | 0.086 | -0.047 | constant | Mixed | Mixed | 0 | 0 |
| A8 | 117 | 0.639 | 69.933 | 0.061 | -0.071 | constant | Mixed | L-M | 1 | 0 |
| A9 | 147 | 0.583 | 72.804 | 0.083 | -0.112 | constant | Mixed | Mixed | 1 | 0 |
| A10 | 60 | 0.706 | 43.327 | 0.106 | -0.179 | constant | L | L-M | 1 | 1 |
| A12 | 186 | 0.882 | 78.066 | 0.078 | -0.142 | decline | Mixed | Mixed | 0 | 0 |
| A13 | 128 | 0.81 | 33.396 | 0.158 | -0.18 | decline | Mixed | L-M | 1 | 1 |
| A14 | 54 | 0.454 | 53.951 | 0.039 | -0.072 | decline | Mixed | L-M | 1 | 1 |
| A15 | 110 | 0.701 | 33.893 | 0.107 | -0.116 | decline | Mixed | L-M | 0 | 0 |
| A16 | 159 | 0.518 | 73.052 | 0.092 | -0.259 | decline | Mixed | L-M | 1 | 3 |
