# Supplementary material for: Diversification dynamics in the Neotropics through time, clades, and biogeographic regions
Source: eLife. 2022 Oct 27;11:e74503. doi: 10.7554/eLife.74503 (PMC9668338; doi:10.7554/eLife.74503)
Supplement: Supplementary file 1. — More details are given on each table. [file elife-74503-supp1.docx]

**Supplementary file 1**

This file contains the complete results of model selection based on **(A)** traditional diversification analyses comparing constant and time-dependent models; **(B)** traditional diversification analyses comparing constant, time-, temperature- and Andean uplift-dependent models; and **(C)** results for the pulled diversification rate analyses. More details are given on each table.

**Supplementary file A.**

Summary results showing the best-fit model (among constant and time-dependent models) for each clade and the derived species richness pattern (gradual increase [Sc. 1], exponential increase [Sc. 2], saturated increase [Sc. 3] and decline [Sc. 4]). For each model category, we fitted three models in which speciation (Birth “B”) and/or extinction (Death “D”) remain constant (“cst”), or vary (“Var.”) continuously with time. The value of speciation (lambda) and extinction (mu) rates is provided, as well as the value of the dependence between speciation and extinction rates with time (alpha and beta values, respectively). For time-dependent models, *α* and *β > 0* reflect decreasing speciation and extinction towards the present, respectively, while *α* and *β <0* indicate the opposite, increasing speciation and extinction towards the present. Species richness patterns are derived from the diversification trend through time. For each phylogeny, the sampling fraction (*i.e.* number of species sampled from the total described), crown age (in million years ago), and number of species (#spp) is provided together with the main elevational range (lowland, montane, highland) of the species in the clade. Mixed patterns could be observed in this category. Abbreviations for model type; B = Birth; D = Death; cst = constant; Var. = variable.

| Taxa | Clade | # spp. | Sampling fraction | Crown age | Elevation | Model type | Lambda | Alpha | Mu | Beta | Richness pattern | |  |  |  |
| --- | --- | --- | --- | --- | --- | --- | --- | --- | --- | --- | --- | --- | --- | --- | --- |
| Plants | P1 | 13 | 0.12 | 2.88 | Lowland-Montane | BcstDTimeVar | 2.08 | NA | 0.02 | 2.52 | Exponential | |  |  |  |
| Plants | P2 | 38 | 0.28 | 0.94 | Lowland-Montane | BcstDTimeVar | 6.43 | NA | 0.15 | 5.64 | Exponential | |  |  |  |
| Plants | P3 | 12 | 0.16 | 43.02 | Lowland-Montane | BcstDcst | 0.15 | NA | 0.09 | NA | Gradual | |  |  |  |
| Plants | P4 | 10 | 0.12 | 13.8 | Lowland-Montane | BcstDcst | 1.02 | NA | 0.89 | NA | Gradual | |  |  |  |
| Plants | P5 | 10 | 0.12 | 7.32 | Lowland-Montane | BcstDcst | 1.35 | NA | 1.04 | NA | Gradual | |  |  |  |
| Plants | P6 | 14 | 0.45 | 5.82 | Lowland-Montane | BcstDcst | 0.47 | NA | 0 | NA | Gradual | |  |  |  |
| Plants | P7 | 29 | 0.57 | 12.27 | Lowland-Montane | BcstDcst | 0.29 | NA | 0 | NA | Gradual | |  |  |  |
| Plants | P8 | 15 | 0.14 | 11.88 | Lowland-Montane | BTimeVarDcst | 1.12 | -0.27 | 0 | NA | Exponential | |  |  |  |
| Plants | P9 | 10 | 0.15 | 19.63 | Lowland-Montane | BcstDcst | 0.18 | NA | 0 | NA | Gradual | |  |  |  |
| Plants | P10 | 24 | 0.41 | 25.52 | Lowland-Montane | BcstDcst | 0.14 | NA | 0 | NA | Gradual | |  |  |  |
| Plants | P11 | 42 | 0.68 | 15.15 | Lowland-Montane | BcstDcst | 0.43 | NA | 0.24 | NA | Gradual | |  |  |  |
| Plants | P12 | 15 | 0.35 | 13.04 | Lowland-Montane | BcstDcst | 0.3 | NA | 0.08 | NA | Gradual | |  |  |  |
| Plants | P13 | 46 | 0.32 | 56.31 | Lowland-Montane | BTimeVarDcst | 0.24 | 0.01 | 0.29 | NA | Declining | |  |  |  |
| Plants | P14 | 10 | 0.2 | 26 | Lowland-Montane | BcstDcst | 0.43 | NA | 0.38 | NA | Gradual | |  |  |  |
| Plants | P15 | 19 | 0.63 | 19.88 | Lowland-Montane | BcstDcst | 0.19 | NA | 0.05 | NA | Gradual | |  |  |  |
| Plants | P16 | 19 | 0.21 | 7.08 | Lowland-Montane | BcstDcst | 1.14 | NA | 0.69 | NA | Gradual | |  |  |  |
| Plants | P17 | 18 | 0.18 | 22.42 | Lowland-Montane | BcstDcst | 0.27 | NA | 0.14 | NA | Gradual | |  |  |  |
| Plants | P18 | 7 | 0.88 | 10.8 | Lowland-Montane | BcstDcst | 0.11 | NA | 0 | NA | Gradual | |  |  |  |
| Plants | P19 | 22 | 0.18 | 28.02 | Lowland-Montane | BcstDTimeVar | 0.21 | NA | 0 | 0.39 | Exponential | |  |  |  |
| Plants | P20 | 29 | 0.72 | 20.25 | Lowland-Montane | BcstDcst | 0.14 | NA | 0 | NA | Gradual | |  |  |  |
| Plants | P21 | 103 | 0.41 | 20.77 | Lowland-Montane | BTimeVarDcst | 0.09 | 0.11 | 0 | NA | Saturated | |  |  |  |
| Plants | P22 | 19 | 0.43 | 17.64 | Montane-Highland | BcstDcst | 0.17 | NA | 0 | NA | Gradual | |  |  |  |
| Plants | P23 | 9 | 0.56 | 25.05 | Lowland-Montane | BcstDcst | 0.13 | NA | 0.07 | NA | Gradual | |  |  |  |
| Plants | P24 | 200 | 0.36 | 17.03 | Mixed | BTimeVarDcst | 1.64 | -0.29 | 0 | NA | Exponential | |  |  |  |
| Plants | P25 | 24 | 0.89 | 19.21 | Lowland-Montane | BcstDcst | 0.14 | NA | 0 | NA | Gradual | |  |  |  |
| Plants | P26 | 43 | 0.61 | 14.63 | Lowland-Montane | BcstDcst | 0.23 | NA | 0 | NA | Gradual | |  |  |  |
| Plants | P27 | 19 | 0.54 | 4.67 | Lowland-Montane | BcstDcst | 0.74 | NA | 0.17 | NA | Gradual | |  |  |  |
| Plants | P28 | 37 | 0.28 | 2.75 | Montane-Highland | BcstDTimeVar | 2.18 | NA | 0 | 4.34 | Exponential | |  |  |  |
| Plants | P29 | 13 | 0.93 | 15.29 | Lowland-Montane | BcstDcst | 0.11 | NA | 0 | NA | Gradual | |  |  |  |
| Plants | P30 | 14 | 0.88 | 37.32 | Lowland-Montane | BcstDcst | 0.1 | NA | 0.06 | NA | Gradual | |  |  |  |
| Plants | P31 | 78 | 0.98 | 30.97 | Lowland-Montane | BcstDcst | 0.19 | NA | 0.08 | NA | Gradual | |  |  |  |
| Plants | P32 | 22 | 0.79 | 20.51 | Lowland-Montane | BcstDcst | 0.14 | NA | 0 | NA | Gradual | |  |  |  |
| Plants | P33 | 16 | 1 | 19.96 | Lowland-Montane | BcstDcst | 0.09 | NA | 0 | NA | Gradual | |  |  |  |
| Plants | P34 | 32 | 1 | 29.68 | Lowland-Montane | BcstDcst | 0.21 | NA | 0.14 | NA | Gradual | |  |  |  |
| Plants | P35 | 114 | 0.99 | 38.07 | Lowland-Montane | BcstDcst | 0.13 | NA | 0 | NA | Gradual | |  |  |  |
| Plants | P36 | 154 | 0.99 | 27.09 | Lowland-Montane | BcstDcst | 0.2 | NA | 0 | NA | Gradual | |  |  |  |
| Plants | P37 | 178 | 0.99 | 43.62 | Lowland-Montane | BcstDTimeVar | 0.19 | NA | 0 | 0.14 | Exponential | |  |  |  |
| Plants | P38 | 102 | 1 | 26.92 | Lowland-Montane | BcstDTimeVar | 0.25 | NA | 0.01 | 0.18 | Exponential | |  |  |  |
| Plants | P39 | 22 | 1 | 34.21 | Lowland-Montane | BcstDcst | 0.26 | NA | 0.26 | NA | Gradual | |  |  |  |
| Plants | P40 | 271 | 0.46 | 20.39 | Lowland-Montane | BTimeVarDcst | 0.76 | -0.05 | 0.25 | NA | Exponential | |  |  |  |
| Plants | P41 | 670 | 0.13 | 17.87 | Montane-Highland | BcstDcst | 3.08 | NA | 2.76 | NA | Gradual | |  |  |  |
| Plants | P42 | 789 | 0.21 | 31.48 | Mixed | BTimeVarDcst | 0.75 | -0.02 | 0.41 | NA | Exponential | |  |  |  |
| Plants | P43 | 168 | 0.25 | 21.01 | Lowland-Montane | BcstDTimeVar | 0.71 | NA | 0.31 | 0.05 | Exponential | |  |  |  |
| Plants | P44 | 126 | 0.33 | 6 | Lowland-Montane | BTimeVarDcst | 3.76 | -0.68 | 0 | NA | Exponential | |  |  |  |
| Plants | P45 | 588 | 0.49 | 48.45 | Lowland-Montane | BTimeVarDTimeVar | 0.16 | 0.01 | 0.01 | 0.09 | Exponential | |  |  |  |
| Plants | P46 | 111 | 0.79 | 28.92 | Lowland-Montane | BcstDcst | 0.16 | NA | 0 | NA | Gradual | |  |  |  |
| Plants | P47 | 21 | 1 | 28.86 | Lowland-Montane | BcstDcst | 0.34 | NA | 0.34 | NA | Gradual | |  |  |  |
| Plants | P48 | 50 | 0.23 | 32.21 | Lowland-Montane | BcstDcst | 3.13 | NA | 3.13 | NA | Gradual | |  |  |  |
| Plants | P49 | 44 | 0.3 | 30.99 | Montane-Highland | BcstDcst | 1.18 | NA | 1.14 | NA | Gradual | |  |  |  |
| Plants | P50 | 63 | 0.71 | 7.21 | Lowland-Montane | BcstDcst | 0.59 | NA | 0 | NA | Gradual | |  |  |  |
| Plants | P51 | 189 | 0.44 | 21.79 | Lowland-Montane | BcstDcst | 0.59 | NA | 0.38 | NA | Gradual | |  |  |  |
| Plants | P52 | 495 | 0.32 | 20.79 | Mixed | BTimeVarDcst | 0.59 | -0.07 | 0 | NA | Exponential | |  |  |  |
| Plants | P53 | 86 | 0.77 | 30.28 | Lowland-Montane | BcstDcst | 0.15 | NA | 0 | NA | Gradual | |  |  |  |
| Plants | P54 | 38 | 0.56 | 45.01 | Lowland-Montane | BcstDcst | 0.19 | NA | 0.13 | NA | Gradual | |  |  |  |
| Plants | P55 | 120 | 0.46 | 19.5 | Lowland-Montane | BTimeVarDcst | 1.01 | -0.21 | 0 | NA | Exponential | |  |  |  |
| Plants | P56 | 150 | 0.25 | 27.88 | Lowland-Montane | BcstDcst | 0.25 | NA | 0.05 | NA | Gradual | |  |  |  |
| Plants | P57 | 161 | 0.25 | 48.28 | Lowland-Montane | BcstDcst | 0.18 | NA | 0.07 | NA | Gradual | |  |  |  |
| Plants | P58 | 307 | 0.24 | 42.38 | Lowland-Montane | BcstDcst | 0.53 | NA | 0.4 | NA | Gradual | |  |  |  |
| Plants | P59 | 34 | 0.35 | 24.17 | Lowland-Montane | BcstDTimeVar | 0.43 | NA | 0.08 | 0.15 | Exponential | |  |  |  |
| Plants | P60 | 16 | 0.18 | 35.74 | Lowland-Montane | BcstDTimeVar | 0.17 | NA | 0 | 0.25 | Exponential | |  |  |  |
| Plants | P61 | 14 | 0.13 | 8.68 | Lowland-Montane | BcstDcst | 0.51 | NA | 0.07 | NA | Gradual | |  |  |  |
| Plants | P62 | 10 | 0.4 | 1.65 | Lowland-Montane | BcstDcst | 1.7 | NA | 0 | NA | Gradual | |  |  |  |
| Plants | P63 | 10 | 0.42 | 0.88 | Lowland-Montane | BcstDcst | 2.23 | NA | 0 | NA | Gradual | |  |  |  |
| Plants | P64 | 10 | 0.59 | 4.57 | Lowland-Montane | BcstDcst | 0.42 | NA | 0 | NA | Gradual | |  |  |  |
| Plants | P65 | 32 | 0.11 | 21.51 | Montane-Highland | BcstDcst | 0.71 | NA | 0.55 | NA | Gradual | |  |  |  |
| Plants | P66 | 48 | 0.28 | 0.5 | Lowland-Montane | BcstDcst | 8.82 | NA | 0 | NA | Gradual | |  |  |  |
| Mammals | M1 | 32 | 1 | 67.96 | Lowland-Montane | BcstDcst | 0.08 | NA | 0.05 | NA | Gradual | |  |  |  |
| Mammals | M2 | 192 | 0.86 | 41.86 | Lowland-Montane | BcstDcst | 0.12 | NA | 0 | NA | Gradual | |  |  |  |
| Mammals | M3 | 20 | 0.69 | 22.5 | Lowland-Montane | BTimeVarDTimeVar | 0.01 | 0.36 | 0.03 | 0.33 | Declining | |  |  |  |
| Mammals | M4 | 95 | 0.48 | 21.54 | Lowland-Montane | BcstDcst | 1.07 | NA | 0.97 | NA | Gradual | |  |  |  |
| Mammals | M5 | 43 | 0.42 | 25.49 | Lowland-Montane | BcstDcst | 0.25 | NA | 0.12 | NA | Gradual | |  |  |  |
| Mammals | M6 | 279 | 0.68 | 12.66 | Mixed | BTimeVarDcst | 0.39 | 0.05 | 0 | NA | Saturated | |  |  |  |
| Mammals | M7 | 11 | 0.69 | 10.6 | Lowland-Montane | BcstDcst | 0.17 | NA | 0 | NA | Gradual | |  |  |  |
| Mammals | M8 | 10 | 0.46 | 13.81 | Lowland-Montane | BcstDcst | 0.45 | NA | 0.41 | NA | Gradual | |  |  |  |
| Mammals | M9 | 20 | 0.91 | 34.9 | Lowland-Montane | BTimeVarDcst | 0.02 | 0.06 | 0 | NA | Saturated | |  |  |  |
| Mammals | M10 | 11 | 0.58 | 8.54 | Mixed | BcstDcst | 0.23 | NA | 0 | NA | Gradual | |  |  |  |
| Mammals | M11 | 10 | 1 | 16 | Lowland-Montane | BcstDcst | 0.1 | NA | 0 | NA | Gradual | |  |  |  |
| Mammals | M12 | 199 | 0.82 | 35.28 | Lowland-Montane | BTimeVarDcst | 0.24 | -0.04 | 0 | NA | Exponential | |  |  |  |
| Birds | B1 | 39 | 0.71 | 11.82 | Lowland-Montane | BcstDcst | 0.33 | NA | 0 | NA | Gradual | |  |  |  |
| Birds | B2 | 233 | 0.69 | 26.1 | Mixed | BcstDcst | 0.18 | NA | 0 | NA | Gradual | |  |  |  |
| Birds | B3 | 8 | 0.73 | 24.79 | Lowland-Montane | BcstDcst | 0.3 | NA | 0.36 | NA | Gradual | |  |  |  |
| Birds | B4 | 118 | 0.71 | 28.95 | Mixed | BcstDcst | 0.18 | NA | 0.02 | NA | Gradual | |  |  |  |
| Birds | B5 | 55 | 0.49 | 25.17 | Lowland-Montane | BcstDcst | 0.15 | NA | 0 | NA | Gradual | |  |  |  |
| Birds | B6 | 9 | 0.6 | 23.42 | Lowland-Montane | BcstDcst | 0.09 | NA | 0 | NA | Gradual | |  |  |  |
| Birds | B7 | 7 | 0.64 | 18.55 | Lowland-Montane | BcstDcst | 0.2 | NA | 0.2 | NA | Gradual | |  |  |  |
| Birds | B8 | 165 | 0.71 | 16.82 | Lowland-Montane | BTimeVarDcst | 0.12 | 0.11 | 0 | NA | Saturated | |  |  |  |
| Birds | B9 | 292 | 0.94 | 32.59 | Mixed | BcstDcst | 0.15 | NA | 0 | NA | Gradual | |  |  |  |
| Birds | B10 | 316 | 0.79 | 25.5 | Mixed | BTimeVarDcst | 0.15 | 0.02 | 0 | NA | Saturated | |  |  |  |
| Birds | B11 | 80 | 0.68 | 23.52 | Lowland-Montane | BcstDcst | 0.18 | NA | 0 | NA | Gradual | |  |  |  |
| Birds | B12 | 19 | 0.37 | 20.15 | Lowland-Montane | BcstDcst | 0.2 | NA | 0.04 | NA | Gradual | |  |  |  |
| Birds | B13 | 36 | 0.95 | 17.38 | Lowland-Montane | BTimeVarDcst | 0.1 | 0.06 | 0 | NA | Saturated | |  |  |  |
| Birds | B14 | 39 | 0.85 | 8.2 | Mixed | BTimeVarDcst | 0.06 | 0.37 | 0 | NA | Saturated | |  |  |  |
| Birds | B15 | 17 | 0.94 | 13.11 | Lowland-Montane | BcstDcst | 0.14 | NA | 0 | NA | Gradual | |  |  |  |
| Birds | B16 | 67 | 0.62 | 24.51 | Lowland-Montane | BTimeVarDcst | 0.12 | 0.05 | 0 | NA | Saturated | |  |  |  |
| Birds | B17 | 10 | 0.37 | 13.1 | Lowland-Montane | BcstDcst | 0.19 | NA | 0 | NA | Gradual | |  |  |  |
| Birds | B18 | 89 | 0.99 | 11.21 | Lowland-Montane | BTimeVarDcst | 0.11 | 0.19 | 0 | NA | Saturated | |  |  |  |
| Birds | B19 | 92 | 0.89 | 12.02 | Lowland-Montane | BTimeVarDcst | 0.18 | 0.11 | 0 | NA | Saturated | |  |  |  |
| Birds | B20 | 41 | 0.82 | 18.04 | Lowland-Montane | BTimeVarDcst | 0.05 | 0.13 | 0 | NA | Saturated | |  |  |  |
| Birds | B21 | 309 | 0.77 | 13.57 | Lowland-Montane | BTimeVarDcst | 0.18 | 0.12 | 0.08 | NA | Saturated | |  |  |  |
| Birds | B22 | 20 | 0.87 | 24.67 | Mixed | BcstDcst | 0.1 | NA | 0 | NA | Gradual | |  |  |  |
| Birds | B23 | 25 | 0.76 | 9.37 | Lowland-Montane | BTimeVarDcst | 0.13 | 0.18 | 0 | NA | Saturated | |  |  |  |
| Birds | B24 | 10 | 0.91 | 6.01 | Lowland-Montane | BcstDcst | 0.26 | NA | 0 | NA | Gradual | |  |  |  |
| Birds | B25 | 12 | 0.5 | 10.54 | Lowland-Montane | BcstDcst | 0.21 | NA | 0 | NA | Gradual | |  |  |  |
| Birds | B26 | 12 | 0.86 | 5.38 | Lowland-Montane | BcstDcst | 0.32 | NA | 0 | NA | Gradual | |  |  |  |
| Birds | B27 | 8 | 0.29 | 12.17 | Lowland-Montane | BcstDcst | 0.62 | NA | 0.56 | NA | Gradual | |  |  |  |
| Birds | B28 | 44 | 0.75 | 20.12 | Lowland-Montane | BcstDcst | 0.22 | NA | 0.04 | NA | Gradual | |  |  |  |
| Birds | B29 | 10 | 0.27 | 42.09 | Lowland-Montane | BcstDcst | 0.12 | NA | 0.08 | NA | Gradual | |  |  |  |
| Birds | B30 | 9 | 1 | 9.65 | Lowland-Montane | BcstDcst | 0.15 | NA | 0 | NA | Gradual | |  |  |  |
| Birds | B31 | 12 | 0.71 | 11.06 | Mixed | BcstDcst | 0.2 | NA | 0 | NA | Gradual | |  |  |  |
| Birds | B32 | 13 | 0.76 | 20.68 | Lowland-Montane | BcstDcst | 0.1 | NA | 0 | NA | Gradual | |  |  |  |
| Squamata | S1 | 76 | 0.49 | 34.35 | Lowland-Montane | BTimeVarDcst | 0.06 | 0.04 | 0 | NA | Saturated | |  |  |  |
| Squamata | S2 | 15 | 0.38 | 26.03 | Lowland-Montane | BTimeVarDcst | 0.04 | 0.07 | 0 | NA | Saturated | |  |  |  |
| Squamata | S3 | 19 | 0.24 | 21.78 | Lowland-Montane | BcstDcst | 0.28 | NA | 0.13 | NA | Gradual | |  |  |  |
| Squamata | S4 | 13 | 0.62 | 21.14 | Lowland-Montane | BcstDcst | 0.1 | NA | 0 | NA | Gradual | |  |  |  |
| Squamata | S5 | 43 | 0.61 | 21.41 | Lowland-Montane | BcstDcst | 0.16 | NA | 0 | NA | Gradual | |  |  |  |
| Squamata | S6 | 15 | 0.47 | 44.84 | Lowland-Montane | BcstDcst | 0.06 | NA | 0 | NA | Gradual | |  |  |  |
| Squamata | S7 | 9 | 0.26 | 80.54 | Lowland-Montane | BcstDcst | 0.23 | NA | 0.24 | NA | Gradual | |  |  |  |
| Squamata | S8 | 29 | 0.49 | 43.74 | Lowland-Montane | BTimeVarDTimeVar | 0.58 | -0.06 | 1.65 | -0.29 | Declining | |  |  |  |
| Squamata | S9 | 214 | 0.49 | 83.4 | Lowland-Montane | BTimeVarDcst | 0.06 | 0.01 | 0 | NA | Saturated | |  |  |  |
| Squamata | S10 | 119 | 0.39 | 71.14 | Montane-Highland | BTimeVarDcst | 0.23 | -0.04 | 0 | NA | Exponential | |  |  |  |
| Squamata | S11 | 11 | 0.33 | 42.19 | Lowland-Montane | BcstDcst | 0.06 | NA | 0 | NA | Gradual | |  |  |  |
| Squamata | S12 | 12 | 0.44 | 81.21 | Lowland-Montane | BcstDcst | 0.03 | NA | 0 | NA | Gradual | |  |  |  |
| Squamata | S13 | 44 | 0.43 | 19.79 | Mixed | BTimeVarDcst | 0.12 | 0.05 | 0 | NA | Saturated | |  |  |  |
| Squamata | S14 | 22 | 0.76 | 34.56 | Lowland-Montane | BcstDcst | 0.12 | NA | 0.06 | NA | Gradual | |  |  |  |
| Squamata | S15 | 78 | 0.57 | 88.47 | Lowland-Montane | BcstDcst | 0.05 | NA | 0 | NA | Gradual | |  |  |  |
| Squamata | S16 | 18 | 0.42 | 26.47 | Lowland-Montane | BcstDcst | 0.11 | NA | 0.01 | NA | Gradual | |  |  |  |
| Squamata | S17 | 37 | 0.39 | 48.84 | Lowland-Montane | BTimeVarDcst | 0.02 | 0.04 | 0 | NA | Saturated | |  |  |  |
| Squamata | S18 | 144 | 0.43 | 86.27 | Mixed | BcstDcst | 0.06 | NA | 0 | NA | Gradual | |  |  |  |
| Squamata | S19 | 20 | 0.33 | 25.22 | Lowland-Montane | BcstDcst | 0.12 | NA | 0 | NA | Gradual | |  |  |  |
| Squamata | S20 | 16 | 0.84 | 36.33 | Lowland-Montane | BTimeVarDcst | 0.02 | 0.06 | 0 | NA | Saturated | |  |  |  |
| Squamata | S21 | 20 | 0.31 | 66.91 | Lowland-Montane | BcstDcst | 0.06 | NA | 0 | NA | Gradual | |  |  |  |
| Squamata | S22 | 69 | 0.41 | 70.89 | Lowland-Montane | BTimeVarDTimeVar | 0.1 | 0 | 4.01 | -2.25 | Declining | |  |  |  |
| Squamata | S23 | 60 | 0.49 | 34.42 | Mixed | BcstDcst | 0.11 | NA | 0 | NA | Gradual | |  |  |  |
| Squamata | S24 | 45 | 0.14 | 29.25 | Lowland-Montane | BTimeVarDcst | 0.11 | 0.03 | 0 | NA | Saturated | |  |  |  |
| Amphibia | A1 | 118 | 0.93 | 67.18 | Lowland-Montane | BTimeVarDcst | 0.05 | 0.01 | 0 | NA | Saturated | |  |  |  |
| Amphibia | A2 | 136 | 1 | 67.35 | Mixed | BcstDcst | 0.06 | NA | 0 | NA | Gradual | |  |  |  |
| Amphibia | A3 | 86 | 0.79 | 80.69 | Lowland-Montane | BcstDcst | 0.04 | NA | 0 | NA | Gradual | |  |  |  |
| Amphibia | A4 | 170 | 0.78 | 72.08 | Mixed | BTimeVarDTimeVar | 0.04 | 0.05 | 0.01 | 0.08 | Exponential | |  |  |  |
| Amphibia | A5 | 61 | 0.47 | 69.87 | Lowland-Montane | BTimeVarDcst | 0.04 | 0.01 | 0 | NA | Saturated | |  |  |  |
| Amphibia | A6 | 295 | 0.47 | 68.16 | Mixed | BTimeVarDcst | 0.07 | 0.01 | 0 | NA | Saturated | |  |  |  |
| Amphibia | A7 | 50 | 0.79 | 54.78 | Lowland-Montane | BcstDcst | 0.06 | NA | 0 | NA | Gradual | |  |  |  |
| Amphibia | A8 | 117 | 0.64 | 69.93 | Lowland-Montane | BcstDcst | 0.09 | NA | 0.03 | NA | Gradual | |  |  |  |
| Amphibia | A9 | 147 | 0.58 | 72.8 | Amazonian | Tropical forest | Lowland-Montane | BcstDcst | | 0.07 | NA | 0 | | NA | Gradual |
| Amphibia | A10 | 60 | 0.71 | 43.33 | Amazonian | Tropical forest | Lowland-Montane | BcstDcst | | 0.08 | NA | 0 | | NA | Gradual |
| Amphibia | A11 | 127 | 0.49 | 87.37 | Mixed | Mixed | Lowland-Montane | BTimeVarDTimeVar | | 0.28 | -0.02 | 0.37 | | -0.05 | Declining |
| Amphibia | A12 | 186 | 0.88 | 78.07 | Mixed | Mixed | Lowland-Montane | BcstDcst | | 0.06 | NA | 0 | | NA | Gradual |
| Amphibia | A13 | 128 | 0.81 | 33.4 | Andean | Tropical forest | Lowland-Montane | BTimeVarDcst | | 0.07 | 0.05 | 0 | | NA | Saturated |
| Amphibia | A14 | 54 | 0.45 | 53.95 | Andean | Mixed | Mixed | BcstDcst | | 0.43 | NA | 0.4 | | NA | Gradual |
| Amphibia | A15 | 110 | 0.7 | 33.89 | Andean | Mixed | Mixed | BcstDcst | | 0.16 | NA | 0.06 | | NA | Gradual |
| Amphibia | A16 | 159 | 0.52 | 73.05 | Andean | Tropical forest | Lowland-Montane | BcstDcst | | 0.07 | NA | 0 | | NA | Gradual |

**Supplementary file B.**

Summary results showing the best-fit model for each clade (among constant, time-, temperature- and Andean uplift-dependent models) and the derived species richness pattern (gradual increase [Sc. 1], exponential increase [Sc. 2], saturated increase [Sc. 3] and decline [Sc. 4]). For each model category, we fitted three models in which speciation (Birth “B”) and/or extinction (Death “D”) remain constant (“cst”), or vary (“Var.”) continuously with time, with temperature changes, or with the elevation of the Andes. The value of speciation (lambda) and extinction (mu) rates is provided, as well as the value of the dependence between speciation and extinction rates with the environmental variables (alpha and beta values, respectively). For time-dependent models, *α* and *β > 0* reflect decreasing speciation and extinction towards the present, respectively, while *α* and *β <0* indicate the opposite, increasing speciation and extinction towards the present. For temperature models, *α* and *β > 0* reflect decreasing speciation and extinction with decreasing temperatures, respectively, and conversely. For the uplift models, *α* and *β < 0* reflect increasing speciation and extinction with increasing Andean elevations, respectively, and conversely. Species richness patterns are derived from the diversification trend through time. For each phylogeny, the sampling fraction (*i.e.* number of species sampled from the total described), crown age (in million years ago), and number of species (#spp) is provided, together with the main elevation range (lowland, montane, highland) of the species in the clade. Mixed patterns could be observed in this category. Abbreviations for model type; B = Birth; D = Death; cst = constant; Var. = variable; Temp. = Temperature; Ande = Andean Uplift model.

| Taxa | Clade | # spp. | Sampling frac. | Crown age | Elevation | Model type | Model category | Lambda | Alpha | Mu | Beta | Richness pattern |
| --- | --- | --- | --- | --- | --- | --- | --- | --- | --- | --- | --- | --- |
| Plants | P1 | 13 | 0.12 | 2.88 | Lowland-Montane | BcstDTimeVar | Time | 2.08 | NA | 0.02 | 2.52 | Exponential |
| Plants | P2 | 38 | 0.28 | 0.94 | Lowland-Montane | BcstDTimeVar | Time | 6.43 | NA | 0.15 | 5.64 | Exponential |
| Plants | P3 | 12 | 0.16 | 43.02 | Lowland-Montane | BcstDcst | Constant | 0.15 | NA | 0.09 | NA | Gradual |
| Plants | P4 | 10 | 0.12 | 13.8 | Lowland-Montane | BcstDcst | Constant | 1.02 | NA | 0.89 | NA | Gradual |
| Plants | P5 | 10 | 0.12 | 7.32 | Lowland-Montane | BcstDcst | Constant | 1.35 | NA | 1.04 | NA | Gradual |
| Plants | P6 | 14 | 0.45 | 5.82 | Lowland-Montane | BcstDcst | Constant | 0.47 | NA | 0 | NA | Gradual |
| Plants | P7 | 29 | 0.57 | 12.27 | Lowland-Montane | BcstDcst | Constant | 0.29 | NA | 0 | NA | Gradual |
| Plants | P8 | 15 | 0.14 | 11.88 | Lowland-Montane | BcstDTemp.Var | Temperature | 0.94 | NA | 0.13 | 0.41 | Exponential |
| Plants | P9 | 10 | 0.15 | 19.63 | Lowland-Montane | BcstDcst | Constant | 0.18 | NA | 0 | NA | Gradual |
| Plants | P10 | 24 | 0.41 | 25.52 | Lowland-Montane | BcstDcst | Constant | 0.14 | NA | 0 | NA | Gradual |
| Plants | P11 | 42 | 0.68 | 15.15 | Lowland-Montane | BcstDcst | Constant | 0.43 | NA | 0.24 | NA | Gradual |
| Plants | P12 | 15 | 0.35 | 13.04 | Lowland-Montane | BcstDcst | Constant | 0.3 | NA | 0.08 | NA | Gradual |
| Plants | P13 | 46 | 0.32 | 56.31 | Lowland-Montane | BTemp.VarDcst | Temperature | 0.21 | 0.06 | 0.31 | NA | Declining |
| Plants | P14 | 10 | 0.2 | 26 | Lowland-Montane | BcstDcst | Constant | 0.43 | NA | 0.38 | NA | Gradual |
| Plants | P15 | 19 | 0.63 | 19.88 | Lowland-Montane | BcstDcst | Constant | 0.19 | NA | 0.05 | NA | Gradual |
| Plants | P16 | 19 | 0.21 | 7.08 | Lowland-Montane | BcstDcst | Constant | 1.14 | NA | 0.69 | NA | Gradual |
| Plants | P17 | 18 | 0.18 | 22.42 | Lowland-Montane | BcstDcst | Constant | 0.27 | NA | 0.14 | NA | Gradual |
| Plants | P18 | 7 | 0.88 | 10.8 | Lowland-Montane | BcstDcst | Constant | 0.11 | NA | 0 | NA | Gradual |
| Plants | P19 | 22 | 0.18 | 28.02 | Lowland-Montane | BcstDTimeVar | Time | 0.21 | NA | 0 | 0.39 | Exponential |
| Plants | P20 | 29 | 0.72 | 20.25 | Lowland-Montane | BcstDcst | Constant | 0.14 | NA | 0 | NA | Gradual |
| Plants | P21 | 103 | 0.41 | 20.77 | Lowland-Montane | BTemp.VarDTemp.Var | Temperature | 0.27 | 0.07 | 8 | -1.26 | Declining |
| Plants | P22 | 19 | 0.43 | 17.64 | Montane-Highland | BcstDcst | Constant | 0.17 | NA | 0 | NA | Gradual |
| Plants | P23 | 9 | 0.56 | 25.05 | Lowland-Montane | BcstDcst | Constant | 0.13 | NA | 0.07 | NA | Gradual |
| Plants | P24 | 200 | 0.36 | 17.03 | Mixed | BTimeVarDcst | Time | 1.64 | -0.29 | 0 | NA | Exponential |
| Plants | P25 | 24 | 0.89 | 19.21 | Lowland-Montane | BcstDcst | Constant | 0.14 | NA | 0 | NA | Gradual |
| Plants | P26 | 43 | 0.61 | 14.63 | Lowland-Montane | BcstDcst | Constant | 0.23 | NA | 0 | NA | Gradual |
| Plants | P27 | 19 | 0.54 | 4.67 | Lowland-Montane | BcstDcst | Constant | 0.74 | NA | 0.17 | NA | Gradual |
| Plants | P28 | 37 | 0.28 | 2.75 | Montane-Highland | BcstDTimeVar | Time | 2.18 | NA | 0 | 4.34 | Exponential |
| Plants | P29 | 13 | 0.93 | 15.29 | Lowland-Montane | BTemp.VarDcst | Temperature | 0.02 | 0.32 | 0 | NA | Saturated |
| Plants | P30 | 14 | 0.88 | 37.32 | Lowland-Montane | BcstDcst | Constant | 0.1 | NA | 0.06 | NA | Gradual |
| Plants | P31 | 78 | 0.98 | 30.97 | Lowland-Montane | BcstDcst | Constant | 0.19 | NA | 0.08 | NA | Gradual |
| Plants | P32 | 22 | 0.79 | 20.51 | Lowland-Montane | BcstDcst | Constant | 0.14 | NA | 0 | NA | Gradual |
| Plants | P33 | 16 | 1 | 19.96 | Lowland-Montane | BcstDcst | Constant | 0.09 | NA | 0 | NA | Gradual |
| Plants | P34 | 32 | 1 | 29.68 | Lowland-Montane | BcstDcst | Constant | 0.21 | NA | 0.14 | NA | Gradual |
| Plants | P35 | 114 | 0.99 | 38.07 | Lowland-Montane | BTemp.VarDcst | Temperature | 0.09 | 0.07 | 0 | NA | Saturated |
| Plants | P36 | 154 | 0.99 | 27.09 | Lowland-Montane | BcstDTemp.Var | Temperature | 0.21 | NA | 0 | 1.44 | Exponential |
| Plants | P37 | 178 | 0.99 | 43.62 | Lowland-Montane | BTemp.VarDTemp.Var | Temperature | 0.11 | 0.17 | 0 | 0.65 | Declining |
| Plants | P38 | 102 | 1 | 26.92 | Lowland-Montane | BcstDTimeVar | Time | 0.25 | NA | 0.01 | 0.18 | Exponential |
| Plants | P39 | 22 | 1 | 34.21 | Lowland-Montane | BcstDcst | Constant | 0.26 | NA | 0.26 | NA | Gradual |
| Plants | P40 | 271 | 0.46 | 20.39 | Lowland-Montane | BTemp.VarDcst | Temperature | 0.9 | -0.19 | 0.01 | NA | Exponential |
| Plants | P41 | 670 | 0.13 | 17.87 | Montane-Highland | BcstDcst | Constant | 3.08 | NA | 2.76 | NA | Gradual |
| Plants | P42 | 789 | 0.21 | 31.48 | Mixed | BTemp.VarDcst | Temperature | 0.83 | -0.19 | 0 | NA | Exponential |
| Plants | P43 | 168 | 0.25 | 21.01 | Lowland-Montane | BcstDTimeVar | Time | 0.71 | NA | 0.31 | 0.05 | Exponential |
| Plants | P44 | 126 | 0.33 | 6 | Lowland-Montane | BTimeVarDcst | Time | 3.76 | -0.68 | 0 | NA | Exponential |
| Plants | P45 | 588 | 0.49 | 48.45 | Lowland-Montane | BcstDAnde.Var | Uplift | 0.17 | NA | 0.2 | 0 | Exponential |
| Plants | P46 | 111 | 0.79 | 28.92 | Lowland-Montane | BTemp.VarDcst | Temperature | 0.09 | 0.11 | 0 | NA | Saturated |
| Plants | P47 | 21 | 1 | 28.86 | Lowland-Montane | BcstDcst | Constant | 0.34 | NA | 0.34 | NA | Gradual |
| Plants | P48 | 50 | 0.23 | 32.21 | Lowland-Montane | BcstDcst | Constant | 3.13 | NA | 3.13 | NA | Gradual |
| Plants | P49 | 44 | 0.3 | 30.99 | Montane-Highland | BcstDcst | Constant | 1.18 | NA | 1.14 | NA | Gradual |
| Plants | P50 | 63 | 0.71 | 7.21 | Lowland-Montane | BcstDcst | Constant | 0.59 | NA | 0 | NA | Gradual |
| Plants | P51 | 189 | 0.44 | 21.79 | Lowland-Montane | BTemp.VarDcst | Temperature | 0.61 | -0.17 | 0 | NA | Exponential |
| Plants | P52 | 495 | 0.32 | 20.79 | Mixed | BTimeVarDcst | Time | 0.59 | -0.07 | 0 | NA | Exponential |
| Plants | P53 | 86 | 0.77 | 30.28 | Lowland-Montane | BcstDcst | Constant | 0.15 | NA | 0 | NA | Gradual |
| Plants | P54 | 38 | 0.56 | 45.01 | Lowland-Montane | BcstDTemp.Var | Temperature | 0.14 | NA | 0 | 0.79 | Declining |
| Plants | P55 | 120 | 0.46 | 19.5 | Lowland-Montane | BTemp.VarDcst | Temperature | 1.7 | -0.38 | 0 | NA | Exponential |
| Plants | P56 | 150 | 0.25 | 27.88 | Lowland-Montane | BTemp.VarDTemp.Var | Temperature | 0.09 | 0.47 | 0.1 | 0.46 | Declining |
| Plants | P57 | 161 | 0.25 | 48.28 | Lowland-Montane | BcstDcst | Constant | 0.18 | NA | 0.07 | NA | Gradual |
| Plants | P58 | 307 | 0.24 | 42.38 | Lowland-Montane | BTemp.VarDcst | Temperature | 0.51 | -0.13 | 0.08 | NA | Exponential |
| Plants | P59 | 34 | 0.35 | 24.17 | Lowland-Montane | BcstDTimeVar | Time | 0.43 | NA | 0.08 | 0.15 | Exponential |
| Plants | P60 | 16 | 0.18 | 35.74 | Lowland-Montane | BcstDTimeVar | Time | 0.17 | NA | 0 | 0.25 | Exponential |
| Plants | P61 | 14 | 0.13 | 8.68 | Lowland-Montane | BcstDcst | Constant | 0.51 | NA | 0.07 | NA | Gradual |
| Plants | P62 | 10 | 0.4 | 1.65 | Lowland-Montane | BcstDcst | Constant | 1.7 | NA | 0 | NA | Gradual |
| Plants | P63 | 10 | 0.42 | 0.88 | Lowland-Montane | BcstDcst | Constant | 2.23 | NA | 0 | NA | Gradual |
| Plants | P64 | 10 | 0.59 | 4.57 | Lowland-Montane | BcstDcst | Constant | 0.42 | NA | 0 | NA | Gradual |
| Plants | P65 | 32 | 0.11 | 21.51 | Montane-Highland | BcstDcst | Constant | 0.71 | NA | 0.55 | NA | Gradual |
| Plants | P66 | 48 | 0.28 | 0.5 | Lowland-Montane | BcstDcst | Constant | 8.82 | NA | 0 | NA | Gradual |
| Mammals | M1 | 32 | 1 | 67.96 | Lowland-Montane | BcstDcst | Constant | 0.08 | NA | 0.05 | NA | Gradual |
| Mammals | M2 | 192 | 0.86 | 41.86 | Lowland-Montane | BTemp.VarDTemp.Var | Temperature | 0.06 | 0.3 | 0.06 | 0.3 | Saturated |
| Mammals | M3 | 20 | 0.69 | 22.5 | Lowland-Montane | BTimeVarDTimeVar | Time | 0.01 | 0.36 | 0.03 | 0.33 | Declining |
| Mammals | M4 | 95 | 0.48 | 21.54 | Lowland-Montane | BTemp.VarDcst | Temperature | 1.19 | -0.34 | 0 | NA | Exponential |
| Mammals | M5 | 43 | 0.42 | 25.49 | Lowland-Montane | BcstDcst | Constant | 0.25 | NA | 0.12 | NA | Gradual |
| Mammals | M6 | 279 | 0.68 | 12.66 | Mixed | BTemp.VarDcst | Temperature | 0.33 | 0.1 | 0 | NA | Saturated |
| Mammals | M7 | 11 | 0.69 | 10.6 | Lowland-Montane | BcstDcst | Constant | 0.17 | NA | 0 | NA | Gradual |
| Mammals | M8 | 10 | 0.46 | 13.81 | Lowland-Montane | BcstDcst | Constant | 0.45 | NA | 0.41 | NA | Gradual |
| Mammals | M9 | 20 | 0.91 | 34.9 | Lowland-Montane | BTimeVarDcst | Time | 0.02 | 0.06 | 0 | NA | Saturated |
| Mammals | M10 | 11 | 0.58 | 8.54 | Mixed | BcstDcst | Constant | 0.23 | NA | 0 | NA | Gradual |
| Mammals | M11 | 10 | 1 | 16 | Lowland-Montane | BcstDcst | Constant | 0.1 | NA | 0 | NA | Gradual |
| Mammals | M12 | 199 | 0.82 | 35.28 | Lowland-Montane | BTemp.VarDTemp.Var | Temperature | 0.14 | 0.36 | 0.18 | 0.33 | Declining |
| Birds | B1 | 39 | 0.71 | 11.82 | Lowland-Montane | BcstDcst | Constant | 0.33 | NA | 0 | NA | Gradual |
| Birds | B2 | 233 | 0.69 | 26.1 | Mixed | BTemp.VarDcst | Temperature | 0.14 | 0.07 | 0.01 | NA | Saturated |
| Birds | B3 | 8 | 0.73 | 24.79 | Lowland-Montane | BcstDcst | Constant | 0.3 | NA | 0.36 | NA | Gradual |
| Birds | B4 | 118 | 0.71 | 28.95 | Mixed | BcstDcst | Constant | 0.18 | NA | 0.02 | NA | Gradual |
| Birds | B5 | 55 | 0.49 | 25.17 | Lowland-Montane | BTemp.VarDcst | Temperature | 0.08 | 0.12 | 0 | NA | Saturated |
| Birds | B6 | 9 | 0.6 | 23.42 | Lowland-Montane | BcstDcst | Constant | 0.09 | NA | 0 | NA | Gradual |
| Birds | B7 | 7 | 0.64 | 18.55 | Lowland-Montane | BcstDcst | Constant | 0.2 | NA | 0.2 | NA | Gradual |
| Birds | B8 | 165 | 0.71 | 16.82 | Lowland-Montane | BTemp.VarDcst | Temperature | 0.07 | 0.25 | 0 | NA | Saturated |
| Birds | B9 | 292 | 0.94 | 32.59 | Mixed | BTemp.VarDcst | Temperature | 0.11 | 0.07 | 0 | NA | Saturated |
| Birds | B10 | 316 | 0.79 | 25.5 | Mixed | BTemp.VarDcst | Temperature | 0.13 | 0.06 | 0 | NA | Saturated |
| Birds | B11 | 80 | 0.68 | 23.52 | Lowland-Montane | BcstDcst | Constant | 0.18 | NA | 0 | NA | Gradual |
| Birds | B12 | 19 | 0.37 | 20.15 | Lowland-Montane | BcstDcst | Constant | 0.2 | NA | 0.04 | NA | Gradual |
| Birds | B13 | 36 | 0.95 | 17.38 | Lowland-Montane | BTemp.VarDcst | Temperature | 0.06 | 0.2 | 0 | NA | Saturated |
| Birds | B14 | 39 | 0.85 | 8.2 | Mixed | BTimeVarDcst | Time | 0.06 | 0.37 | 1 | NA | Saturated |
| Birds | B15 | 17 | 0.94 | 13.11 | Lowland-Montane | BcstDcst | Constant | 0.14 | NA | 0 | NA | Gradual |
| Birds | B16 | 67 | 0.62 | 24.51 | Lowland-Montane | BTemp.VarDcst | Temperature | 0.05 | 0.21 | 0 | NA | Saturated |
| Birds | B17 | 10 | 0.37 | 13.1 | Lowland-Montane | BcstDcst | Constant | 0.19 | NA | 0 | NA | Gradual |
| Birds | B18 | 89 | 0.99 | 11.21 | Lowland-Montane | BTemp.VarDcst | Temperature | 0.05 | 0.41 | 0.08 | NA | Declining |
| Birds | B19 | 92 | 0.89 | 12.02 | Lowland-Montane | BTemp.VarDcst | Temperature | 0.1 | 0.27 | 0 | NA | Saturated |
| Birds | B20 | 41 | 0.82 | 18.04 | Lowland-Montane | BTemp.VarDcst | Temperature | 0.02 | 0.34 | 0 | NA | Saturated |
| Birds | B21 | 309 | 0.77 | 13.57 | Lowland-Montane | BTemp.VarDcst | Temperature | 0.15 | 0.3 | 0.37 | NA | Declining |
| Birds | B22 | 20 | 0.87 | 24.67 | Mixed | BcstDcst | Constant | 0.1 | NA | 0 | NA | Gradual |
| Birds | B23 | 25 | 0.76 | 9.37 | Lowland-Montane | BTemp.VarDcst | Temperature | 0.06 | 0.38 | 0 | NA | Saturated |
| Birds | B24 | 10 | 0.91 | 6.01 | Lowland-Montane | BcstDcst | Constant | 0.26 | NA | 0 | NA | Gradual |
| Birds | B25 | 12 | 0.5 | 10.54 | Lowland-Montane | BcstDcst | Constant | 0.21 | NA | 0 | NA | Gradual |
| Birds | B26 | 12 | 0.86 | 5.38 | Lowland-Montane | BcstDcst | Constant | 0.32 | NA | 0 | NA | Gradual |
| Birds | B27 | 8 | 0.29 | 12.17 | Lowland-Montane | BcstDcst | Constant | 0.62 | NA | 0.56 | NA | Gradual |
| Birds | B28 | 44 | 0.75 | 20.12 | Lowland-Montane | BcstDcst | Constant | 0.22 | NA | 0.04 | NA | Gradual |
| Birds | B29 | 10 | 0.27 | 42.09 | Lowland-Montane | BcstDcst | Constant | 0.12 | NA | 0.08 | NA | Gradual |
| Birds | B30 | 9 | 1 | 9.65 | Lowland-Montane | BcstDcst | Constant | 0.15 | NA | 0 | NA | Gradual |
| Birds | B31 | 12 | 0.71 | 11.06 | Mixed | BcstDcst | Constant | 0.2 | NA | 0 | NA | Gradual |
| Birds | B32 | 13 | 0.76 | 20.68 | Lowland-Montane | BcstDcst | Constant | 0.1 | NA | 0 | NA | Gradual |
| Squamata | S1 | 76 | 0.49 | 34.35 | Lowland-Montane | BTemp.VarDcst | Temperature | 0.04 | 0.16 | 0 | NA | Saturated |
| Squamata | S2 | 15 | 0.38 | 26.03 | Lowland-Montane | BTimeVarDcst | Time | 0.04 | 0.07 | 0 | NA | Saturated |
| Squamata | S3 | 19 | 0.24 | 21.78 | Lowland-Montane | BcstDcst | Constant | 0.28 | NA | 0.13 | NA | Gradual |
| Squamata | S4 | 13 | 0.62 | 21.14 | Lowland-Montane | BcstDcst | Constant | 0.1 | NA | 0 | NA | Gradual |
| Squamata | S5 | 43 | 0.61 | 21.41 | Lowland-Montane | BTemp.VarDcst | Temperature | 0.08 | 0.16 | 0 | NA | Saturated |
| Squamata | S6 | 15 | 0.47 | 44.84 | Lowland-Montane | BcstDcst | Constant | 0.06 | NA | 0 | NA | Gradual |
| Squamata | S7 | 9 | 0.26 | 80.54 | Lowland-Montane | BcstDcst | Constant | 0.23 | NA | 0.24 | NA | Gradual |
| Squamata | S8 | 29 | 0.49 | 43.74 | Lowland-Montane | BTimeVarDTimeVar | Time | 0.58 | -0.06 | 1.65 | -0.29 | Declining |
| Squamata | S9 | 214 | 0.49 | 83.4 | Lowland-Montane | BAnde.VarDcst | Uplift | 0.14 | 0 | 0 | NA | Saturated |
| Squamata | S10 | 119 | 0.39 | 71.14 | Montane-Highland | BAnde.VarDAnde.Var | Uplift | 0.1 | 0 | 0.18 | 0 | Exponential |
| Squamata | S11 | 11 | 0.33 | 42.19 | Lowland-Montane | BAnde.VarDcst | Uplift | 0.17 | 0 | 0 | NA | Saturated |
| Squamata | S12 | 12 | 0.44 | 81.21 | Lowland-Montane | BAnde.VarDAnde.Var | Uplift | 0.3 | 0 | 0.66 | 0 | Exponential |
| Squamata | S13 | 44 | 0.43 | 19.79 | Mixed | BTimeVarDcst | Time | 0.12 | 0.05 | 0 | NA | Saturated |
| Squamata | S14 | 22 | 0.76 | 34.56 | Lowland-Montane | BcstDcst | Constant | 0.12 | NA | 0.06 | NA | Gradual |
| Squamata | S15 | 78 | 0.57 | 88.47 | Lowland-Montane | BAnde.VarDAnde.Var | Uplift | 0.09 | 0 | 0.28 | -0.01 | Exponential |
| Squamata | S16 | 18 | 0.42 | 26.47 | Lowland-Montane | BcstDcst | Constant | 0.11 | NA | 0.01 | NA | Gradual |
| Squamata | S17 | 37 | 0.39 | 48.84 | Lowland-Montane | BTemp.VarDcst | Temperature | 0.01 | 0.22 | 0 | NA | Saturated |
| Squamata | S18 | 144 | 0.43 | 86.27 | Mixed | BAnde.VarDAnde.Var | Uplift | 0.2 | 1 | 0.17 | 0 | Exponential |
| Squamata | S19 | 20 | 0.33 | 25.22 | Lowland-Montane | BTemp.VarDcst | Temperature | 0.03 | 0.29 | 0.06 | NA | Declining |
| Squamata | S20 | 16 | 0.84 | 36.33 | Lowland-Montane | BAnde.VarDcst | Uplift | 0.17 | 0 | 0 | NA | Saturated |
| Squamata | S21 | 20 | 0.31 | 66.91 | Lowland-Montane | BcstDcst | Constant | 0.06 | NA | 0 | NA | Gradual |
| Squamata | S22 | 69 | 0.41 | 70.89 | Lowland-Montane | BAnde.VarDcst | Uplift | 0.08 | 0 | 0 | NA | Saturated |
| Squamata | S23 | 60 | 0.49 | 34.42 | Mixed | BTemp.VarDcst | Temperature | 0.05 | 0.12 | 0 | NA | Saturated |
| Squamata | S24 | 45 | 0.14 | 29.25 | Lowland-Montane | BTemp.VarDcst | Temperature | 0.06 | 0.15 | 0 | NA | Saturated |
| Amphibia | A1 | 118 | 0.93 | 67.18 | Lowland-Montane | BAnde.VarDAnde.Var | Uplift | 0.18 | 0 | 0.16 | 0 | Exponential |
| Amphibia | A2 | 136 | 1 | 67.35 | Mixed | BTemp.VarDcst | Temperature | 0.04 | 0.06 | 0 | NA | Saturated |
| Amphibia | A3 | 86 | 0.79 | 80.69 | Lowland-Montane | BcstDcst | Constant | 0.04 | NA | 0 | NA | Gradual |
| Amphibia | A4 | 170 | 0.78 | 72.08 | Mixed | BAnde.VarDAnde.Var | Uplift | 0.76 | 0 | 0.8 | 0 | Exponential |
| Amphibia | A5 | 61 | 0.47 | 69.87 | Lowland-Montane | BAnde.VarDAnde.Var | Uplift | 0.22 | 0 | 0.21 | 0 | Exponential |
| Amphibia | A6 | 295 | 0.47 | 68.16 | Mixed | BAnde.VarDAnde.Var | Uplift | 0.17 | 0 | 0.11 | 0 | Exponential |
| Amphibia | A7 | 50 | 0.79 | 54.78 | Lowland-Montane | BAnde.VarDcst | Uplift | 0.08 | 0 | 0 | NA | Saturated |
| Amphibia | A8 | 117 | 0.64 | 69.93 | Lowland-Montane | BcstDcst | Constant | 0.09 | NA | 0.03 | NA | Gradual |
| Amphibia | A9 | 147 | 0.58 | 72.8 | Lowland-Montane | BcstDcst | Constant | 0.07 | NA | 0 | NA | Gradual |
| Amphibia | A10 | 60 | 0.71 | 43.33 | Lowland-Montane | BTemp.VarDcst | Temperature | 0.04 | 0.16 | 0.06 | NA | Declining |
| Amphibia | A11 | 127 | 0.49 | 87.37 | Lowland-Montane | BcstDAnde.Var | Uplift | 0.25 | NA | 3 | 0 | Declining |
| Amphibia | A12 | 186 | 0.88 | 78.07 | Lowland-Montane | BAnde.VarDAnde.Var | Uplift | 0.1 | 1 | 0.08 | 0 | Exponential |
| Amphibia | A13 | 128 | 0.81 | 33.4 | Lowland-Montane | BTemp.VarDcst | Temperature | 0.03 | 0.2 | 0 | NA | Saturated |
| Amphibia | A14 | 54 | 0.45 | 53.95 | Mixed | BcstDcst | Constant | 0.43 | NA | 0.4 | NA | Gradual |
| Amphibia | A15 | 110 | 0.7 | 33.89 | Mixed | BcstDcst | Constant | 0.16 | NA | 0.06 | NA | Gradual |
| Amphibia | A16 | 159 | 0.52 | 73.05 | Lowland-Montane | BAnde.VarDAnde.Var | Uplift | 0.19 | 0 | 0.17 | 0 | Exponential |

**Supplementary file C**

Summary results showing the best-fit model for each clade based on Pulled diversification rates (PDR) in *castor*. For each model category, we compared a constant model (1-time interval) with a model in which PDR values are allowed to vary independently on a grid of 3-time intervals (PDR values are presented for the successive intervals going back in time.). We selected the model that best explains the LTT of the Neotropical time trees based on AIC values. Rholambda (*ρλo)* value is the product of present-day speciation *(λo)* and sampling fraction *(ρ)*. Knowing *ρ*, *λ_0_* could be derived as follows: *λ_0_* = *λ_0_ρ/ρ.* Pulled extinction rates “PER” (*μ_p_*) for each time interval could be derived as follows: *μ_p_* := *λ_0_* – *r_p_.* If the estimated *μ_p_(0)* is negative, this is evidence that speciation (*lambda*) is currently decreasing over time.

|  |  | Constant model | | | | Gridded model | | | | | | | | | |  |
| --- | --- | --- | --- | --- | --- | --- | --- | --- | --- | --- | --- | --- | --- | --- | --- | --- |
| Taxa | **Clade** | **PDR** | **rholambda0** | **Lambda** | **AIC** | **PDR1.present** | **PDR2** | **PDR3.past** | **rholambda0** | **lambda0** | **PER1.present** | **PER2** | **PER3.past** | **Lambda trend** | **AIC** | **Best model** |
| Plants | P1 | 1.83 | 0.21 | 1.79 | 33.1 | 20.44 | -0.14 | 1.89 | 0 | 0 | -20.44 | 0.14 | -1.89 | lambda descreases | 30.99 | time-variable |
| Plants | P2 | 5.63 | 1.76 | 6.32 | -12.61 | 20.24 | 1.91 | 5.8 | 0.54 | 1.94 | -19.7 | -1.36 | -5.26 | lambda descreases | -12.97 | time-variable |
| Plants | P3 | 0.09 | 0.02 | 0.13 | 93.34 | 0.52 | -0.06 | 0.26 | 0 | 0.02 | -0.52 | 0.07 | -0.25 | lambda descreases | 94.92 | constant |
| Plants | P4 | 0.22 | 0.1 | 0.82 | 56.25 | 1.92 | -0.43 | 0.9 | 0.01 | 0.08 | -1.92 | 0.44 | -0.89 | lambda descreases | 56.68 | constant |
| Plants | P5 | 0.45 | 0.13 | 1.07 | 46.05 | 1.67 | -0.08 | 1.1 | 0.06 | 0.47 | -1.61 | 0.14 | -1.05 | lambda descreases | 49.35 | constant |
| Plants | P6 | 0.67 | 0.14 | 0.32 | 56.55 | 3.92 | -0.45 | 2.08 | 0.02 | 0.04 | -3.9 | 0.47 | -2.06 | lambda descreases | 57.5 | constant |
| Plants | P7 | 0.34 | 0.14 | 0.25 | 141.95 | 0.5 | 0.27 | 0.43 | 0.12 | 0.22 | -0.38 | -0.15 | -0.3 | lambda descreases | 145.86 | constant |
| Plants | P8 | 0.23 | 0.22 | 1.62 | 73.25 | 1.72 | -0.45 | 0.95 | 0.06 | 0.42 | -1.66 | 0.51 | -0.89 | lambda descreases | 73.35 | constant |
| Plants | P9 | 0.23 | 0.02 | 0.12 | 63.98 | 0.56 | 0.09 | 0.44 | 0.01 | 0.06 | -0.55 | -0.08 | -0.42 | lambda descreases | 67.63 | constant |
| Plants | P10 | 0.17 | 0.04 | 0.1 | 157.29 | 0.44 | 0.08 | 0.27 | 0.02 | 0.05 | -0.42 | -0.06 | -0.24 | lambda descreases | 160.61 | constant |
| Plants | P11 | 0.21 | 0.28 | 0.41 | 201.43 | 0.25 | 0.15 | 0.38 | 0.28 | 0.4 | 0.02 | 0.12 | -0.1 | unknown | 205.24 | constant |
| Plants | P12 | 0.27 | 0.09 | 0.26 | 81.99 | 0.82 | 0.02 | 0.61 | 0.05 | 0.14 | -0.77 | 0.03 | -0.56 | lambda descreases | 85.27 | constant |
| Plants | P13 | 0.04 | 0.05 | 0.16 | 361.3 | 0.02 | -0.01 | 0.23 | 0.06 | 0.2 | 0.04 | 0.07 | -0.17 | unknown | 359.39 | time-variable |
| Plants | P14 | 0.1 | 0.07 | 0.34 | 67 | 0.52 | -0.09 | 0.31 | 0.03 | 0.14 | -0.49 | 0.12 | -0.28 | lambda descreases | 69.92 | constant |
| Plants | P15 | 0.17 | 0.1 | 0.17 | 112.7 | 0.55 | 0 | 0.35 | 0.06 | 0.09 | -0.5 | 0.06 | -0.29 | lambda descreases | 115.72 | constant |
| Plants | P16 | 0.53 | 0.21 | 1.01 | 77.97 | 1.08 | 0.39 | 0.51 | 0.14 | 0.7 | -0.94 | -0.24 | -0.37 | lambda descreases | 81.69 | constant |
| Plants | P17 | 0.15 | 0.04 | 0.24 | 119.47 | 0.09 | 0.03 | 0.59 | 0.06 | 0.36 | -0.03 | 0.03 | -0.52 | lambda descreases | 120.69 | constant |
| Plants | P18 | 0.46 | 0.02 | 0.02 | 37.11 | 5.23 | 0.02 | 0.66 | 0 | 0 | -5.23 | -0.02 | -0.66 | lambda descreases | 39.55 | constant |
| Plants | P19 | 0.18 | 0.04 | 0.21 | 144.08 | 0.62 | 0.1 | 0.13 | 0.01 | 0.06 | -0.61 | -0.09 | -0.12 | lambda descreases | 145.99 | constant |
| Plants | P20 | 0.18 | 0.09 | 0.12 | 174.5 | 0.17 | 0.13 | 0.35 | 0.1 | 0.13 | -0.07 | -0.04 | -0.25 | lambda descreases | 178.09 | constant |
| Plants | P21 | 0.38 | 0.02 | 0.04 | 584.39 | -0.19 | 0.52 | 0.19 | 0.05 | 0.12 | 0.24 | -0.47 | -0.14 | unknown | 583.32 | time-variable |
| Plants | P22 | 0.27 | 0.04 | 0.09 | 113.05 | 0.68 | 0.17 | 0.32 | 0.02 | 0.04 | -0.66 | -0.15 | -0.31 | lambda descreases | 116.51 | constant |
| Plants | P23 | 0.11 | 0.06 | 0.1 | 60.65 | 0.24 | 0.07 | 0.12 | 0.04 | 0.07 | -0.2 | -0.03 | -0.08 | lambda descreases | 64.56 | constant |
| Plants | P24 | 0.35 | 1 | 2.76 | 550.77 | 1.18 | 0.03 | -0.01 | 0.68 | 1.87 | -0.5 | 0.65 | 0.69 | lambda descreases | 533.76 | time-variable |
| Plants | P25 | 0.22 | 0.08 | 0.09 | 140.25 | 0.99 | -0.02 | 0.42 | 0.02 | 0.02 | -0.97 | 0.04 | -0.4 | lambda descreases | 140.97 | constant |
| Plants | P26 | 0.3 | 0.09 | 0.15 | 228.57 | 0.63 | 0.12 | 0.7 | 0.07 | 0.11 | -0.56 | -0.06 | -0.63 | lambda descreases | 231.23 | constant |
| Plants | P27 | 0.69 | 0.36 | 0.66 | 64.12 | 0.81 | 0.39 | 1.58 | 0.38 | 0.69 | -0.44 | -0.02 | -1.21 | lambda descreases | 67.7 | constant |
| Plants | P28 | 2.1 | 0.56 | 1.97 | 62.72 | 9.06 | 0.55 | 1.89 | 0.1 | 0.36 | -8.96 | -0.44 | -1.79 | lambda descreases | 59.82 | time-variable |
| Plants | P29 | 0.32 | 0.03 | 0.03 | 71.56 | 3.16 | 0.21 | -0.14 | 0 | 0 | -3.16 | -0.21 | 0.14 | lambda descreases | 70.36 | time-variable |
| Plants | P30 | 0.06 | 0.08 | 0.08 | 99.9 | 0.3 | -0.1 | 0.31 | 0.04 | 0.05 | -0.26 | 0.14 | -0.26 | lambda descreases | 101.97 | constant |
| Plants | P31 | 0.12 | 0.18 | 0.18 | 454.1 | 0.37 | -0.05 | 0.41 | 0.12 | 0.12 | -0.25 | 0.17 | -0.29 | lambda descreases | 453.02 | time-variable |
| Plants | P32 | 0.2 | 0.08 | 0.1 | 131.65 | 0.64 | 0.08 | 0.27 | 0.03 | 0.04 | -0.6 | -0.04 | -0.24 | lambda descreases | 134.44 | constant |
| Plants | P33 | 0.15 | 0.06 | 0.06 | 103.23 | -0.26 | 0.2 | 0.32 | 0.14 | 0.14 | 0.4 | -0.06 | -0.18 | unknown | 104.87 | constant |
| Plants | P34 | 0.09 | 0.19 | 0.19 | 190.01 | -0.1 | 0.18 | -0.01 | 0.25 | 0.25 | 0.35 | 0.07 | 0.26 | unknown | 193.08 | constant |
| Plants | P35 | 0.18 | 0.09 | 0.09 | 685.12 | 0.37 | 0.16 | 0 | 0.06 | 0.06 | -0.31 | -0.1 | 0.06 | lambda descreases | 682.02 | time-variable |
| Plants | P36 | 0.2 | 0.19 | 0.19 | 810.35 | 0.38 | 0.15 | 0.13 | 0.14 | 0.15 | -0.24 | 0 | 0.01 | lambda descreases | 811.29 | constant |
| Plants | P37 | 0.19 | 0.17 | 0.17 | 970.84 | 0.46 | 0.11 | 0 | 0.1 | 0.1 | -0.36 | 0 | 0.1 | lambda descreases | 959.66 | time-variable |
| Plants | P38 | 0.21 | 0.25 | 0.25 | 501.35 | 0.64 | 0.07 | 0.13 | 0.14 | 0.14 | -0.49 | 0.08 | 0.01 | lambda descreases | 496.5 | time-variable |
| Plants | P39 | 0.04 | 0.24 | 0.24 | 129.42 | 0.28 | -0.15 | 0.34 | 0.17 | 0.17 | -0.11 | 0.32 | -0.17 | lambda descreases | 131.19 | constant |
| Plants | P40 | 0.29 | 0.4 | 0.88 | 1112.4 | 0.5 | 0.26 | 0 | 0.34 | 0.74 | -0.16 | 0.08 | 0.34 | lambda descreases | 1110.57 | time-variable |
| Plants | P41 | 0.32 | 0.4 | 3.07 | 2712.49 | 0.28 | 0.32 | 0.35 | 0.42 | 3.16 | 0.13 | 0.09 | 0.06 | unknown | 2716.24 | constant |
| Plants | P42 | 0.24 | 0.18 | 0.87 | 4017.59 | 0.5 | 0.15 | 0.24 | 0.13 | 0.62 | -0.36 | -0.02 | -0.11 | lambda descreases | 4001.58 | time-variable |
| Plants | P43 | 0.29 | 0.2 | 0.83 | 805.84 | 0.48 | 0.25 | 0.16 | 0.16 | 0.66 | -0.32 | -0.09 | 0 | lambda descreases | 807.6 | constant |
| Plants | P44 | 1.25 | 1.58 | 4.77 | 164.19 | 5.17 | -0.48 | 1.14 | 0.69 | 2.08 | -4.49 | 1.17 | -0.46 | lambda descreases | 137.61 | time-variable |
| Plants | P45 | 0.2 | 0.06 | 0.13 | 3643.96 | 0.49 | 0.12 | 0.11 | 0.03 | 0.06 | -0.46 | -0.09 | -0.08 | lambda descreases | 3602.6 | time-variable |
| Plants | P46 | 0.24 | 0.08 | 0.1 | 645.59 | 1.12 | 0.02 | 0.21 | 0.01 | 0.01 | -1.11 | -0.01 | -0.2 | lambda descreases | 617.31 | time-variable |
| Plants | P47 | 0.03 | 0.32 | 0.32 | 117.12 | 0.3 | -0.36 | 0.73 | 0.25 | 0.25 | -0.05 | 0.6 | -0.48 | lambda descreases | 115.93 | time-variable |
| Plants | P48 | 0.02 | 0.69 | 3.05 | 229.64 | -0.19 | 0.06 | 0.19 | 0.83 | 3.67 | 1.02 | 0.78 | 0.64 | unknown | 229.73 | constant |
| Plants | P49 | 0.06 | 0.34 | 1.13 | 232.38 | 0.25 | -0.11 | 0.38 | 0.28 | 0.92 | 0.02 | 0.39 | -0.1 | unknown | 233.53 | constant |
| Plants | P50 | 0.65 | 0.39 | 0.55 | 211.5 | 1.36 | 0.4 | 0.74 | 0.28 | 0.39 | -1.09 | -0.13 | -0.46 | lambda descreases | 214.26 | constant |
| Plants | P51 | 0.21 | 0.26 | 0.58 | 929.61 | 0.6 | 0 | 0.48 | 0.17 | 0.39 | -0.43 | 0.17 | -0.31 | lambda descreases | 923.56 | time-variable |
| Plants | P52 | 0.35 | 0.22 | 0.7 | 2245.43 | 0.74 | 0.22 | 0.29 | 0.15 | 0.47 | -0.59 | -0.07 | -0.14 | lambda descreases | 2233.17 | time-variable |
| Plants | P53 | 0.05 | 0.17 | 0.26 | 765.15 | 0.11 | 0.02 | 0.12 | 0.15 | 0.23 | 0.04 | 0.13 | 0.03 | unknown | 767.83 | constant |
| Plants | P54 | 0.07 | 0.1 | 0.18 | 259.13 | 0.27 | -0.04 | 0.21 | 0.06 | 0.11 | -0.21 | 0.1 | -0.15 | lambda descreases | 260.25 | constant |
| Plants | P55 | 0.18 | 0.72 | 1.58 | 437.08 | 0.71 | -0.15 | 0.52 | 0.53 | 1.15 | -0.18 | 0.67 | 0 | lambda descreases | 433.25 | time-variable |
| Plants | P56 | 0.2 | 0.06 | 0.25 | 923.14 | 0.8 | -0.01 | 0.5 | 0.02 | 0.07 | -0.78 | 0.02 | -0.49 | lambda descreases | 906.15 | time-variable |
| Plants | P57 | 0.12 | 0.04 | 0.18 | 1135 | 0.27 | 0.06 | 0.23 | 0.03 | 0.12 | -0.24 | -0.03 | -0.2 | lambda descreases | 1134.35 | time-variable |
| Plants | P58 | 0.12 | 0.13 | 0.52 | 1887.12 | 0.33 | 0.02 | 0.33 | 0.08 | 0.35 | -0.25 | 0.07 | -0.25 | lambda descreases | 1877.14 | time-variable |
| Plants | P59 | 0.17 | 0.2 | 0.57 | 181.13 | 0.93 | -0.14 | 0.3 | 0.07 | 0.2 | -0.86 | 0.21 | -0.23 | lambda descreases | 177.28 | time-variable |
| Plants | P60 | 0.1 | 0.05 | 0.27 | 113.26 | 0.18 | 0.1 | 0.04 | 0.04 | 0.2 | -0.14 | -0.06 | -0.01 | lambda descreases | 116.97 | constant |
| Plants | P61 | 0.5 | 0.06 | 0.44 | 68.04 | 3.38 | -0.38 | 1.55 | 0 | 0.02 | -3.38 | 0.38 | -1.54 | lambda descreases | 68.39 | constant |
| Plants | P62 | 2.45 | 0.49 | 1.22 | 18.1 | 27.41 | -2.59 | 5.8 | 0 | 0.01 | -27.41 | 2.6 | -5.8 | lambda descreases | 17.43 | time-variable |
| Plants | P63 | 4.25 | 0.34 | 0.82 | 10.73 | 48.83 | -20.92 | 42.72 | 0 | 0.01 | -48.82 | 20.93 | -42.72 | lambda descreases | -0.26 | time-variable |
| Plants | P64 | 0.67 | 0.16 | 0.27 | 39.85 | 5.53 | -2.37 | 5.17 | 0.02 | 0.03 | -5.51 | 2.39 | -5.15 | lambda descreases | 37.54 | time-variable |
| Plants | P65 | 0.18 | 0.07 | 0.66 | 198.46 | 0.12 | 0.14 | 0.34 | 0.08 | 0.79 | -0.04 | -0.06 | -0.26 | lambda descreases | 201.77 | constant |
| Plants | P66 | 9.67 | 2.13 | 7.71 | -60.39 | 21.97 | 4.33 | 18.19 | 1.3 | 4.72 | -20.67 | -3.02 | -16.89 | lambda descreases | -57.8 | constant |
| Mammals | M1 | 0.04 | 0.07 | 0.07 | 247.54 | 0.03 | 0.02 | 0.09 | 0.08 | 0.08 | 0.05 | 0.06 | -0.01 | unknown | 251.07 | constant |
| Mammals | M2 | 0.11 | 0.11 | 0.11 | 1245.12 | 0.3 | 0.04 | 0.17 | 0.07 | 0.07 | -0.23 | 0.03 | -0.1 | lambda descreases | 1240.03 | time-variable |
| Mammals | M3 | 0.19 | 0.02 | 0.03 | 133.22 | 0.99 | -0.12 | 0.57 | 0 | 0 | -0.99 | 0.12 | -0.56 | lambda descreases | 133.08 | time-variable |
| Mammals | M4 | 0.11 | 0.5 | 1.05 | 422.38 | 0.5 | -0.23 | 0.77 | 0.38 | 0.8 | -0.12 | 0.61 | -0.39 | lambda descreases | 416.13 | time-variable |
| Mammals | M5 | 0.14 | 0.1 | 0.24 | 265.58 | 0.46 | -0.04 | 0.45 | 0.06 | 0.14 | -0.4 | 0.1 | -0.39 | lambda descreases | 266.33 | constant |
| Mammals | M6 | 0.59 | 0.2 | 0.3 | 1103.75 | 1.06 | 0.47 | 0.48 | 0.14 | 0.21 | -0.92 | -0.33 | -0.34 | lambda descreases | 1102.91 | time-variable |
| Mammals | M7 | 0.4 | 0.04 | 0.06 | 57.98 | 3.73 | -0.42 | 1.3 | 0 | 0 | -3.73 | 0.42 | -1.3 | lambda descreases | 58.11 | constant |
| Mammals | M8 | 0.12 | 0.17 | 0.37 | 56.46 | 0.42 | -0.36 | 1.18 | 0.16 | 0.34 | -0.26 | 0.52 | -1.02 | lambda descreases | 57.66 | constant |
| Mammals | M9 | 0.14 | 0.01 | 0.01 | 146.61 | 1.79 | -0.14 | 0.43 | 0 | 0 | -1.79 | 0.14 | -0.43 | lambda descreases | 141.37 | time-variable |
| Mammals | M10 | 0.52 | 0.04 | 0.08 | 53.91 | -0.69 | 0.78 | 0.57 | 0.15 | 0.26 | 0.84 | -0.62 | -0.42 | unknown | 56.87 | constant |
| Mammals | M11 | 0.14 | 0.09 | 0.09 | 56.08 | 1.69 | -0.25 | 0.27 | 0 | 0 | -1.68 | 0.26 | -0.27 | lambda descreases | 56.15 | constant |
| Mammals | M12 | 0.14 | 0.21 | 0.26 | 1091.66 | 0.32 | 0.05 | 0.21 | 0.16 | 0.2 | -0.16 | 0.12 | -0.05 | lambda descreases | 1090.5 | time-variable |
| Birds | B1 | 0.4 | 0.2 | 0.29 | 174.19 | 2.11 | -0.17 | 0.68 | 0.04 | 0.06 | -2.07 | 0.21 | -0.64 | lambda descreases | 168.55 | time-variable |
| Birds | B2 | 0.22 | 0.1 | 0.14 | 1337.43 | 0.36 | 0.15 | 0.32 | 0.08 | 0.12 | -0.28 | -0.07 | -0.24 | lambda descreases | 1339.49 | constant |
| Birds | B3 | 0.03 | 0.16 | 0.22 | 50.77 | 0.17 | -0.24 | 0.57 | 0.15 | 0.21 | -0.01 | 0.39 | -0.41 | lambda descreases | 53.32 | constant |
| Birds | B4 | 0.17 | 0.13 | 0.18 | 681.82 | 0.45 | 0.06 | 0.26 | 0.08 | 0.12 | -0.37 | 0.02 | -0.18 | lambda descreases | 681.19 | time-variable |
| Birds | B5 | 0.24 | 0.04 | 0.08 | 340.15 | 0.58 | 0.17 | 0.23 | 0.02 | 0.03 | -0.56 | -0.15 | -0.22 | lambda descreases | 342.05 | constant |
| Birds | B6 | 0.15 | 0.04 | 0.06 | 59.97 | 1.32 | -0.15 | 0.4 | 0 | 0 | -1.32 | 0.16 | -0.4 | lambda descreases | 61.16 | constant |
| Birds | B7 | 0.1 | 0.09 | 0.14 | 44.06 | 0.36 | -0.13 | 0.47 | 0.07 | 0.11 | -0.29 | 0.2 | -0.4 | lambda descreases | 47.6 | constant |
| Birds | B8 | 0.39 | 0.06 | 0.08 | 860.41 | 0.71 | 0.26 | 0.74 | 0.04 | 0.06 | -0.67 | -0.22 | -0.7 | lambda descreases | 861.51 | constant |
| Birds | B9 | 0.19 | 0.11 | 0.12 | 1701.47 | 0.48 | 0.07 | 0.34 | 0.07 | 0.07 | -0.41 | 0 | -0.27 | lambda descreases | 1692.72 | time-variable |
| Birds | B10 | 0.22 | 0.11 | 0.14 | 1790.59 | 0.35 | 0.16 | 0.31 | 0.09 | 0.11 | -0.27 | -0.07 | -0.22 | lambda descreases | 1792.32 | constant |
| Birds | B11 | 0.2 | 0.11 | 0.16 | 455.91 | 0.23 | 0.19 | 0.14 | 0.1 | 0.15 | -0.13 | -0.09 | -0.04 | lambda descreases | 459.78 | constant |
| Birds | B12 | 0.18 | 0.07 | 0.18 | 117.74 | 0.66 | -0.05 | 0.52 | 0.03 | 0.08 | -0.63 | 0.08 | -0.49 | lambda descreases | 119.92 | constant |
| Birds | B13 | 0.29 | 0.06 | 0.07 | 202.86 | 1.35 | -0.02 | 0.56 | 0.01 | 0.01 | -1.34 | 0.03 | -0.55 | lambda descreases | 200.96 | time-variable |
| Birds | B14 | 0.7 | 0.03 | 0.03 | 173.07 | 1.33 | 0.3 | 1.56 | 0.02 | 0.02 | -1.31 | -0.27 | -1.54 | lambda descreases | 174.58 | constant |
| Birds | B15 | 0.32 | 0.05 | 0.05 | 94.43 | 0.5 | 0.21 | 0.52 | 0.04 | 0.04 | -0.46 | -0.16 | -0.48 | lambda descreases | 98.23 | constant |
| Birds | B16 | 0.3 | 0.04 | 0.06 | 391.7 | 1.26 | 0.12 | 0.32 | 0 | 0.01 | -1.26 | -0.11 | -0.31 | lambda descreases | 384.19 | time-variable |
| Birds | B17 | 0.35 | 0.03 | 0.07 | 56.36 | 2.5 | -0.14 | 0.88 | 0 | 0 | -2.5 | 0.14 | -0.88 | lambda descreases | 58.25 | constant |
| Birds | B18 | 0.51 | 0.07 | 0.07 | 421.37 | 0.63 | 0.41 | 0.81 | 0.06 | 0.06 | -0.57 | -0.34 | -0.74 | lambda descreases | 424.64 | constant |
| Birds | B19 | 0.53 | 0.1 | 0.11 | 418.1 | 1.03 | 0.43 | 0.39 | 0.06 | 0.06 | -0.98 | -0.38 | -0.33 | lambda descreases | 419.78 | constant |
| Birds | B20 | 0.33 | 0.02 | 0.03 | 235.64 | 1.42 | 0.16 | 0.38 | 0 | 0 | -1.42 | -0.16 | -0.38 | lambda descreases | 235.29 | time-variable |
| Birds | B21 | 0.38 | 0.11 | 0.14 | 1520.64 | 0.18 | 0.34 | 0.76 | 0.14 | 0.19 | -0.04 | -0.2 | -0.62 | lambda descreases | 1516.5 | time-variable |
| Birds | B22 | 0.15 | 0.06 | 0.06 | 129.98 | -0.09 | 0.24 | 0.07 | 0.09 | 0.11 | 0.18 | -0.15 | 0.02 | unknown | 133.38 | constant |
| Birds | B23 | 0.56 | 0.05 | 0.07 | 117.59 | 1.03 | 0.45 | 0.63 | 0.03 | 0.04 | -1 | -0.42 | -0.6 | lambda descreases | 121.36 | constant |
| Birds | B24 | 0.63 | 0.09 | 0.1 | 40.84 | 2.44 | 0.98 | -1.52 | 0.01 | 0.01 | -2.43 | -0.97 | 1.53 | lambda descreases | 41.97 | constant |
| Birds | B25 | 0.53 | 0.02 | 0.04 | 60.54 | 3.11 | 0.18 | 0.75 | 0 | 0 | -3.11 | -0.18 | -0.75 | lambda descreases | 63.14 | constant |
| Birds | B26 | 0.85 | 0.09 | 0.1 | 47.34 | 0.58 | 0.93 | 0.76 | 0.1 | 0.12 | -0.48 | -0.83 | -0.66 | lambda descreases | 51.32 | constant |
| Birds | B27 | 0.17 | 0.13 | 0.46 | 44.53 | 0.64 | -0.28 | 0.96 | 0.1 | 0.34 | -0.54 | 0.37 | -0.86 | lambda descreases | 47.4 | constant |
| Birds | B28 | 0.19 | 0.16 | 0.21 | 240.72 | 0.86 | -0.11 | 0.54 | 0.06 | 0.09 | -0.8 | 0.18 | -0.47 | lambda descreases | 238.59 | time-variable |
| Birds | B29 | 0.07 | 0.03 | 0.1 | 77.49 | 0.36 | -0.06 | 0.25 | 0.01 | 0.03 | -0.35 | 0.07 | -0.24 | lambda descreases | 80.15 | constant |
| Birds | B30 | 0.39 | 0.07 | 0.07 | 46.45 | -0.49 | 0.75 | 0.02 | 0.14 | 0.14 | 0.64 | -0.61 | 0.12 | unknown | 49.94 | constant |
| Birds | B31 | 0.28 | 0.11 | 0.16 | 63.29 | 1.14 | -0.17 | 0.97 | 0.05 | 0.07 | -1.09 | 0.22 | -0.92 | lambda descreases | 65.87 | constant |
| Birds | B32 | 0.16 | 0.05 | 0.06 | 84.52 | 0.25 | 0.02 | 0.48 | 0.05 | 0.06 | -0.2 | 0.02 | -0.43 | lambda descreases | 87.63 | constant |
| Squamata | S1 | 0.15 | 0.02 | 0.04 | 532.96 | 0.73 | -0.02 | 0.37 | 0 | 0.01 | -0.73 | 0.02 | -0.37 | lambda descreases | 524.33 | time-variable |
| Squamata | S2 | 0.18 | 0.01 | 0.03 | 99.13 | 0.58 | 0.08 | 0.28 | 0 | 0.01 | -0.58 | -0.08 | -0.28 | lambda descreases | 102.57 | constant |
| Squamata | S3 | 0.17 | 0.06 | 0.25 | 120.49 | 0.48 | 0.02 | 0.4 | 0.03 | 0.14 | -0.45 | 0.02 | -0.37 | lambda descreases | 123.57 | constant |
| Squamata | S4 | 0.19 | 0.03 | 0.04 | 84.97 | 1.21 | -0.18 | 0.68 | 0 | 0 | -1.21 | 0.18 | -0.68 | lambda descreases | 85.49 | constant |
| Squamata | S5 | 0.27 | 0.05 | 0.08 | 252.87 | 0.56 | 0.2 | 0.27 | 0.03 | 0.04 | -0.53 | -0.17 | -0.24 | lambda descreases | 255.92 | constant |
| Squamata | S6 | 0.12 | 0.01 | 0.02 | 115.61 | 0.06 | 0.15 | 0.06 | 0.01 | 0.02 | -0.05 | -0.14 | -0.05 | lambda descreases | 119.41 | constant |
| Squamata | S7 | 0.02 | 0.05 | 0.18 | 75.41 | 0.15 | -0.12 | 0.22 | 0.03 | 0.1 | -0.12 | 0.14 | -0.19 | lambda descreases | 77.12 | constant |
| Squamata | S8 | 0.07 | 0.05 | 0.1 | 216.36 | -0.24 | 0.19 | 0 | 0.13 | 0.26 | 0.36 | -0.06 | 0.13 | unknown | 215 | time-variable |
| Squamata | S9 | 0.12 | 0.02 | 0.04 | 1624.88 | 0.17 | 0.12 | 0.02 | 0.01 | 0.03 | -0.15 | -0.11 | -0.01 | lambda descreases | 1625.27 | constant |
| Squamata | S10 | 0.1 | 0.1 | 0.26 | 775.73 | 0.31 | 0.04 | 0.02 | 0.05 | 0.14 | -0.26 | 0.02 | 0.04 | lambda descreases | 764.42 | time-variable |
| Squamata | S11 | 0.17 | 0 | 0 | 79.97 | 1.88 | 0.12 | 0.17 | 0 | 0 | -1.88 | -0.12 | -0.17 | lambda descreases | 82.47 | constant |
| Squamata | S12 | 0.08 | 0 | 0.01 | 103.1 | 0.38 | 0.1 | -0.03 | 0 | 0 | -0.38 | -0.1 | 0.03 | lambda descreases | 103.28 | constant |
| Squamata | S13 | 0.25 | 0.04 | 0.08 | 268.07 | 0.5 | 0.12 | 0.49 | 0.02 | 0.06 | -0.48 | -0.09 | -0.46 | lambda descreases | 270.86 | constant |
| Squamata | S14 | 0.08 | 0.08 | 0.11 | 151.94 | 0.07 | 0.04 | 0.21 | 0.09 | 0.12 | 0.02 | 0.05 | -0.12 | unknown | 155.36 | constant |
| Squamata | S15 | 0.08 | 0.02 | 0.03 | 637.92 | 0.2 | 0.06 | 0.04 | 0.01 | 0.01 | -0.19 | -0.05 | -0.03 | lambda descreases | 636.97 | time-variable |
| Squamata | S16 | 0.11 | 0.04 | 0.1 | 126.84 | 0.13 | -0.06 | 0.58 | 0.06 | 0.14 | -0.07 | 0.12 | -0.53 | lambda descreases | 125.94 | time-variable |
| Squamata | S17 | 0.12 | 0 | 0.01 | 293.41 | 0.35 | 0.01 | 0.3 | 0 | 0 | -0.35 | -0.01 | -0.3 | lambda descreases | 293.58 | constant |
| Squamata | S18 | 0.07 | 0.02 | 0.04 | 1192.6 | 0.17 | 0.05 | 0.07 | 0.01 | 0.02 | -0.16 | -0.04 | -0.06 | lambda descreases | 1190.78 | time-variable |
| Squamata | S19 | 0.23 | 0.01 | 0.03 | 131.77 | 0.05 | 0.25 | 0.26 | 0.02 | 0.06 | -0.03 | -0.23 | -0.24 | lambda descreases | 135.48 | constant |
| Squamata | S20 | 0.15 | 0.01 | 0.01 | 117.44 | 0.45 | 0.07 | 0.23 | 0 | 0 | -0.44 | -0.07 | -0.23 | lambda descreases | 120.77 | constant |
| Squamata | S21 | 0.07 | 0.02 | 0.05 | 167.06 | 0.2 | 0.05 | 0.05 | 0 | 0.02 | -0.2 | -0.04 | -0.05 | lambda descreases | 169.92 | constant |
| Squamata | S22 | 0.09 | 0.01 | 0.02 | 577.74 | 0.07 | 0.09 | 0.07 | 0.01 | 0.02 | -0.06 | -0.08 | -0.06 | lambda descreases | 581.62 | constant |
| Squamata | S23 | 0.16 | 0.03 | 0.06 | 413.22 | 0.23 | 0.14 | 0.16 | 0.02 | 0.05 | -0.21 | -0.12 | -0.14 | lambda descreases | 416.97 | constant |
| Squamata | S24 | 0.22 | 0.01 | 0.06 | 304.42 | 0.18 | 0.22 | 0.21 | 0.01 | 0.07 | -0.17 | -0.21 | -0.2 | lambda descreases | 308.4 | constant |
| Amphibia | A1 | 0.08 | 0.04 | 0.04 | 908.19 | 0.1 | 0.09 | 0.05 | 0.03 | 0.03 | -0.07 | -0.05 | -0.02 | lambda descreases | 911.75 | constant |
| Amphibia | A2 | 0.09 | 0.05 | 0.05 | 1012.7 | 0.25 | 0.03 | 0.12 | 0.02 | 0.02 | -0.22 | -0.01 | -0.1 | lambda descreases | 1008.1 | time-variable |
| Amphibia | A3 | 0.06 | 0.03 | 0.03 | 716.31 | 0.14 | 0.03 | 0.09 | 0.02 | 0.02 | -0.12 | -0.02 | -0.07 | lambda descreases | 718.18 | constant |
| Amphibia | A4 | 0.12 | 0.02 | 0.03 | 1264.58 | 0.36 | 0.08 | 0.05 | 0.01 | 0.01 | -0.35 | -0.07 | -0.05 | lambda descreases | 1249.24 | time-variable |
| Amphibia | A5 | 0.09 | 0.01 | 0.03 | 498.94 | 0.23 | 0.06 | 0.08 | 0 | 0.01 | -0.23 | -0.06 | -0.07 | lambda descreases | 499.95 | constant |
| Amphibia | A6 | 0.11 | 0.02 | 0.05 | 2243.42 | 0.16 | 0.1 | 0.07 | 0.02 | 0.04 | -0.14 | -0.09 | -0.05 | lambda descreases | 2244.92 | constant |
| Amphibia | A7 | 0.09 | 0.03 | 0.03 | 390.07 | 0.08 | 0.09 | 0.09 | 0.03 | 0.04 | -0.05 | -0.06 | -0.06 | lambda descreases | 394.05 | constant |
| Amphibia | A8 | 0.06 | 0.06 | 0.09 | 897.33 | 0.12 | 0.03 | 0.14 | 0.04 | 0.07 | -0.07 | 0.02 | -0.09 | lambda descreases | 899.1 | constant |
| Amphibia | A9 | 0.08 | 0.03 | 0.06 | 1142.79 | 0.14 | 0.07 | 0.06 | 0.02 | 0.04 | -0.11 | -0.05 | -0.03 | lambda descreases | 1144.8 | constant |
| Amphibia | A10 | 0.11 | 0.04 | 0.05 | 438.56 | 0.21 | 0.07 | 0.14 | 0.03 | 0.04 | -0.18 | -0.04 | -0.11 | lambda descreases | 441.79 | constant |
| Amphibia | A11 | 0.03 | 0.08 | 0.17 | 1009.67 | -0.07 | 0.05 | 0.08 | 0.13 | 0.26 | 0.2 | 0.08 | 0.05 | unknown | 998.45 | time-variable |
| Amphibia | A12 | 0.08 | 0.04 | 0.04 | 1443.98 | 0.16 | 0.06 | 0.06 | 0.02 | 0.03 | -0.14 | -0.03 | -0.03 | lambda descreases | 1441.95 | time-variable |
| Amphibia | A13 | 0.16 | 0.04 | 0.06 | 863.03 | 0.21 | 0.2 | -0.39 | 0.03 | 0.04 | -0.18 | -0.17 | 0.43 | lambda descreases | 848.46 | time-variable |
| Amphibia | A14 | 0.04 | 0.19 | 0.42 | 344.76 | 0.21 | -0.12 | 0.28 | 0.14 | 0.3 | -0.07 | 0.25 | -0.15 | lambda descreases | 342.17 | time-variable |
| Amphibia | A15 | 0.11 | 0.11 | 0.16 | 704.37 | 0.21 | 0.01 | 0.35 | 0.1 | 0.14 | -0.12 | 0.09 | -0.26 | lambda descreases | 704.01 | time-variable |
| Amphibia | A16 | 0.09 | 0.03 | 0.05 | 1235.92 | 0.27 | 0.04 | 0.09 | 0.01 | 0.02 | -0.26 | -0.04 | -0.08 | lambda descreases | 1226.49 | time-variable |
