# Supplementary material for: Diversification dynamics in the Neotropics through time, clades, and biogeographic regions
Source: eLife. 2022 Oct 27;11:e74503. doi: 10.7554/eLife.74503 (PMC9668338; doi:10.7554/eLife.74503)
Supplement: Supplementary file 2. [file elife-74503-supp2.docx]

**Supplementary file 2**

This file contains all the information on the phylogenetic reconstruction and dating of Caviomorpha.

*Molecular dataset and sequencing.* We obtained a time-calibrated species-level phylogeny for Caviomorpha following the Woods and Kilpatrick (9) taxonomic list, and including recent taxonomic updates for newly described genera and species (10). Five molecular markers proven valuable to resolve caviomorph phylogenies were considered in this study (11–13): two mitochondrial genes (cytochrome *b* apoenzyme: *cyt b* and 12S ribosomal unit: *12S rRNA*) and three nuclear genes (growth hormone receptor exon 10: *GHR*; interphotoreceptor retinoid binding protein exon 1: *IRPB,* and Recombinating Activating protein 1: *RAG1*). DNA cytb and *12S rRNA* sequences were newly generated for the following species: *Proechimys brevicauda, Proechimys cuvieri, Proechimys echinothrix, Proechimys gardneri, Proechimys goeldii, Proechimys gorgonae, Proechimys gularis, Proechimys guyannensis, Proechimys hoplomyoides, Proechimys kulinae, Proechimys longicaudatus, Proechimys mincae, Proechimys pattoni, Proechimys poliopus, Proechimys quadruplicatus, Proechimys roberti, Proechimys semispinosus, Proechimys simonsi, Proechimys steerei, Proechimys trinitatus, Proechimys warreni,*, *Diplomys caniceps* and *Olallamys edax*. Ctenohystrica sequences from previous studies were downloaded from public databanks (Table S2). The final dataset includes 199 caviomorpha species representing 82% of the species and all the genera described (9). We included 37 representatives of Diatomyidae, Ctenodactylidae, Hystricidae, Heterocephalidae, Bathyergidae, Petromuridae and Thryonomyidae, Pedetidae, Dipodidae and Muridae as outgroups (14–16).

Fresh tissues were extracted using the QIAGEN extraction kit protocol. Four museum skin samples of rare unsequenced species (*Proechimys gorgonae, Proechimys trinitatus, Diplomys caniceps* and *Olallamys edax*) were stored in Eppendorf tubes. They were processed in the "Degraded DNA Facility" in Montpellier, France (dedicated to processing low quality/quantity DNA tissue samples). These samples were extracted using the QUIAGEN extraction kit protocol, in small batches (of 3 samples maximum), and a negative control was included in each batch to monitor possible contamination. For all these samples, libraries were prepared following Tilak et al. (17) protocol, in order to obtain the complete mitochondrial genome. These seven libraries were pooled and sequenced without enrichment as single end reads on Illumina HiSeq 2000 lanes at the GATC-Biotech company (Konstanz, Germany). Raw 101-nt reads were imported in Geneious R6 (18) and adaptor fragments were removed by the “trim ends” utility. The mapping of the reads on the phylogenetically closest available mitochondrial genome was performed for each species. The following mapping parameters were used in Geneious read mapper: a minimum of 24 consecutive nucleotides (nt) perfectly matching the reference, a maximum 5% of single nt mismatch over the read length, a minimum of 95%-nt similarity in overlap region, and a maximum of 3% of gaps with a maximum gap size of 3-nt. Iterative mapping cycles were performed in order to elongate the sequence when the complete mitogenome was not recovered after the initial mapping round. A high-quality consensus was generated and the circularity of the mitogenome was verified by the exact superimposition of the 100-nt at the assembly extremities. We extracted the cytb and *12S rRNA* from these mitogenomes in order to perform our phylogenetic analyses We used SEAVIEW (19) and MACSE v2 (20) to align the sequences. They were translated into peptide sequences to exclude putative NUMt copies and to ensure sequence orthology. From these individual alignments, we built four gene matrices; *cyt b* (224 taxa and 1140 sites), *12S* rRNA (152 taxa and 775 sites), *GHR* (95 taxa and 927 sites), *RAG1* (63 taxa and 1064 sites), vWF (83 taxa and 1269 sites) and a nuclear + mitochondrial supermatrix (232 taxa and 5174 sites).

*Phylogenetic analyses.* Phylogenetic trees were reconstructed using a maximum likelihood (ML) method. ML analyses were first carried out on each marker independently and on the supermatrix using RAxML 8.0 (21, 22). Each gene considered separately does not result in a robust phylogeny for Caviomorpha: mitochondrial marker helps to resolve terminal nodes, while nuclear genes lend support to deepest ones. Since the 5 different markers yielded consistent, compatible topologies, sequences were concatenated and phylogenetic analyses were then carried out using the combined dataset. We used PartitionFinder v2.1.1 (23) to find the best partition schemes and nucleotide substitution models. Robustness of each gene tree was assessed using the rapid bootstrap (Bp) procedure (option –f a) with 1,000 replications (option -# numberOfRuns) (21, 22). Divergence times were subsequently estimated from the mitochondrial + nuclear nucleotide supermatrices to provide a temporal framework of the caviomorpha radiation. A Bayesian relaxed molecular clock method was used to estimate divergence dates whilst accounting for changes in evolutionary rates through time and allowing for independent models of sequence evolution for each gene partition. The best fitting substitution models for each partition were selected according to PartitionFinder results (23). We used Beast v1.10.2 (24) for phylogenetic analyses, assuming the Birth Death model of speciation and an uncorrelated log-normal distribution molecular clock as tree priors. Clock models were unlinked across 5 genes and codon partitions of the exons in order to account for missing data (25). We ran MCMC chains for 250 million generations, with trees sampled every 10,000 generations. We performed the analyses 4 times to check for convergence of model parameter estimates, and Tracer (26) was used to assess algorithm convergence. We removed the first 25% of trees as burn in. Trees from each of the 4 independent runs were combined into a maximum clade credibility tree with mean node heights calculated using TreeCombiner and TreeAnnotator. All these analyses were computed on the CIPRES science gateway. To calibrate the phylogeny we selected 17 fossil constraints as described from previous studies (11). In order to take into account uncertainties in the phylogenetic position of these fossils, all constraints were set using hard minimum bounds and soft upper bounds under a lognormal prior, as suggested by recent paleontological studies (27, 28).

**Table S2.** Taxon sampling and GenBank accession numbers for caviomorph rodents.

| Species |  | Genes |  |  |  |  |
| --- | --- | --- | --- | --- | --- | --- |
| MOUSE-RELATED CLADE |  | **cyt-b** | **12SrRNA** | **GHR** | **vWF** | **RAG1** |
| Pedetidae | *Pedetes spce* | AJ389527 | AY012113 | AF332025 | AJ238389 | AY011882 |
| CTENOHYSTRICA |  |  |  |  |  |  |
| CTENODACTYLOMORPHI |  |  |  |  |  |  |
| Diatomyidae | *Laonastes aenigmamus* | AM407933 | DQ139934 | AM407901 | AM407897 |  |
| Ctenodactylidae | *Ctenodactylus vali* | AJ389532 | AJ389543 | AF332042 | JN415077 | JN633629 |
|  | *Felovia vae* | KM369883 |  |  |  |  |
|  | *Massoutiera mzabi* | AJ389533 | AJ389544 | AM407921 | AJ238388 |  |
|  |  |  |  |  |  |  |
| HYSTRICOGNATHI |  |  |  |  |  |  |
| Hystricidae | *Atherurus africanus* | HQ450774 | AY093658 |  |  |  |
|  | *Atherurus macrourus* | FJ931121 | U12451 | JF938865 | AJ251131 |  |
|  | *Hystrix africaeaustralis* | X70674 | U12448 | AF332033 |  |  |
|  | *Hystrix brachyura* | JQ991599 | AY012117 |  |  |  |
|  | *Hystrix cristata* | FJ472565 | AY093659 | JN414760 | JN415082 | AY011886 |
|  | *Hystrix indica* | AY692229 | AY093669 |  |  |  |
|  | *Trichys fasciculata* |  |  | FM162081 | AJ224675 |  |
| PHIOMORPHA |  |  |  |  |  |  |
| Bathyergidae | *Bathyergus janetta* | AF012241 | AY425843 |  |  |  |
|  | *Bathyergus suillus* | AY425913 |  | FM162080 | AJ238384 |  |
|  | *Fukomys amatus* | AF012233 | AY427021 |  |  |  |
|  | *Fukomys anselli* |  | AY427022 |  |  |  |
|  | *Fukomys bocagei* | AF012229 | AF012213 |  |  |  |
|  | *Fukomys damarensis* | AF012220 | AY427026 | FN984748 | FN984751 |  |
|  | *Fukomys darlingi* | AF012232 | AY427033 |  |  |  |
|  | *Fukomys foxi* |  | AY427036 |  |  |  |
|  | *Fukomys ilariae* |  |  |  |  |  |
|  | *Fukomys kafuensis* |  | AY427037 |  |  |  |
|  | *Fukomys mechowi* | AF012230 | AY427041 |  |  |  |
|  | *Fukomys occlusus* |  |  |  |  |  |
|  | *Fukomys ochraceocinereus* |  | AY427045 |  |  |  |
|  | *Fukomys vandewoestijneae* |  |  |  |  |  |
|  | *Fukomys whytei* |  | AY425863 | AY427046 |  |  |
|  | *Fukomys zechi* |  |  |  |  |  |
|  | *Cryptomys hottentotus* | AY425885 | AY427064 | FJ855202 | AJ251132 |  |
|  | *Cryptomys anomalous* |  | AY427054 |  |  |  |
|  | *Cryptomys holosericeus* |  | AY427051 |  |  |  |
|  | *Cryptomys natalensis* |  | M63568 |  |  |  |
|  | *Cryptomys nimrodi* |  |  |  |  |  |
|  | *Georychus capensis* | AF012243 | AY427066 | FJ855203 |  |  |
|  | *Heliophobius argenteocinereus* | KJ742646 | AY427070 | FJ855204 | AJ251133 | KJ742671 |
|  | *Heterocephalus glaber* | AF155870 | AY427075 | AF332034 | AJ251134 | AY011889 |
| Petromuridae | *Petromus typicus* | DQ139935 | M63571 | JN414761 | AJ251144 | JN633636 |
| Thryonomyidae | *Thryonomys swinderianus* | KJ742647 | NC_002658 | AF332035 | AJ224674 | KJ742672 |
|  |  |  |  |  |  |  |
| CAVIOMORPHA |  |  |  |  |  |  |
| ERETHIZONTOIDEA |  |  |  |  |  |  |
| Erethizontidae |  |  |  |  |  |  |
| Chaetomyinae | *Chaetomys subspinosus* | EU544660 |  |  |  |  |
| Erethizontinae | *Erethizon dorsatum* |  | AY012118 | AF332037 | AJ251135 | AY011887 |
|  | *Coendou bicolor* | KC463889 |  |  |  |  |
|  | *Coendou ichillus* | KC463860 |  |  |  |  |
|  | *Coendou insidiosus* | KC463861 |  |  |  |  |
|  | *Coendou melanurus* | JX312693 | JX312693 |  |  |  |
|  | *Coendou mexicanus* | KC463862 | AJ389549 |  | AJ224664 |  |
|  | *Coendou nycthemera* | KC463863 |  | FJ855212 |  |  |
|  | *Coendou prehensilis* | KC463864 |  |  |  |  |
|  | *Coendou pruinosus* | KC463873 | AF520695 | AF520663 |  |  |
|  | *Coendou quichua* | KC463880 |  |  |  |  |
|  | *Coendou roosmalenorum* | KC463882 |  |  |  |  |
|  | *Coendou spinosus* | KC463884 |  |  |  |  |
|  | *Coendou vestitus* | KC463887 |  |  |  |  |
|  | *Coendou villosus* | KC463888 |  |  |  |  |
| CAVIOIDEA |  |  |  |  |  |  |
| Caviidae |  |  |  |  |  |  |
| Caviinae | *Cavia aperea* | GU136753 | AF433908 | AF433930 |  |  |
|  | *Cavia fulgida* | GU136737 |  |  |  |  |
|  | *Cavia magna* | GU136734 | AY765986 |  |  |  |
|  | *Cavia porcellus* | AF490405 | AF433909 | AF433931 | AJ224663 | XM_003463833 |
|  | *Cavia tschudii* | GU136731 | AY012121 | FJ855206 |  | AY011890 |
|  | *Galea musteloides* | KJ742648 | AF433910 | AF433933 | KJ742608 | KJ742673 |
|  | *Galea spixii* | GU067491 | AF433913 | AF433935 |  |  |
|  | *Microcavia australis* | AF491750 | AF433915 | AF433937 |  |  |
|  | *Microcavia niata* | GU136725 |  |  |  |  |
| Dolichotinae | *Dolichotis patagonum* | GU136724 | AF433917 | AF433939 |  |  |
|  | Dolichotis salinicola | GU136723 | AF433919 | AF433940 |  |  |
| Hydrochoerinae | *Hydrochoerus hydrochaeris* | GU136721 | AF433924 | FJ855208 | AJ251137 | AY011891 |
|  | *Hydrochoerus isthmius* |  |  |  |  |  |
|  | *Kerodon acrobata* | GU477346 |  |  |  |  |
|  | *Kerodon rupestris* | GU136722 | AY765988 | AF433938 |  |  |
| Dasyproctidae | *Dasyprocta fuliginosa* | AF437784 |  |  |  |  |
|  | *Dasyprocta leporina* | AF437791 | AY093660 | FJ855207 | U31607 |  |
|  | *Dasyprocta punctata* |  | AF433921 | AF433943 | JN415079 |  |
|  | *Myoprocta acouchy* | KJ742649 | AF433922 | AF433945 | KJ742609 | KJ742695 |
|  | *Myoprocta pratti* | U34850 | AF433923 | AF433946 |  |  |
| Cuniculidae | *Cuniculus paca* | AY206555 | AF520693 | AF433928 | AJ251136 |  |
|  | *Cuniculus taczanowskii* | KJ742656 | AY012125 | AF433929 | JN415074 | AY011894 |
| CHINCHILLOIDEA |  |  |  |  |  |  |
| Chinchillidae | *Chinchilla lanigera* | AF464760 | AF520696 | AF332036 | AJ238385 | KF590658 |
|  | *Lagidium peruanum* | AY254885 |  |  |  |  |
|  | *Lagidium viscacia* | AY254886 |  | FJ855209 |  |  |
|  | *Lagidium wolffsohni* | AY227023 |  |  |  |  |
|  | *Lagostomus crassus* |  |  |  |  |  |
|  | *Lagostomus maximus* | AF245485 |  | FJ855210 |  |  |
| Dinomyidae | *Dinomys branickii* | AY254884 | AY012124 | AF520659 | AJ251145 | AY011893 |
|  |  |  |  |  |  |  |
| OCTODONTOIDEA |  |  |  |  |  |  |
| Abrocomidae | *Abrocoma bennettii* | AF244387 |  | FJ855213 | AJ251143 | JN633625 |
|  | *Abrocoma boliviensis* | KJ742657 |  |  |  |  |
|  | *Abrocoma cinerea* | AF244388 | AF520666 | AF520643 |  |  |
|  | *Cuscomys ashaninka* | KJ742658 | KJ742598 | KJ742626 | KJ742610 | KJ742683 |
| Octodontidae | *Aconaemys fuscus* | AF405351 | AF520674 | AF520657 |  |  |
|  | *Aconaemys porteri* |  | AF520671 | AF520644 |  |  |
|  | *Aconaemys sagei* | KJ742650 | AF520672 | AF520645 |  | KJ742675 |
|  | *Octodon bridgesi* | KJ742651 | AF520676 | AF520646 | KJ742611 | KJ742676 |
|  | *Octodon degus* | AF007058 | AF520678 | AF520647 |  |  |
|  | *Octodon lunatus* | AF227514 | AF520681 | AF520650 | AJ238386 |  |
|  | *Octodontomys gliroides* | AF370706 | AF520683 | AF520649 | KF590672 | KF590663 |
|  | *Octomys mimax* | GQ121097 | AF520686 | AF520652 |  |  |
|  | *Pipanacoctomys aureus* | GQ121117 | AY249753 | AY249752 |  |  |
|  | *Salinoctomys loschalchalerosorum* | KJ742652 | KJ742607 | KJ742635 | KJ742612 | KJ742684 |
|  | *Spalacopus cyanus* | AF007061 | AF520688 | AF520653 |  |  |
|  | *Tympanoctomys barrerae* | AF007060 | AF520691 | AF520655 |  |  |
| Ctenomyidae | *Ctenomys argentinus* | AF370680 |  |  |  |  |
|  | *Ctenomys australis* | AF370697 |  |  |  |  |
|  | *Ctenomys azarae* | JN791407 |  |  |  |  |
|  | *Ctenomys bergi* | AF144284 |  |  |  |  |
|  | *Ctenomys boliviensis* | AF007038 | U12446 | FJ855214 |  | FJ855214 |
|  | *Ctenomys bonettoi* | AF144286 |  |  |  |  |
|  | *Ctenomys colburni* | HM777474 |  |  |  |  |
|  | *Ctenomys conoveri* | AF007054 |  |  |  |  |
|  | *Ctenomys coyhaiquensis* | AF119112 | KF590700 | KF590678 | KF590666 | KF590659 |
|  | *Ctenomys dorbignyi* | AF500044 |  |  |  |  |
|  | *Ctenomys flamarioni* | AF119107 |  |  |  |  |
|  | Ctenomys fodax | HM777475 |  |  |  |  |
|  | Ctenomys frater | AF007045 |  |  |  |  |
|  | Ctenomys fulvus | AF370688 |  |  |  |  |
|  | Ctenomys goodfellowi | AF007050 |  |  |  |  |
|  | Ctenomys haigi | AF422920 | AF422853 |  |  |  |
|  | Ctenomys ibicuiensis | JQ389020 |  |  |  |  |
|  | Ctenomys juris | AF144275 |  |  |  |  |
|  | Ctenomys lami | HM777477 |  |  |  |  |
|  | Ctenomys latro | HM777478 |  |  |  |  |
|  | Ctenomys leucodon | AF007056 | HM544131 |  |  |  |
|  | Ctenomys lewisi | AF007049 |  |  |  |  |
|  | Ctenomys magellanicus | HM777479 |  |  |  |  |
|  | Ctenomys maulinus | AF370703 |  |  | AJ251138 |  |
|  | Ctenomys mendocinus | HM777480 |  |  |  |  |
|  | Ctenomys minutus | HM777483 |  |  |  |  |
|  | Ctenomys nattereri | HM777484 |  |  |  |  |
|  | Ctenomys occultus | HM777485 |  |  |  |  |
|  | Ctenomys opimus | AF370700 |  |  |  |  |
|  | Ctenomys pearsoni | HM777486 |  |  |  |  |
|  | Ctenomys perrensi | HM777489 |  |  |  |  |
|  | Ctenomys pilarensis | AF144265 |  |  |  |  |
|  | Ctenomys porteousi | AF370682 |  |  |  |  |
|  | Ctenomys pundit | HM777490 |  |  |  |  |
|  | Ctenomys rionegrensis | AF119103 | HM544130 |  |  |  |
|  | Ctenomys roigi | HM777492 |  |  |  |  |
|  | Ctenomys saltarius | HM777493 |  |  |  |  |
|  | Ctenomys scagliai | HM777494 |  |  |  |  |
|  | Ctenomys sericeus | HM777496 |  |  |  |  |
|  | Ctenomys sociabilis | HM777495 | HM544129 |  |  |  |
|  | Ctenomys steinbachi | AF007044 | AF520667 | AF520656 |  |  |
|  | Ctenomys talarum | AF370699 |  |  |  |  |
|  | Ctenomys torquatus | AF119111 |  |  |  |  |
|  | Ctenomys tuconax | AF370684 |  |  |  |  |
|  | Ctenomys tucumanus | AF370691 |  |  |  |  |
|  | Ctenomys yolandae | AF144285 |  |  |  |  |
| Echimyidae |  |  |  |  |  |  |
| Dactylomyinae | *Dactylomys boliviensis* | L23339 | AF422875 | JX515334 | AJ849307 | EU313298 |
|  | *Dactylomys dactylinus* | L23335 | AF422874 | KF590681 | KF590667 | EU313300 |
|  | *Dactylomys peruanus* | EU313206 |  |  |  |  |
|  | *Kannabateomys amblyonyx* | AF422917 | AF422850 |  | AJ849310 |  |
|  | *Olallamys albicauda* | KF590697 | *Fabre et al. 2017* | KF590690 | KF590673 |  |
|  | *Olallamys edax* | *this study* | *this study* |  |  |  |
| Echimyinae | *Callistomys pictus* | KJ742659 | KJ742594 | KJ742627 | KJ742614 | KJ742677 |
|  | *Diplomys caniceps* | *this study* | *this study* |  |  |  |
|  | *Diplomys labilis* | KJ742660 | *Fabre et al. 2017* | KJ742636 | KJ742613 | KJ742685 |
|  | *Santamartamys rufodorsalis* | KJ742664 | *Fabre et al. 2017* |  |  |  |
|  | *Echimys chrysurus* | L23341 | AF422877 | JX515333 | AJ251141 | EU313303 |
|  | *Echimys saturnus* | *this study* | *this study* |  |  |  |
|  | *Pattonomys occasius* | KJ742661 | Emmons & Fabre 2018 | KJ742637 |  |  |
|  | *Pattonomys semivillosus* | KJ742662 | Emmons & Fabre 2018 |  | KJ742616 | KJ742687 |
|  | *Isothrix barbarabrownae* | EU313214 | KF590701 | KF590682 | KF590668 | EU313304 |
|  | *Isothrix bistriata* | L23349 |  | JX515336 | AJ849308 | EU313307 |
|  | *Isothrix negrensis* | L23355 | AF422873 |  |  |  |
|  | *Isothrix orinoci ‡* | EU313223 | KF590702 | KF590683 | KF590669 | KF590660 |
|  | *Isothrix pagurus ‡* | EU313227 | KF590703 | KF590684 | KF590670 | KF590661 |
|  | *Isothrix sinnamariensis* | AY745734 | KF590704 | KF590685 | AJ849309 | EU313312 |
|  | *Toromys grandis* | KF590699 | Emmons & Fabre 2018 | KF590694 | KF590676 | EU313336 |
|  | *Toromys rhipidurus* | KJ742663 | Emmons & Fabre 2018 | KJ742638 | KJ742617 | KJ742686 |
|  | *Toromys albiventris* | Emmons & Fabre 2018 | Emmons & Fabre 2018 |  |  |  |
|  | *Makalata didelphoides* | L23362 | KJ742600 | KJ742639 | AJ849311 | KJ742688 |
|  | *Makalata macrura* | L23356 | AF422879 | KF590687 | AJ849312 | EU313328 |
|  | *Phyllomys blainvillii* | JF297836 | KF590706 | KF590692 | JF297734 | KF590664 |
|  | *Phyllomys brasiliensis* | EF608182 |  |  | JF297729 |  |
|  | *Phyllomys dasythrix* | JF297832 | KJ742605 | KJ742641 | JF297708 | KJ742689 |
|  | *Phyllomys lamarum* | EF608181 |  |  | JF297730 |  |
|  | Phyllomys lundi | EF608183 |  |  | JF297721 |  |
|  | Phyllomys mantiqueirensis | EF608179 |  |  | JF297720 |  |
|  | *Phyllomys nigrispinus* | JF297807 |  |  | JF297714 |  |
|  | *Phyllomys pattoni* | EF608187 | KJ742606 | KJ742642 | JF297744 | KJ742690 |
|  | Phyllomys sulinus | JF297833 |  |  | JF297710 |  |
|  | Carterodon sulcidens | KJ742666 | KJ742596 | KJ742640 | KJ742615 | KJ742678 |
|  | Clyomys laticeps | AF422918 | KJ742597 | KJ742628 | AJ849306 | KJ742679 |
|  | Euryzygomatomys spinosus | EU544667 | AF422854 | KJ742629 | AJ849319 | KJ742680 |
|  | Hoplomys gymnurus | AF422922 | AF520668 | AF520661 | JN415080 | JN633632 |
|  | Lonchothrix emiliae | AF422921 | AF422857 |  |  |  |
|  | Mesomys hispidus | KF590705 | KF590696 | KF590688 | KF590671 | KF590662 |
|  | Mesomys occultus | L23388 | AF422858 | KF590689 |  | EU313331 |
|  | Mesomys stimulax | KJ742667 | KJ742603 | KJ742630 | KJ742618 | KJ742674 |
|  | Myocastor coypus | EU544663 | AF520669 | AF520662 | AJ251140 | AY011892 |
|  | Proechimys brevicauda | *this study* | *this study* |  |  |  |
|  | Proechimys cuvieri | AJ251400 | KF590707 | KF590693 | KF590675 | KF590665 |
|  | Proechimys echinothrix | *this study* | *this study* |  |  |  |
|  | Proechimys gardneri | *this study* | *this study* |  |  |  |
|  | Proechimys goeldii | *this study* | *this study* |  |  |  |
|  | Proechimys guairae | *this study* | *this study* |  |  |  |
|  | Proechimys guyannensis | AJ251396 | *this study* |  |  |  |
|  | Proechimys hoplomyoides | *this study* | *this study* |  |  |  |
|  | Proechimys kulinae | *this study* | *this study* |  |  |  |
|  | Proechimys longicaudatus | HM544128 | HM544128 | KJ742643 | KJ742619 | KJ742681 |
|  | Proechimys mincae | *this study* | *this study* |  |  |  |
|  | Proechimys pattoni | *this study* | *this study* |  |  |  |
|  | Proechimys poliopus | *this study* | *this study* |  |  |  |
|  | Proechimys quadruplicatus | U35413 | AF422863 |  | AJ849313 |  |
|  | Proechimys roberti | *this study* | *this study* |  | AJ251139 |  |
|  | Proechimys semispinosus | *this study* | *this study* |  |  |  |
|  | Proechimys simonsi | U35414 | AF422864 | KJ742631 | AJ849320 | EU313332 |
|  | Proechimys steerei | *this study* | *this study* |  |  |  |
|  | Proechimys trinitatus | *this study* | *this study* |  |  |  |
|  | Thrichomys apereoides | EU313252 | AF422855 | JX515325 | AJ849315 | EU313334 |
|  | Thrichomys inermis | AY083343 |  |  |  |  |
|  | Thrichomys pachyurus | AY083329 |  |  |  |  |
|  | Trinomys albispinus | U34856 |  |  |  |  |
|  | Trinomys dimidiatus | U35169 | AF422867 |  | KJ742620 | KJ742682 |
|  | Trinomys eliasi | U35166 | AF422869 |  |  |  |
|  | Trinomys gratiosus | AF194281 |  |  |  |  |
|  | Trinomys iheringi | EU313254 | AF422868 | KF590695 | KF590677 | EU313337 |
|  | Trinomys paratus | U35165 | AF422866 |  | AJ849316 |  |
|  | Trinomys setosus | AF422924 | AF422871 |  | AJ849317 |  |
|  | Trinomys yonenagae | AF194295 | AF422865 |  | AJ849318 |  |
| Capromyidae |  |  |  |  |  |  |
| Capromyinae | *Capromys pilorides* | AF422915 | AF433926 | AF433950 | AJ251142 | JN633628 |
|  | *Geocapromys browni* | KJ742653 | KJ742599 | KJ742644 | KJ742621 | KJ742692 |
|  | *Geocapromys ingrahami* | KJ742668 |  |  |  |  |
|  | *Geocapromys thoracatus* | *this study* | *this study* |  |  |  |
|  | *Mesocapromys angelcabrerai* | KJ742654 | KJ742595 | KJ742632 | KJ742622 | KJ742694 |
|  | *Mesocapromys auritus* | KJ742655 | KJ742601 | KJ742633 | KJ742623 | KJ742693 |
|  | *Mesocapromys melanurus* | KJ742669 | KJ742602 |  |  | KJ742691 |
|  | *Mesocapromys nanus* | *this study* | *this study* |  |  |  |
|  | *Mysateles prehensilis* | KJ742670 | KJ742604 | KJ742634 | KJ742624 | KJ742696 |
| Plagiodontinae | *Plagiodontia aedium* | KJ742665 |  | KJ742645 | KJ742625 | KJ742697 |

**References for SI text and tables**
